# Coumarin– and Dipicolylamine–Terpenoid Hybrids as Selective Carbonic Anhydrases IX and XII Inhibitors: Mechanistic Insights and Selective Anti-Cancer Potential

**DOI:** 10.3390/ph19050717

**Published:** 2026-04-30

**Authors:** Venkatesan Saravanan, Andrea Angeli, Francesco Melfi, Nicola Amodio, Ilenia Valentino, Massimo Gentile, Ilaria D’Agostino, Kathiravan Muthukumaradoss, Gokhan Zengin, Davide Moi, Rahime Simsek, Claudiu T. Supuran, Simone Carradori

**Affiliations:** 1 School of Pharmacy, SBV Chennai, Sri Balaji Vidyapeeth, Pondicherry 607402, India; venkuu111@gmail.com; 2 Neurofarba Department, University of Florence, Sesto Fiorentino, 50019 Florence, Italy; claudiu.supuran@unifi.it; 3 Department of Pharmacy, “G. d’Annunzio” University of Chieti-Pescara, 66100 Chieti, Italy; francesco.melfi@unich.it (F.M.); simone.carradori@unich.it (S.C.); 4 Department of Experimental and Clinical Medicine, University “Magna Græcia” of Catanzaro, Campus “S. Venuta”, 88100 Catanzaro, Italy; amodio@unicz.it (N.A.); ilenia.valentino@studenti.unicz.it (I.V.); 5 Department of Pharmacy, Health and Nutritional Science, University of Calabria, 87036 Rende, Italy; massimo.gentile@unical.it; 6 Department of Pharmacy, University of Pisa, via Bonanno Pisano 6, 56126 Pisa, Italy; ilaria.dagostino@unipi.it; 7 Department of Pharmaceutical Chemistry, Faculty of Medicine and Health Sciences, SRM Institute of Science and Technology, Kattankulathur, SRM College of Pharmacy, Chengalpattu District, Tamil Nadu 603203, India; kathirak@srmist.edu.in; 8 Department of Biology, Science Faculty, Selcuk University, 42130 Konya, Türkiye; gokhanzengin@selcuk.edu.tr; 9 Dipartimento di Scienze della Vita e dell’Ambiente, Cittadella Universitaria di Monserrato, Università degli Studi di Cagliari, S.P. 8 CA, 09042 Monserrato, Italy; davide.moi@unica.it; 10 Department of Pharmaceutical Chemistry, Faculty of Pharmacy, Hacettepe University, 06100 Ankara, Türkiye; rsimsek@hacettepe.edu.tr

**Keywords:** Carbonic anhydrase, 4-chlorothymol, dipicolylamine, in silico studies, multiple myeloma, terpenoids, umbelliferon

## Abstract

**Background**: Carbonic Anhydrases (CAs) represent regulators of cell adaptation to hypoxia, pH regulation, and metabolic fitness. Among cancers, multiple myeloma (MM) is a plasma cell malignancy sustained by hypoxia-driven metabolic adaptation, extracellular acidification, and redox imbalance. Tight regulation of tumor extracellular pH, mediated by Carbonic Anhydrases IX and XII, is crucial for myeloma survival, progression, and stemness, making these isoforms attractive therapeutic targets. **Methods**: We designed and synthesized a library of terpenoid-based hybrids by derivatizing chlorothymol and 4-isopropyl-3-methylphenol with either the natural coumarin umbelliferon or the 2,2′-dipicolylamine (DPA) scaffold. This chemical strategy aimed to selectively inhibit tumor-associated CAs IX/XII through coumarin- or DPA-mediated recognition, while terpenoid fragments were introduced to enhance lipophilicity, membrane permeability, and potential redox-modulating properties. The compounds were tested by a Stopped-Flow assay for CA inhibition, in cell-based assays for antiproliferative properties and by means of several antioxidant assays. **Results**: The most active compounds, connecting the coumarin core to a terpenoid tail, inhibited the targeted CAs in the nanomolar range, showing up higher selectivity over off-target isoforms (I and II). In studies performed on MM cell lines, selected derivatives reduced viability (IC_50_ = 15.8–85.4 µM) and displayed favorable selectivity over normal cells. In silico investigations suggested that the compounds were able to interact selectively with the target enzymes. **Conclusions**: Collectively, these results support a dual-targeting strategy in which selective inhibition of tumor-associated CAs, combined with redox modulation, interferes with adaptive mechanisms of MM cells, providing a rational framework for the development of multifunctional agents against metabolically resilient hematological malignancies.

## 1. Introduction

Cancer remains one of the leading causes of death worldwide, accounting for 20 million new cancer cases and 9.7 million deaths in 2022 and projected to increase to 53.5 million cases within the next five years [1]. Despite advances in early diagnosis, surgery, radiotherapy, and systemic chemotherapies, drug resistance continues to be one of the biggest challenges in oncology, despite the release of new drugs [2]. While resistance mechanisms arising from target alterations and tumor cell heterogeneity are still difficult to overcome, strategies that address cancer-protective mechanisms in the microenvironment offer more accessible pharmacological opportunities [3,4,5,6].

The tumor microenvironment (TME) plays a pivotal role in cancer progression and treatment response [7,8]. Indeed, rapid proliferation and altered energy pathways, as well as hypoxia and the acidification that follows, trigger a cascade of molecular pathways that enable cancer cells to adapt and survive in these TME conditions, leading to invasion, migration, and drug resistance. In order to counteract the large amounts of acidic byproducts generated and maintain intracellular pH homeostasis, cells developed specific oncogenic mechanisms [9,10]. Although hypoxia often accompanies acidosis, low pH can also develop under normoxic conditions due to the preference of many tumor cells for glycolysis, known as the Warburg effect [11,12,13]. Indeed, during oxidative metabolism, the pentose phosphate pathway, and other biochemical processes carbon dioxide molecules produced strongly contribute to extracellular acidity [13,14].

Indeed, it is rapidly interconverted to bicarbonate, along with proton generation, by Carbonic Anhydrases (CAs; EC 4.2.1.1), a family of zinc metalloenzymes that function as pH buffers both inside and outside cells. Among the twelve catalytically active human isoforms, membrane-associated CAs IX and XII are relevant for their role in cancer [15,16]. Their expression is regulated by hypoxia-inducible factor-1α (HIF-1α) and is strongly upregulated in hypoxic regions, where they contribute to maintaining intracellular alkalinity and extracellular acidification [17,18]. Although first discovered in solid tumors, overexpression of these cancer-associated CA isoforms has also been reported in Multiple Myeloma (MM) [19,20]. This disease is a clonal plasma cell malignancy characterized by uncontrolled proliferation within the bone marrow microenvironment, progressive bone destruction, metabolic reprogramming, and the development of drug resistance, resulting in high mortality [21,22,23]. Unfortunately, although recent therapies have prolonged overall survival, relapse and refractory disease remain major clinical challenges in MM patients, largely due to the remarkable adaptive capacity of myeloma cells to hypoxic, acidic, and oxidative stress conditions [24,25,26]. Furthermore, the intrinsic hypoxia and high metabolic activity of this niche strongly support MM progression [27]. Consequently, CA inhibitors (CAIs) have recently gained attention as promising anti-MM agents, selectively targeting cancer-associated isoforms [28,29,30].

In parallel, MM cells exhibit elevated basal levels of reactive oxygen species (ROS), resulting from high immunoglobulin synthesis, intense endoplasmic reticulum activity, mitochondrial metabolism, and oncogenic signaling pathways. While excessive ROS accumulation is cytotoxic, moderate ROS levels promote survival signaling and genomic instability [31,32]. To maintain viability, myeloma cells upregulate antioxidant defense mechanisms, including NRF2-driven transcriptional programs and glutathione-dependent detoxification systems [33]. Redox homeostasis is therefore a critical determinant of MM cell survival. Importantly, pH regulation and oxidative stress adaptation are functionally interconnected [34,35]. Hypoxia simultaneously enhances hCAs IX and XII expression and reshapes mitochondrial metabolism, influencing intracellular ROS levels. By preserving intracellular alkalinity, carbonic anhydrases indirectly contribute to redox tolerance, protecting malignant plasma cells from acidification-induced oxidative stress. Conversely, selective inhibition of hCAs IX and XII may impair intracellular buffering capacity, exacerbate metabolic stress, and increase oxidative vulnerability. Within this framework, compounds capable of combining carbonic anhydrase inhibition with antioxidant or redox-modulating properties may disrupt multiple adaptive axes of MM survival [36]. Rather than acting as simple cytoprotective antioxidants, such agents may interfere with the finely tuned redox balance required by highly secretory plasma cells, particularly under hypoxic and acidic conditions. Targeting both pH regulation and redox homeostasis thus represents a rational strategy to counteract metabolic resilience and therapeutic resistance in MM [37].

In the search for interesting chemotype, endowed with high isoform selectivity and inhibitory activity, we focused our attention on the well-characterized coumarin scaffold since compounds incorporating this moiety have exhibited broad pharmacological properties, especially in oncology [38,39,40] along with antiviral, anticoagulant, anti-inflammatory, antimutagenic, anti-tubercular, CNS stimulant and fungicidal fields [41,42,43,44]. In particular, several coumarin-based derivatives displayed selective inhibition of the tumor-associated hCA IX and XII isoforms, thus limiting the side effects associated with pan-CA inhibitors [45,46,47]. In addition, we have recently explored the potent inhibitory activity of umbelliferon-terpene hybrids connected by using methylene spacers or triazole linkers. These new compounds demonstrated nanomolar inhibitory activity against these targets, as also confirmed by molecular modeling and dynamics simulations which highlighted their peculiar mechanism of action [47]. To further explore the chemical space around the terpenoid tail and to enlarge this library of biologically active hybrids, we have designed the introduction of new terpenoid tails connected to the coumarin core and the substitution of this well-known CA-interacting group with alternative zinc binders with the aim of outperforming single pharmacophores. In this context, previous studies have shown that dipicolylamine (DPA) and its derivatives can effectively inhibit other zinc-containing enzymes (e.g., metalloproteases and metallo-β-lactamases) [48,49] by forming a strong Zn^2+^-DPA complex and establishing π–π stacking interactions through its pyridine rings. This hybridization strategy [50] was carried out by exploiting two different linkers: aliphatic chains for conformational freedom [51] and 1,2,3-triazoles for rigidity and improved metabolic stability and aqueous solubility [52,53].

## 2. Results and Discussion

### 2.1. Rationale of the Study

**Figure 1:**
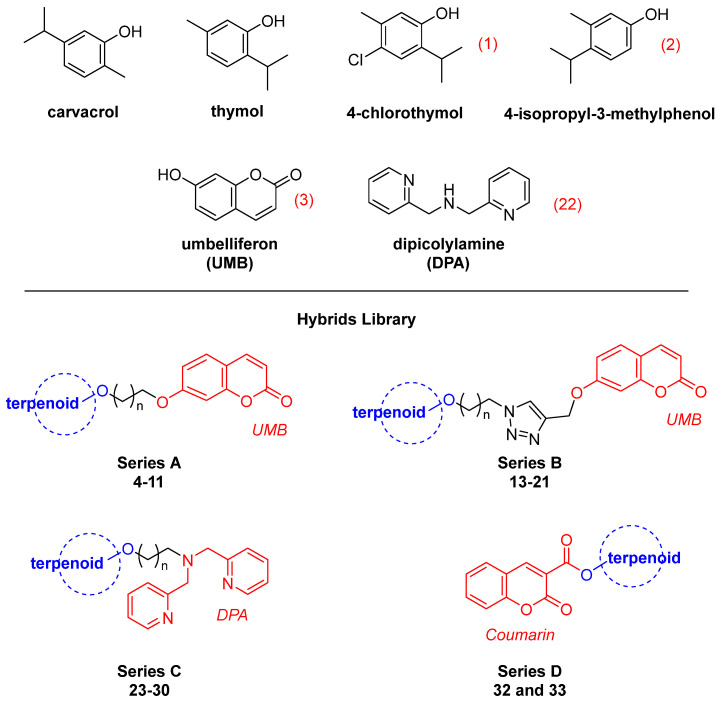
Chemical structures of carvacrol, its isomers thymol and 4-isopropyl-3-methylphenol, and the derivative 4-chlorothymol, alongside the structures of **UMB** and **DPA**. General structures of the hybrid series A, B, C, and D developed in this study.

Recently, increasing efforts have been directed toward natural phenolic monoterpenes such as carvacrol and thymol (Figure 1), whose structural modification has enabled to expand their therapeutic potential, including anticancer applications [54,55,56,57].

In contrast, synthetic analogues such as the regioisomer 4-isopropyl-3-methylphenol (*o*-cymen-5-ol) and the derivative 4-chlorothymol (Figure 1) remain largely unexplored in the context of cancer-targeted drug development. To address this gap, we designed and synthesized a new library of hybrids in which these terpenoids were conjugated either with the natural coumarin umbelliferon [58] (**UMB**) or with dipicolylamine (**DPA**), a well-established zinc-chelating moiety (K_d_ ≈ 10^−8^ M [59,60]) (Figure 1). This strategy was intended to broaden chemical diversity while enabling modulation of physicochemical and biological properties of **UMB** and investigate **DPA** for the first time as CA-inhibiting chemotype. Specifically, the library of derivatives developed was composed of four different series, namely A, B, C, and D (Figure 1).

Series A: Smaller hybrids in which the terpenoid and **UMB** portions are directly connected via *O*-alkylation of the terpenoid phenols. This design allowed investigation of the effects of linker length and terpenoid scaffold on CAs IX and XII inhibition.

Series B: Hybrids in which the terpenoid and **UMB** are linked through a 1,2,3-triazole formed via the versatile click chemistry, introducing a rigid and polar linker to modulate binding orientation and selectivity [61]. This nitrogen-containing ring is well-known as a bioisostere of the amide group, a good player in establishing H-bonds and π-π interactions, and tolerant to metabolism as well as pH changes.

Series C: Conjugates of terpenoid and **DPA**, in which phenolic terpenoids are directly attached to DPA, combining a lipophilic terpenoid tail with a zinc-chelating pharmacophore to explore an alternative mode of CA inhibition.

Series D: Coumarin ester derivatives in which the terpenoid core is linked to coumarin via esterification, designed to probe the effects of a more flexible linker on CA binding and to explore alternative structural arrangements for modulating biological activity.

### 2.2. Synthesis of the New Derivatives

**Scheme 1:**
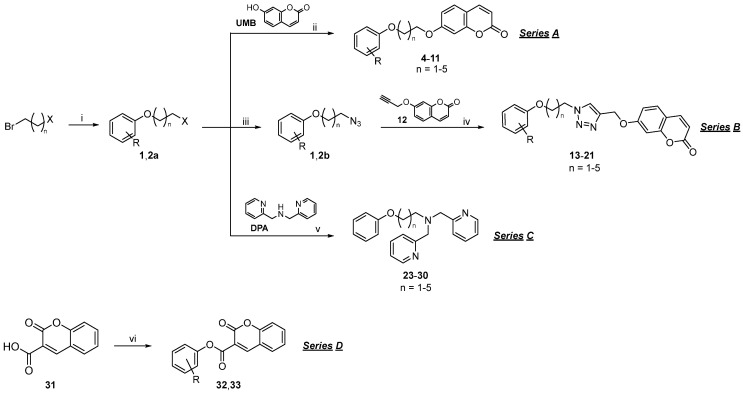
Synthesis of the derivatives library. Reagents and conditions: (i) 4-chlorothymol (**1**) or 4-isopropyl-3-methylphenol (**2**), K_2_CO_3_, ACN or cyrene, microwave, 90 °C, 2–3 h; (ii) K_2_CO_3_, ACN, microwave, 90 °C, 2–3 h; (iii) NaN_3_, DMF, room temperature, 2 h; (iv) CuSO_4_ × 5H_2_O, sodium ascorbate, H_2_O/*t*BuOH 1:1, room temperature, 16 h; (v) K_2_CO_3_, ACN/H_2_O, 100 °C, 16 h; (vi) 4-chlorothymol (**1**) or 4-isopropyl-3-methylphenol (**2**), DCC, DMAP, dry DCM, room temperature, 24 h.

The derivatives library was synthesized following a straightforward approach, as outlined in Scheme 1.

Specifically, series A derivatives were prepared via two consecutive microwave-assisted nucleophilic substitutions. The terpenoid moieties, 4-chlorothymol (**1**) or 4-isopropyl-3-methylphenol (**2**), were first reacted with an appropriate alkyl dihalide to generate intermediates **1a** and **2a**. An initial attempt to perform this reaction under greener conditions using cyrene, a biocompatible and biodegradable solvent [62,63,64], resulted in low yields and complicated purification. Consequently, the reactions were carried out under standard conditions using acetonitrile (ACN) as solvent in the presence of potassium carbonate. The resulting halogenated intermediates **1a** and **2a** were then coupled with **UMB** (**3**) via *O*-alkylation to afford the series A ether derivatives **4**–**11** (Scheme 1).

For series B, **1a** and **2a** were first converted into the corresponding azides **1b** and **2b** in mild conditions and these azides then underwent Huisgen’s copper-catalyzed azide–alkyne cycloaddition (CuAAC) [65] with *O*-propargyl umbelliferon **12**, which was previously prepared by treating **UMB** (**3**) with propargyl bromide in ACN or cyrene, yielding the clicked series B derivatives **13**–**21** (Scheme 1). Series C derivatives **23**–**30** were obtained by reacting **1a** and **2a** directly with **DPA** (**22**) in a mixture of ACN and water, whereas series D compounds **32** and **33** were prepared via Steglich esterification [66] using commercially available 3-cumarin carboxylic acid **31** (Scheme 1).

### 2.3. CA Inhibitory Assays

The whole library was tested on the targeted enzymes CAs IX and XII and the physiologically relevant CAs I and II and the inhibition constants are reported in Table 1 with the parent compounds 4-chlorothymol (**1**), 4-isopropyl-3-methylphenol (**2**), **UMB** (**3**), **DPA** (**22**) and coumarin-3-carboxylic acid (**31**).

### 2.4. Structure-Activity Relationships

The structural modification of scaffolds chlorothymol **1** (K_I_ = 34.7–66.5 µM) and 4-isopropyl-3-methylphenol **2** (K_I_ = 34.7–66.5 µM) led to a marked improvement in inhibitory activity against hCA IX and XII compared to the corresponding unmodified parent compounds, especially for series A and B compounds in which the terpenoid tail was linked via a spacer to a natural coumarin scaffold (Figure 1), highlighting the effectiveness of the designed derivatives in enhancing target selectivity and potency. These outcomes are in agreement with the proposed hybridization approach of two core nuclei as also reported for other coumarins as CA inhibitors [68].

All compounds in series A demonstrated notable selectivity toward cancer-related isoforms hCA IX and XII, as evidenced by their weak inhibition of the ubiquitous isoforms hCA I and II (K_I_ > 100,000 nM). For these derivatives, no clear trend in hCA IX inhibitory activity was observed based solely on the presence of either the chlorothymol (**1**) or the 4-isopropyl-3-methylphenol (**2**) moiety. However, when considering derivatives with a two-methylene linker, the chlorothymol-based scaffold (compound **4**) appears to be more favorable, displaying a K_I_ of 30.4 nM compared to 93.8 nM for its counterpart, compound **5**. In contrast, across the remaining derivatives, the 4-isopropyl-3-methylphenol scaffold (**2**) seems to enhance the inhibitory potency against hCA IX. This is exemplified by compounds **7** (K_I_ = 22.8 nM), **9** (K_I_ = 29.2 nM), and **11** (K_I_ = 29.7 nM), which rank among the most active compounds in the series against this isoform. Regarding hCA XII, the inhibition constants for series A compounds are generally lower than the other series and do not display a consistent structure-activity relationship. Notably, compounds **4**, **7**, and **9** exhibit the best performance within this series, with K_I_ values ranging between 81.4 and 86.8 nM.

For the compounds of series B, the inhibition constants were generally higher for both hCA IX and, more notably, for hCA XII, with the exception of compound **20**, which features the chlorothymol moiety and a six-methylene linker connected to the triazole ring. Within this series, the most potent inhibitor of hCA IX was compound **13** (K_I_ = 27.6 nM); however, this compound exhibited significantly lower potency against hCA XII (K_I_ = 418.4 nM). As with series A, no consistent structure-activity trend could be identified for hCA IX inhibition among the series B derivatives, aside from compounds **13** and **20** (K_I_ = 32.0 nM), both of which incorporate the parent scaffold **1**. The remaining compounds displayed K_I_ values for hCA IX ranging between 42.3 and 200.0 nM. In contrast, hCA XII inhibition was generally weaker across the series, with the exception of compounds **15** (K_I_ hCA IX = 178.3 nM vs. K_I_ hCA XII = 82.4 nM), **20** (K_I_ hCA IX = 32.0 nM vs. K_I_ hCA XII = 9.3 nM), and **21** (K_I_ hCA IX = 200.0 nM vs. K_I_ hCA XII = 71.9 nM), which exhibited enhanced potency against this isoform when compared to their activity against hCA IX. Moreover, **20** resulted to be the best-in-class molecule in the inhibition of hCA XII.

As regards **DPA** (**22**) and its derivatives (**23**–**30**), none of these compounds exhibited inhibitory activity against any of the tested hCA isoforms, despite the well-established metal-chelating properties of DPA and the data in the literature regarding their antiproliferative effects [69,70]. This lack of activity may be attributed to suboptimal orientation or insufficient binding interactions within the active site of the enzyme, suggesting that the **DPA** scaffold alone is not adequate to ensure effective inhibition, possibly due to steric or electronic factors that prevent productive coordination with the catalytic zinc ion. As described below, ligand-receptor interaction diagrams further support the lack of consistent hydrogen bonds, π–π stacking interactions, or direct coordination to the metal center, in contrast to the coumarin-based reference compounds.

Lastly, the coumarin-ester derivatives **32** and **33** proved to be more active than the parent compound **31**, exhibiting more consistent inhibitory profiles, thus confirming that also the prodrug approach could be a relevant strategy [71]. Both derivatives showed improved activity against hCA IX, with K_I_ values of 2295 nM and 2934 nM, respectively, while also displaying moderate inhibition of hCA XII, with K_I_ values of 4589 nM and 6940 nM, respectively.

### 2.5. Molecular Docking and Dynamics

The coumarin moiety undergoes enzymatic hydrolysis by CA esterase activity, yielding the corresponding *E*/*Z* 2-hydroxycinnamic acid derivatives, which act as suicide inhibitors by blocking the entrance of the active site [72]. In light of this, docking calculations for the selected compounds were conducted on their hydrolyzed forms, considering both the *E* and *Z* isomers. This dual-isomer approach reflects the possible conformational outcomes upon lactone ring hydrolysis and allows for a more accurate assessment of their binding affinity and selectivity toward hCA IX and XII. Active site analysis of the target proteins was also performed. In hCA XII, the reference ligand acetazolamide (AAZ) interacts with key residues including Zn901, His94, His96, His119, and Thr199. Similarly, in hCA IX, the co-crystallized sulfonamide ligand is stabilized via interactions with Zn301, His94, His96, His119, and Thr199, with additional polar contacts observed at the entrance region involving Gln67 and Arg60.

In order to elucidate the molecular determinants responsible for the selective inhibition of neoplasia-associated hCA IX and hCA XII isoforms, docking calculations were performed on the hydrolyzed forms (both *E* and *Z* isomers) of the most active coumarin derivatives previously tested experimentally, namely **4**, **7**, **9**, **11**, **13**, and **20**. Among these, **20** and **9** demonstrated the most favorable binding energies, suggesting a higher probability of interaction and stabilization within the CA active sites.

**Figure 2:**
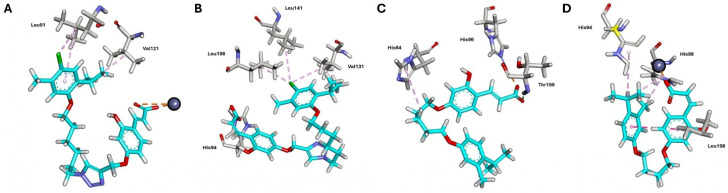
Predicted binding interactions of hydrolyzed coumarin derivatives with hCA IX. Ligand–protein interaction diagrams of (**A**) **20**(*E*), (**B**) **20**(*Z*), (**C**) **9**(*E*), and (**D**) **9**(*Z*) in the active site of hCA IX (PDB ID: 6G9U). Ligand carbons are shown in cyan and protein carbons in gray; oxygen in red, nitrogen in blue, and the zinc ion as a sphere. Purple dashed lines indicate hydrogen bonds and coordination interactions.

Specifically, for hCA IX, as shown in Table 2, the *E*-isomer of **20** exhibited the strongest binding affinity, marginally outperforming **9**(*E*), followed by **9**(*Z*) and **20**(*Z*). In the case of hCA XII, the docking scores indicated that **9**(*E*) maintained the most favorable interaction, followed by **9**(*Z*), **20**(*Z*) and **20**(*E*). These data suggest that while both compounds possess significant affinity for the tumor-associated isoforms, the hydrolyzed form of **9** presents a more balanced dual-isoform inhibition profile, whereas the hydrolyzed form of **20** appears to be preferentially selective toward hCA IX. To better understand the molecular basis of these interactions, a detailed analysis of the binding modes was carried out for both compounds in their *E* and *Z* hydrolyzed forms using ligand–protein interaction diagrams generated within the active sites of hCA IX and hCA XII. In hCA IX, **20**(*E*) exhibited a well-defined orientation wherein the resorcinol moiety formed stabilizing hydrogen bonds with Thr199 and Gln67 and was further anchored by hydrophobic contacts with residues such as Val121 and Leu198. Additional interactions were observed with the backbone of Gln67, and a favorable polar interaction with Arg60 at the entrance of the active site, highlighting its potential for selective hCA IX inhibition. In contrast, **20**(*Z*) adopted a less optimal pose, lacking the strong polar network seen in the E isomer, particularly missing the stabilizing interaction with Gln67, which likely accounts for its reduced binding affinity. **9**(*E*), on the other hand, demonstrated a highly stabilized binding configuration, characterized by a triad of hydrogen bonds involving Thr199, His94, and Gln67, along with π–π interactions with His96. The spatial fit of the hydroxycinnamic acid scaffold allowed deep penetration into the catalytic pocket, while maintaining coordination with key zinc-chelating residues, closely mimicking the behavior of classical sulfonamide inhibitors. **9**(*Z*) preserved several of these interactions, but a subtle reorientation of the aromatic moiety reduced the extent of hydrophobic packing, leading to a slight decrease in docking score relative to the *E* isomer (Figure 2).

**Figure 3:**
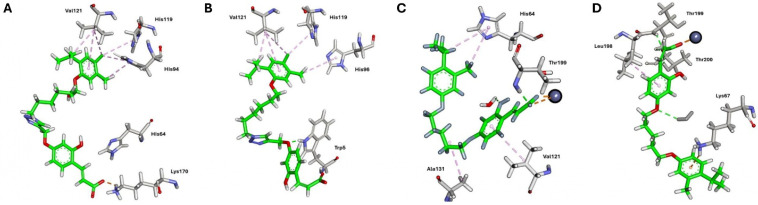
Predicted binding interactions of hydrolyzed coumarin derivatives with hCA XII. Ligand–protein interaction diagrams of (**A**) **20**(*E*), (**B**) **20**(*Z*), (**C**) **9**(*E*), and (**D**) **9**(*Z*) docked into the active site of hCA XII (PDB ID: 1JD0). Ligand carbons are shown in green and protein carbons in gray; oxygen atoms are shown in red, nitrogen in blue, and the zinc ion as a sphere. Purple dashed lines indicate hydrogen bonds and coordination interactions.

When docked into the active site of hCA XII, **9**(*E*) again showed the most favorable pose, forming strong interactions with Thr199, His119, and the catalytic zinc ion (Zn901), a hallmark of effective CA inhibition. This orientation was further stabilized by hydrogen bonding with His94 and close proximity to hydrophobic residues lining the catalytic cleft. **9**(*Z*) retained some of these interactions, particularly with Thr199 and the zinc-coordinating residues, but with a slightly altered geometry that reduced its overall binding energy. **20**(*Z*) and **20**(*E*), on the other hand, exhibited comparatively weaker interaction patterns within the hCA XII pocket (Figure 3).

The *E* isomer of hydrolyzed **20**, despite being the top binder in hCA IX, failed to establish consistent hydrogen bonds with the catalytic triad in hCA XII and lacked interaction with Thr199 and His94, resulting in a lower docking score. The *Z* isomer showed marginally improved hydrogen bonding but did not achieve deep penetration into the active site nor effective zinc coordination, which likely explains its diminished affinity. These differences highlight the importance of both stereochemistry, and substituent positioning on the hydrolyzed coumarin scaffold in achieving isoform-specific interactions. To further assess the structure-activity relationship and binding behavior of different chemotypes, we compared the docking profiles of coumarin-based compounds with a set of **DPA**-based analogs. Our analysis revealed a clear distinction in binding affinity and interaction patterns at the hCA IX active site. The coumarin derivatives, particularly **9**(*Z*) and **5**(*E*), exhibited stronger binding, with docking scores of −6.812 and −6.564 kcal/mol, respectively. In contrast, the **DPA**-based compounds (e.g., **23**, **27**, **24**, and **25**) showed weaker docking scores, ranging from −4.676 to −4.120 kcal/mol. Detailed binding mode analysis indicates that these compounds generally fail to engage in direct coordination with the zinc ion, a critical determinant of strong and selective inhibition. For instance, **23**, despite being the most favorable among the **DPA** set, adopts an orientation that positions the ligand away from the zinc-binding pocket, likely due to steric constraints imposed by a flexible linker and the absence of a suitable metal-chelating group. Conversely, the **DPA**-based molecules often contain tertiary amines and pyridyl rings, which are suboptimal for zinc coordination, especially when not properly oriented or sterically hindered. Docking poses frequently show these moieties remaining solvent-exposed or interacting with peripheral residues rather than penetrating the catalytic site. Ligand–receptor interaction diagrams (Figure S1) further confirm the absence of consistent hydrogen bonding, π–π stacking, or direct metal interactions, compared to the coumarin-based references. These findings align with in vitro data showing poor inhibitory activity for **DPA** compounds, underscoring the importance of scaffold design.

**Figure 4:**
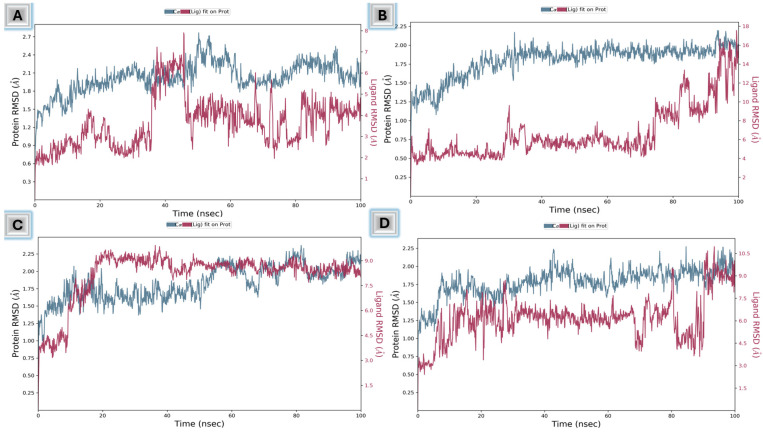
Root mean square deviation (RMSD) analysis of **20**–CA complexes over a 100 ns MD simulation. Time evolution of protein backbone RMSD (blue, left *Y*-axis) and ligand RMSD (red, right *Y*-axis) for: (**A**) **20**(*Z*) with hCA IX, (**B**) **20**(*Z*) with hCA XII, (**C**) **20**(*E*) with hCA IX, and (**D**) **20**(*E*) with hCA XII.

To further assess the conformational stability and binding persistence of the top docked complexes, molecular dynamics (MD) simulations were conducted for the hydrolyzed *E* and *Z* isomers of **20** in complex with hCA IX and hCA XII over a 100 ns trajectory. The RMSD profiles of the protein backbone and ligand (fit on protein) were evaluated to monitor structural equilibration (Figure 4). In the **20**(*E*)–hCA IX complex (Figure 4C), the protein RMSD stabilized around 1.8 Å after 10 ns and remained consistent throughout the simulation, while the ligand RMSD fluctuated minimally (2.5–4.0 Å), indicating strong retention and a stable binding orientation. In contrast, the **20**(*Z*)–hCA IX complex (Figure 4A) showed slightly more fluctuation, with the protein RMSD averaging ~2.0 Å and the ligand RMSD rising above 4.0 Å intermittently, suggesting less optimal anchoring of the *Z* isomer. For hCA XII, both **20**(*Z*) (Figure 4B) and **20**(*E*) (Figure 4D) complexes maintained protein RMSD values around 2.0 Å, but the ligand RMSDs were notably higher, particularly in the *Z* isomer, which showed excursions above 8.0 Å near the end of the simulation, indicating weaker binding and potential drift from the active site.

**Figure 5:**
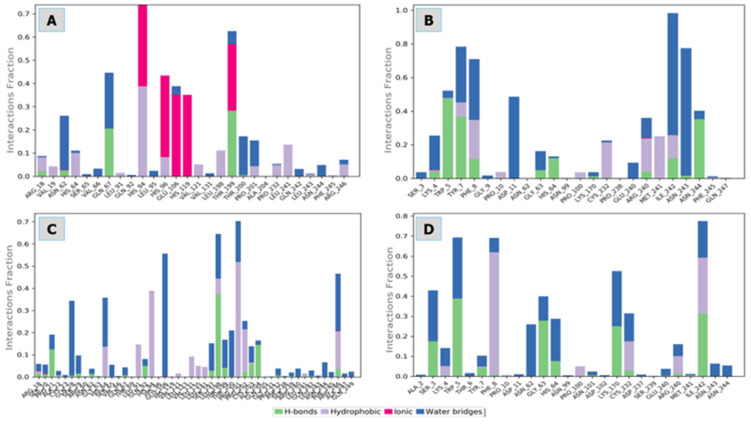
Protein–ligand interaction fractions during 100 ns MD simulations for **20**–CA complexes. Interaction types (color-coded) include hydrogen bonds (green), hydrophobic interactions (purple), ionic bonds (pink), and water bridges (blue), calculated as fractional occupancy across the simulation time frame. (**A**) **20**(*Z*) with hCA IX, (**B**) **20**(*Z*) with hCA XII, (**C**) **20**(*E*) with hCA IX, and (**D**) **20**(*E*) with hCA XII.

Post-simulation interaction analysis (Figure 5) corroborated these findings by quantifying the frequency and persistence of ligand–protein contacts. In hCA IX, **20**(*E*) (Figure 5C) established robust hydrogen bonding and hydrophobic interactions with critical residues such as Thr199, His94, Leu198, Val121, and Gln67, along with multiple water bridges, indicative of a tightly anchored and well-distributed binding mode. The *Z* isomer (Figure 5A) showed dominant ionic and water-bridged interactions—especially with Gln67 and Thr200—but lacked the consistent hydrophobic packing of its *E* counterpart. In hCA XII, **20**(*E*) (Figure 5D) formed moderate hydrogen bonds and water-mediated contacts with Thr199, His119, and Asn62, but failed to engage deeply with core catalytic residues such as His94. The *Z* isomer (Figure 5B) relied more heavily on transient water bridges involving Val143 and Ser131, consistent with its higher ligand RMSD and reduced affinity. Collectively, these results highlight that **20**(*E*) forms a more stable, persistent, and deeply embedded complex with hCA IX, reinforcing its potential as a stereoselective, non-classical inhibitor with preferential hCA IX targeting properties.

### 2.6. Cell-Based Assay on Multiple Myeloma (MM) Cells

Building on previous findings showing that MM cells overexpress hCA IX and hCA XII isoenzymes, and that their targeting can elicit anti-MM activity [21], we investigated the in vitro effects of the most potent and representative CA inhibitors (**7**, **9**, **13**, **20**) and their precursors (**1**–**3**) on the viability of MM cell lines, including both Bortezomib-sensitive and Bortezomib-resistant isogenic models (Table 3). Bortezomib is a well-established proteasome inhibitor (PI) used for the treatment of MM across both newly diagnosed and relapsed/refractory disease settings.

**Figure 6:**
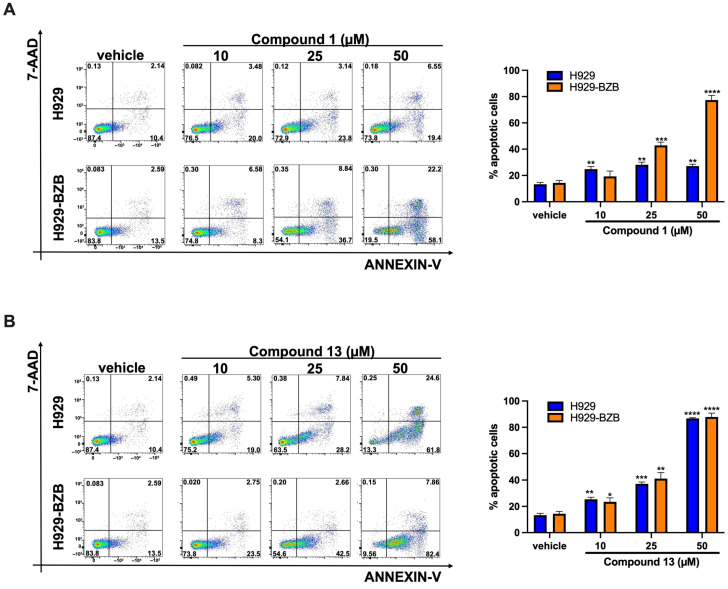
Flow cytometry analysis of Annexin V/7-AAD-stained H929 and H929-BZB cells, 48 h after treatment with compound **1** (**A**) or compound **13** (**B**); DMSO (vehicle) was used as control. The percentages of viable cells (lower left panel), early apoptotic cells (lower right panel), and late apoptotic cells (upper right panel) are shown in each plot and reported as histogram, bars. Statistical significance was calculated using unpaired Student’s *t*-test. * *p* < 0.05; ** *p* < 0.01; *** *p* < 0.00; **** *p* < 0.0001.

IC_50_ values were determined for all compounds, with lower IC_50_ values observed for compound **13**, chlorothymol (**1**), and compound **7**. Analysis of the effects of these compounds on HEK293 cells—a model of immortalized, non-cancerous kidney cells [73]—revealed two- to threefold higher IC_50_ values for **13** and **1**, indicating superior selectivity index (SI) toward AMO and H929 cancer cells (IC_50_ and Selectivity Index values in Table S1), whereas compound **7** displayed a limited selective toxicity (SI < 1). This trend was also evident for one BZB-sensitive cell line (H929-BZB) in the case of compound **13** (27.5 µM vs. 34.6 µM, SI = 1.40) and **9** (70.0 µM vs. >200 µM, SI >2.85). Moreover, the parent compound **2** was also very selective toward MM cell lines (SI higher than 2.18 for three cell lines). The selected compounds lost efficacy partially when moving from the enzymatic assay to the cell-based experiments, likely for a more complex environment, especially for transmembrane isoforms. Other factors related to the acidification of the extracellular milieu, cell metabolism and composition of the cell secretome could have an impact. Notably, fluorescence-activated cell sorting (FACS) analysis demonstrated that treatment with both chlorothymol (**1**) and compound **13** induced pronounced pro-apoptotic effects, as evidenced by a significant increase in Annexin V/7-AAD-positive cell populations (Figure 6). Importantly, in the case of compound **1**, these effects were more pronounced in BZB-resistant cells than in parental cells, indicating enhanced efficacy against drug-resistant phenotypes. In contrast, compound **13** showed comparable dose-dependent activity in both MM cell lines. Altogether, these findings suggest that both compounds exert anti-MM activity with a potentially favorable toxicity profile toward healthy cells.

### 2.7. Antioxidant Activity

To further assess a multi-target action of these compounds and keeping into consideration the properties of the parent compounds [74,75] used for the hybridization strategy, our aim was also to study the antioxidant ability of these scaffolds. This ability can be more related to the intrinsic chemical backbone and could enhance the antiproliferative effect of the derivatives [76]. An alteration of the oxidative state is not only responsible for the onset and progression of MM, but it also appears essential for the failure of therapeutic response and chemoresistance occurrence [77]. The term ‘antioxidant’ refers to substances that protect against free radical attacks, which are a key trigger of chronic and degenerative diseases, including cancer (e.g., multiple myeloma) [78]. Then, the tested compounds were examined for antioxidant properties with different test systems including radical quenching (ABTS and DPPH), reducing power (CUPRAC and FRAP) and metal chelating (Table 3).

The DPPH and ABTS assays are the most common methods used to evaluate the radical scavenging ability of antioxidants. As can be seen, the strongest DPPH and ABTS radical scavenging ability was found in compound **23** with the values of 13.12 mg TE/g and 92.44 mg TE/g, respectively. While all of the tested compounds exhibited an ABTS scavenging ability, eleven of them did not display any scavenging activity against the DPPH radical. Compounds **24** and **25** displayed a good radical scavenging activities as compared to other compounds confirming the beneficial role of the **DPA** group for this property. The electron-donating ability is also considered one of the most important antioxidant mechanisms, contributing to the closure of the electron gap in free radicals. The CUPRAC and FRAP assays are based on the reduction of Cu^2+^ to Cu^+^ and Fe^3+^ to Fe^2+^ through the electron-donating ability of antioxidant compounds. From Table 4, the best cupric and ferric reducing ability was again provided by compound **23** with the values of 286.38 mg TE/g and 85.76 mg TE/g, respectively. Among the tested compounds, **29** exhibited the lowest electron-donation ability in the CUPRAC and FRAP assays. The chelation of transition metals is closely linked to the management of the production of hydroxyl radicals in the Fenton reaction. Therefore, a compound that can chelate transition metals, particularly iron, can inhibit the production of hydroxyl radicals. Similar to other antioxidant assays trend, the best metal chelation ability was found in **23** with 48.12 mg EDTAE/g, followed by **26** (44.79 mg EDTAE/g) and **27** (42.61 mg EDTAE/g), respectively, all belonging to the **DPA**-based hybrids. Collectively, this ancillary property was more pronounced for **DPA**-based compounds, despite some coumarin-based compounds could benefit from this ability for their antiproliferative effect on MM.

## 3. Materials and Methods

### 3.1. Synthesis of the Compound Library

#### 3.1.1. General Chemistry

All commercially available chemicals and solvents were used as purchased. Chromatographic separations were performed on columns packed with silica gel (230–400 mesh, for flash technique). Reaction monitoring was performed through thin-layer chromatography (TLC) by using 0.2 mm-thick silica gel–aluminum-backed plates (60 F_254_). TLC spot visualization was performed under short and long wavelength (254 and 365 nm, respectively) ultra-violet irradiation and stained with ninhydrin or basic permanganate. ^1^H and ^13^C NMR were recorded on a spectrometer operating at 300 and 75 MHz, respectively. Spectra are reported in parts per million (*δ* scale) and internally referenced to the CDCl_3_ and MeOD, DMSO-*d*_6_ signal, respectively, at *δ* 7.26, 3.31 and 2.50 ppm. Chemical shifts for carbon are reported in parts per million (*δ* scale) and referenced to the carbon resonances of the solvent (CDCl_3_ at *δ* 77.0, MeOD at δ 49.0 and DMSO-*d*_6_ at δ 39.0). Data are shown as follows: chemical shift, multiplicity (s = singlet, d = doublet, t = triplet, q = quartet, m = multiplet and/or multiplet resonances, br = broad signal), integration and coupling constants (*J*) in Hertz (Hz). The ^1^H and ^13^C spectra confirmed the anticipated number of hydrogens for each compound, respectively. Melting points were measured on a Stuart^®^ melting point apparatus SMP1 (Fisher Scientific Italia, Segrate (MI), Italy) and are uncorrected (temperatures are reported in °C). Elemental analyses for C, H, and N were recorded on a Perkin-Elmer 240 B microanalyzer (PerkinElmer Italia Spa, Milan, Italy) and the analytical results are within ± 0.4% of the theoretical values for all compounds. Microwave-assisted reactions were performed with Biotage^®^ Initiator+ (Biotage Sweden AB, Uppsala, Sweden) in a 10-mL vial suitable for an automatic single-mode reactor (2.45-GHz high-frequency microwaves, power range 0–300 W). The internal vial temperature was strictly controlled by an IR sensor.

#### 3.1.2. Synthetic Procedure and Characterization Data

##### General Procedure for the Synthesis of Compounds **4**–**11** (Series A)

The proper alkyl halide (1.5 eq) was added to the colorless solution of the specific phenol (**1** and **2**) (1.0 eq) in acetonitrile or cyrene (0.4 M). Then, an aqueous solution of K_2_CO_3_ (4.0 eq, 1.0 M) was added to the mixture and the final homogeneous solution was placed in a microwave reactor and exposed to microwave irradiation at 90 °C for 2–3 h (irradiation power reaches its maximum at the beginning of reaction, then it decreases to lower and quite constant values). Once the reaction was completed, detecting by TLC the complete consumption of starting material, after cooling with pressurized air, the mixture was poured into H_2_O and extracted with EtOAc three times. The combined organic layers were dried over Na_2_SO_4_, filtered, and evaporated under reduced pressure to afford the crude products. The latter ones were purified by column chromatography on silica gel, with different mixtures of *n*-hexane/EtOAc to afford the intermediates **1**–**2a**. To synthesize the final products **4**–**11**, the intermediates **1**–**2a** (1.5 eq) were dissolved into DMF (0.4 M), then umbelliferon (1.0 eq) and K_2_CO_3_ (4.0 eq) were added to the mixture, placed in a microwave reactor and exposed to irradiation at 100 °C for 2 h. Once the reaction was completed, the mixture was poured into H_2_O and extracted with EtOAc three times. The combined organic layers were dried over Na_2_SO_4_, filtered, and evaporated under reduced pressure to afford the crude products. These were then subjected to column chromatography on silica gel with different n-hexane/EtOAc mixtures and recrystallization from ethanol to afford compounds **4**–**11**.

***7-(2-(4-chloro-2-isopropyl-5-methylphenoxy)ethoxy)-2H-chromen-2-one (*****4*****).*** Pale yellow solid, m.p. 107–109 °C, 60% yield. ^1^H NMR (300 MHz, Chloroform-*d*) *δ* 1.02–1.35 (s, 6H, 2 × CH_3_ iPro), 2.32 (s, 3H, ArCH_3_), 3.11–3.32 (m, 1H, CH iPro), 4.35 (d, *J* = 18.4 Hz, 4H, OCH_2_CH_2_O), 6.26 (m, 1H, Ar), 6.72 (s, 1H, Ar), 6.87 (m, 2H, Ar), 7.14 (m, 1H, Ar), 7.38 (m, 1H, Ar), 7.64 (m, 1H, Ar). ^13^C NMR (75 MHz, Chloroform-*d*) *δ* 19.9, 22.5, 26.7, 26.7, 29.7, 66.9, 67.1, 101.7, 112.8, 112.9, 113.4, 113.4, 113.5, 114.4, 126.4, 126.8, 128.8, 133.7, 136.8, 143.2, 143.3, 154.2, 155.8, 161.0, 161.0, 161.8. Anal. calcd for C_21_H_21_ClO_4_: C, 67.65; H, 5.68. Found: C, 67.55; H, 5.70.
***7-(2-(4-isopropyl-3-methylphenoxy)ethoxy)-2H-chromen-2-one (*****5*****).*** White solid, m.p. 153–155 °C, 72% yield. ^1^H NMR (300 MHz, Chloroform-*d*) *δ* 1.20 (d, *J* = 6.9 Hz, 6H, 2 × CH_3_ iPro), 2.31 (s, 3H ArCH_3_), 3.07 (p, *J* = 6.9 Hz, 1H CH iPro), 4.34 (m, 4H, OCH_2_CH_2_O), 6.25 (d, *J* = 9.5 Hz, 1H Ar), 6.72–6.81 (m, 2H, Ar), 6.84–6.94 (m, 2H, Ar), 7.16 (d, *J* = 8.2 Hz, 1H, Ar), 7.37 (dd, *J* = 8.3, 0.7 Hz, 1H, Ar), 7.63 (dd, *J* = 9.5, 0.6 Hz, 1H, Ar). ^13^C NMR (75 MHz, Chloroform-*d*) *δ* 19.5, 23.4, 28.6, 28.7, 66.1, 67.2, 101.6, 112.0, 112.8, 113.0, 113.3, 116.6, 125.7, 128.8, 136.5, 139.8, 143.4, 155.8, 156.0, 161.2, 161.9. Anal. calcd for C_21_H_22_O_4_: C, 74.54; H, 6.55. Found: C, 74.61; H, 6.59.
***7-(3-(4-chloro-2-isopropyl-5-methylphenoxy)propoxy)-2H-chromen-2-one (*****6*****).*** White solid, m.p. 103–105 °C, 60% yield. ^1^H NMR (300 MHz, Chloroform-*d*) *δ* 1.16 (d, *J* = 6.9 Hz, 6H, 2 × CH_3_ iPro), 2.27–2.35 (m, 5H, overlapped OCH_2_CH_2_CH_2_O and ArCH_3_), 3.22 (hept, *J* = 6.9 Hz, 1H CH iPro), 4.13 (t, *J* = 5.9 Hz, 2H OCH_2_CH_2_CH_2_O), 4.23 (t, *J* = 6.1 Hz, 2H, OCH_2_CH_2_CH_2_O), 6.25 (d, *J* = 9.5 Hz, 1H, Ar), 6.70 (s, 1H, Ar), 6.81–6.89 (m, 2H, Ar), 7.12 (s, 1H, Ar), 7.36 (d, *J* = 9.2 Hz, 1H, Ar), 7.62 (d, *J* = 9.4 Hz, 1H, Ar). ^13^C NMR (75 MHz, Chloroform-*d*) *δ* 19.9, 22.5, 22.6, 26.7, 29.2, 64.4, 65.1, 101.4, 112.6, 112.8, 113.2, 113.9, 125.8, 126.6, 128.8, 133.6, 136.3, 143.3, 154.3, 155.9, 161.1, 162.0. Anal. calcd for C_22_H_23_ClO_4_: C, 68.30; H, 5.99. Found: C, 68.39; H, 5.95.
***7-(3-(4-isopropyl-3-methylphenoxy)propoxy)-2H-chromen-2-one (*****7*****).*** Pale yellow solid, m.p. 80–82 °C, 78% yield. ^1^H NMR (300 MHz, Chloroform-*d*) *δ* 1.19 (d, *J* = 6.9 Hz, 6H, 2 × CH_3_ iPro), 2.22–2.32 (m, 5H, overlapped OCH_2_CH_2_CH_2_O and ArCH_3_), 3.06 (p, *J* = 6.9 Hz, 1H, CH iPro), 4.13 (t, *J* = 6.0 Hz, 2H, OCH_2_CH_2_CH_2_O), 4.21 (t, *J* = 6.1 Hz, 2H, OCH_2_CH_2_CH_2_O), 6.24 (d, *J* = 9.5 Hz, 1H, Ar), 6.69–6.75 (m, 2H, Ar), 6.81–6.86 (m, 2H, Ar), 7.14 (d, *J* = 8.3 Hz, 1H, Ar), 7.33–7.37 (m, 1H, Ar), 7.62 (dd, *J* = 9.5, 0.6 Hz, 1H, Ar). ^13^C NMR (75 MHz, Chloroform-*d*) *δ* 19.5, 19.5, 23.4, 28.6, 28.6, 29.1, 63.8, 65.1, 101.5, 111.8, 112.5, 112.8, 113.1, 116.4, 125.7, 128.7, 136.4, 139.4, 143.4, 155.9, 156.3, 161.2, 161.2, 162.1. Anal. calcd for C_22_H_24_O_4_: C, 74.98; H, 6.86. Found: C, 74.88; H, 6.89.
***7-(4-(4-isopropyl-3-methylphenoxy)butoxy)-2H-chromen-2-one (*****8*****).*** White solid, m.p. 90–92 °C, 64% yield.^1^H NMR (300 MHz, Chloroform-*d*) *δ* 1.20 (d, *J* = 6.8 Hz, 6H, 2 × CH_3_ iPro), 1.88–2.09 (m, 4H, OCH_2_CH_2_CH_2_CH_2_O), 2.30 (s, 3H, ArCH_3_), 3.06 (hept, *J* = 6.8 Hz, 1H, CH iPro), 4.01 (t, *J* = 5.7 Hz, 2H, OCH_2_CH_2_CH_2_CH_2_O), 4.09 (t, *J* = 5.9 Hz, 2H, OCH_2_CH_2_CH_2_CH_2_O), 6.24 (d, *J* = 9.5 Hz, 1H, Ar), 6.65–6.77 (m, 2H, Ar), 6.77–6.88 (m, 2H, Ar), 7.13 (d, *J* = 8.3 Hz, 1H, Ar), 7.29–7.40 (m, 1H, Ar), 7.62 (d, *J* = 9.5 Hz, 1H, Ar). ^13^C NMR (75 MHz, Chloroform-*d*) *δ* 19.5, 23.4, 25.9, 26.0, 28.6, 67.1, 68.2, 101.4, 111.7, 112.5, 112.9, 113.0, 116.3, 125.6, 128.7, 136.4, 139.2, 143.4, 155.9, 156.5, 161.3, 162.3. Anal. calcd for C_23_H_26_O_4_: C, 75.38; H, 7.15. Found: C, 75.45; H, 7.19.
***7-((5-(4-isopropyl-3-methylphenoxy)pentyl)oxy)-2H-chromen-2-one (*****9*****).*** Yellow oil, 59% yield. ^1^H NMR (300 MHz, Chloroform-*d*) *δ* 1.20 (d, *J* = 6.9 Hz, 6H, 2 × CH_3_ iPro), 1.67 (q, *J* = 8.1, 7.6 Hz, 2H, OCH_2_CH_2_CH_2_CH_2_CH_2_O), 1.87 (m, 4H, OCH_2_CH_2_CH_2_CH_2_CH_2_O), 2.31 (s, 3H, ArCH_3_), 3.07 (p, *J* = 6.9 Hz, 1H, CH iPro), 4.01 (dt, *J* = 22.5, 6.3 Hz, 4H, OCH_2_CH_2_CH_2_CH_2_O), 6.24 (dd, *J* = 9.5, 1.3 Hz, 1H, Ar), 6.71 (m, 2H, Ar), 6.83 (m, 2H, Ar), 7.13 (d, *J* = 8.2 Hz, 1H, Ar), 7.35 (d, *J* = 8.4 Hz, 1H, Ar), 7.62 (d, *J* = 9.4 Hz, 1H, Ar). ^13^C NMR (75 MHz, Chloroform-*d*) *δ* 19.5, 22.7, 23.4, 28.6, 28.8, 29.1, 29.7, 67.5, 68.4, 101.3, 111.7, 112.4, 113.0, 116.4, 125.6, 128.7, 136.3, 139.1, 143.4, 155.9, 156.6, 161.2, 161.3, 162.3. Anal. calcd for C_24_H_28_O_4_: C, 75.76; H, 7.42. Found: C, 75.82; H, 7.48.
***7-((6-(4-chloro-2-isopropyl-5-methylphenoxy)hexyl)oxy)-2H-chromen-2-one (*****10*****).*** Pale yellow solid, m.p. 104–105 °C, 50% yield. ^1^H NMR (300 MHz, Chloroform-*d*) *δ* 1.17 (d, *J* = 6.9 Hz, 6H, 2 × CH_3_ iPro), 1.54–1.59 (m, 4H, OCH_2_CH_2_CH_2_CH_2_CH_2_CH_2_O), 1.76–1.91 (m, 4H, OCH_2_CH_2_CH_2_CH_2_CH_2_CH_2_O), 2.31 (s, 3H, ArCH_3_), 3.23 (hept, *J* = 6.9 Hz, 1H, CH iPro), 3.94 (t, *J* = 6.2 Hz, 2H, OCH_2_CH_2_CH_2_CH_2_CH_2_CH_2_O), 4.03 (t, *J* = 6.5 Hz, 2H, OCH_2_CH_2_CH_2_CH_2_CH_2_CH_2_O), 6.25 (d, *J* = 9.5 Hz, 1H, Ar), 6.67 (s, 1H, Ar), 6.76–6.87 (m, 2H, Ar), 7.11 (s, 1H, Ar), 7.36 (d, *J* = 8.4 Hz, 1H, Ar), 7.63 (d, *J* = 9.5 Hz, 1H, Ar). ^13^C NMR (75 MHz, Chloroform-*d*) *δ* 56.6, 111.4, 112.3, 123.2, 128.8, 130.2, 131.3, 149.3, 150.0, 189.9. Anal. calcd for C_25_H_29_ClO_4_: C, 70.00; H, 6.81. Found: C, 70.10; H, 6.87.
***7-((6-(4-isopropyl-3-methylphenoxy)hexyl)oxy)-2H-chromen-2-one (*****11*****).*** Yellow oil, 59% yield. ^1^H NMR (300 MHz, Chloroform-*d*) *δ* 1.19 (d, *J* = 6.9 Hz, 6H, 2 × CH_3_ iPro), 1.54 (m, 4H, OCH_2_CH_2_CH_2_CH_2_CH_2_CH_2_O), 1.81 (m, 4H, OCH_2_CH_2_CH_2_CH_2_CH_2_CH_2_O), 2.30 (s, 3H, ArCH_3_), 3.06 (p, *J* = 6.9 Hz, 1H, CH iPro), 3.95 (t, *J* = 6.5 Hz, 2H, OCH_2_CH_2_CH_2_CH_2_CH_2_CH_2_O), 4.02 (t, *J* = 6.5 Hz, 2H, OCH_2_CH_2_CH_2_CH_2_CH_2_CH_2_O), 6.23 (dd, *J* = 9.5, 0.8 Hz, 1H, Ar), 6.67–6.74 (m, 1H, Ar m, 2H, Ar), 6.79– 6.84 (m, 2H, Ar), 7.12 (d, *J* = 8.3 Hz, 1H, Ar), 7.34 (d, *J* = 8.7 Hz, 1H, Ar), 7.62 (dd, *J* = 9.5, 0.8 Hz, 1H, Ar). ^13^C NMR (75 MHz, Chloroform-*d*) *δ* 19.5, 23.4, 25.8, 25.9, 28.6, 28.9, 29.3, 67.6, 68.5, 101.3, 111.7, 112.4, 112.9, 113.0, 116.4, 125.6, 128.7, 136.3, 139.0, 143.4, 155.9, 156.7, 161.3, 162.4. Anal. calcd for C_25_H_30_O_4_: C, 76.11; H, 7.67. Found: C, 76.17; H, 7.70.

##### General Procedure for the Synthesis of Compounds 13–21 (Series B)

To obtain azido-derivatives, the proper **1**–**2a** intermediate (1.0 eq) was dissolved in DMF (1.0 M), then NaN_3_ (1.2 eq) was added to the mixture, and it was allowed to stir for 2 h at room temperature. The reaction mixture was quenched with water, extracted three times with EtOAc, the organic layers were recollected and dried over Na_2_SO_4_, filtered, and evaporated under reduced pressure to reach compounds **1**–**2b** with no further purification. Successively, umbelliferon (1.0 eq), propargyl bromide (1.5 eq) and K_2_CO_3_ (4.0 eq) were added to ACN or cyrene (0.4 M) and the mixture was allowed to reflux for 12 h. Once the reaction was completed, the mixture was poured into H_2_O and extracted with EtOAc three times. The combined organic layers were dried over Na_2_SO_4_, filtered, and evaporated under reduced pressure to afford the crude product **12**, that was purified by column chromatography on silica gel with 8:2 *n*-hexane/EtOAc mixture [79]. Finally, azido-derivatives **1**–**2b** (1.2 eq), propargyl-umbelliferon **12** (1.0 eq), CuSO_4_ × 5 H_2_O (0.01 eq) and sodium ascorbate (0.1 eq) were dissolved in a mixture of 1:1 H_2_O (0.25 M) and *tert*-butanol (0.25 M). The reaction stirred overnight at room temperature, after completion the mixture reaction was poured into H_2_O and extracted with EtOAc three times. The organic layers were dried over Na_2_SO_4_, filtered, and evaporated under reduced pressure to afford the crude products. These were purified by column chromatography on silica gel with different *n*-hexane/EtOAc mixtures and recrystallization from ethanol (when solid) to afford compounds **13**–**21**.

***7-((1-(2-(4-chloro-2-isopropyl-5-methylphenoxy)ethyl)-1H-1,2,3-triazol-4-yl)methoxy)-2H-chromen-2-one (*****13*****).*** White solid, m.p. 133–135 °C, 69% yield. ^1^H NMR (300 MHz, DMSO-*d*_6_) *δ* 0.92 (d, *J* = 6.9 Hz, 6H, 2 × CH_3_ iPro), 2.23 (s, 3H, ArCH_3_), 2.87–2.97 (m, 1H, CH iPro), 4.37 (t, *J* = 5.0 Hz, 2H, OCH_2_CH_2_N), 4.79 (t, *J* = 5.0 Hz, 2H, OCH_2_CH_2_N), 5.24 (s, 2H, OCH_2_), 6.27 (d, *J* = 9.5 Hz, 1H, Ar), 6.89–7.01 (m, 2H, Ar), 7.02–7.15 (m, 2H, Ar), 7.60 (d, *J* = 8.7 Hz, 1H), 7.93–8.00 (m, 1H, Ar), 8.30 (s, 1H, triazole). Anal. calcd for C_24_H_24_ClN_3_O_4_: C, 63.51; H, 5.33; N, 9.26. Found: C, 63.59; H, 5.38; N, 9.30.
***7-((1-(3-(4-chloro-2-isopropyl-5-methylphenoxy)propyl)-1H-1,2,3-triazol-4-yl)methoxy)-2H-chromen-2-one (*****14*****).*** White solid, m.p. 104–106 °C, 58% yield. ^1^H NMR (300 MHz, DMSO-*d*_6_) *δ* 1.12 (d, *J* = 6.9 Hz, 6H, 2 × CH_3_ iPro), 2.23 (s, 3H, ArCH_3_), 2.30 (q, *J* = 6.4 Hz, 2H, OCH_2_CH_2_CH_2_N), 3.09–3.21 (m, 1H, CH iPro), 3.94 (t, *J* = 5.9 Hz, 2H, OCH_2_CH_2_CH_2_N), 4.54 (t, *J* = 6.9 Hz, 2H, OCH_2_CH_2_CH_2_N), 5.24 (s, 2H, OCH_2_), 6.28 (d, *J* = 9.5 Hz, 1H, Ar), 6.86 (d, *J* = 0.7 Hz, 1H, Ar), 6.98 (dd, *J* = 8.6, 2.4 Hz, 1H, Ar), 7.08–7.16 (m, 2H, Ar), 7.61 (d, *J* = 8.6 Hz, 1H, Ar), 7.97 (dd, *J* = 9.6, 0.6 Hz, 1H, Ar), 8.31 (s, 1H, triazole). ^13^C NMR (75 MHz, DMSO-*d*_6_) *δ* 19.8, 22.8, 26.5, 29.8, 47.2, 61.9, 65.2, 101.9, 113.0, 113.5, 114.8, 125.0, 125.3, 126.4, 130.0, 133.9, 136.4, 142.6, 144.9, 154.4. Anal. calcd for C_25_H_26_ClN_3_O_4_: C, 64.17; H, 5.60; N, 8.98. Found: C, 64.10; H, 5.64; N, 8.94.
***7-((1-(3-(4-isopropyl-3-methylphenoxy)propyl)-1H-1,2,3-triazol-4-yl)methoxy)-2H-chromen-2-one (*****15*****).*** White solid, m.p. 98–100 °C, 62% yield. ^1^H NMR (300 MHz, Chloroform-*d*) *δ* 1.19 (d, *J* = 6.8 Hz, 6H, 2 × CH_3_ iPro), 2.30 (s, 3H, ArCH_3_), 2.33–2.45 (m, 2H, OCH_2_CH_2_CH_2_N), 3.06 (hept, *J* = 6.9 Hz, 1H, CH iPro), 3.93 (t, *J* = 5.9 Hz, 2H, OCH_2_CH_2_CH_2_N), 4.60 (t, *J* = 6.9 Hz, 2H, OCH_2_CH_2_CH_2_N), 5.24 (s, 2H, OCH_2_), 6.26 (d, *J* = 9.5 Hz, 1H, Ar), 6.65–6.71 (m, 2H, Ar), 6.89–6.94 (m, 2H, Ar), 7.14 (d, *J* = 8.0 Hz, 1H, Ar), 7.35–7.40 (m, 1H, Ar), 7.61–7.67 (m, 2H, overlapped Ar and triazole). ^13^C NMR (75 MHz, Chloroform-*d*) *δ* 19.5, 21.5, 23.4, 28.6, 29.9, 47.3, 62.3, 63.7, 102.1, 111.7, 112.7, 113.0, 113.5, 116.3, 123.6, 125.7, 128.9, 136.5, 139.7, 142.8, 143.3, 155.7, 155.9, 161.1, 161.3. Anal. calcd for C_25_H_27_N_3_O_4_: C, 69.27; H, 6.28; N, 9.69. Found: C, 69.37; H, 6.30; N, 9.65.
***7-((1-(4-(4-chloro-2-isopropyl-5-methylphenoxy)butyl)-1H-1,2,3-triazol-4-yl)methoxy)-2H-chromen-2-one (*****16*****)***. White solid, m.p. 102–104 °C, 72% yield. ^1^H NMR (300 MHz, DMSO-*d*_6_) *δ* 1.09 (d, *J* = 6.9 Hz, 6H, 2 × CH_3_ iPro), 1.68 (p, *J* = 6.2 Hz, 2H, OCH_2_CH_2_CH_2_CH_2_N), 1.92–2.03 (m, 2H, OCH_2_CH_2_CH_2_CH_2_N), 2.24 (s, 4H, ArCH_3_), 3.11 (q, *J* = 6.9 Hz, 1H, CH iPro), 3.96 (t, *J* = 6.2 Hz, 2H, OCH_2_CH_2_CH_2_CH_2_N), 4.45 (t, *J* = 7.0 Hz, 2H, OCH_2_CH_2_CH_2_CH_2_N), 5.24 (s, 2H, OCH_2_), 6.28 (d, *J* = 9.5 Hz, 1H, Ar), 6.89 (s, 1H, Ar), 6.99 (dd, *J* = 8.6, 2.4 Hz, 1H, Ar), 7.07–7.16 (m, 2H, Ar), 7.62 (d, *J* = 8.7 Hz, 1H, Ar), 7.98 (dd, *J* = 9.6, 0.5 Hz, 1H, Ar), 8.29 (s, 1H, triazole). ^13^C NMR (75 MHz, Chloroform-*d +* DMSO-*d*_6_ due to solubility issues) *δ* 24.7, 27.3, 31.0, 31.2, 31.8, 54.7, 66.9, 71.9, 106.6, 117.7, 117.8, 118.7, 128.5, 130.0, 131.1, 134.0, 138.2, 140.9, 147.4, 148.6, 159.1, 160.3, 165.7, 166.1, 229.0. Anal. calcd for C_26_H_28_ClN_3_O_4_: C, 64.79; H, 5.86; N, 8.72. Found: C, 64.86; H, 5.90; N, 8.75.
***7-((1-(4-(4-isopropyl-3-methylphenoxy)butyl)-1H-1,2,3-triazol-4-yl)methoxy)-2H-chromen-2-one (*****17*****).*** White solid, m.p. 108–109 °C, 76% yield. ^1^H NMR (300 MHz, Chloroform-*d*) *δ* 1.19 (d, *J* = 6.8 Hz, 6H, 2 × CH_3_ iPro), 1.80 (m, 2H, OCH_2_CH_2_CH_2_CH_2_N), 2.13 (, 2H, OCH_2_CH_2_CH_2_CH_2_N), 2.30 (s, 3H, ArCH_3_), 3.06 (hept, *J* = 6.9 Hz, 1H, CH iPro), 3.96 (t, *J* = 5.9 Hz, 2H, OCH_2_CH_2_CH_2_N), 4.47 (t, *J* = 7.2 Hz, 2H, OCH_2_CH_2_CH_2_N), 5.25 (s, 2H, OCH_2_), 6.25 (d, *J* = 9.5 Hz, 1H, Ar), 6.62–6.73 (m, 2H, Ar), 6.88–6.98 (m, 2H, Ar), 7.13 (d, *J* = 8.2 Hz, 1H, Ar), 7.31–7.42 (m, 1H, Ar), 7.62 (d, *J* = 9.5 Hz, 1H, Ar), 7.67 (s, 1H, triazole). ^13^C NMR (75 MHz, Chloroform-*d*) *δ* 19.5, 23.4, 26.2, 27.3, 28.6, 50.2, 62.4, 66.7, 102.2, 111.7, 112.7, 113.0, 113.5, 116.3, 122.9, 125.7, 128.9, 136.4, 139.4, 143.0, 143.3, 155.7, 156.3, 161.0, 161.3. Anal. calcd for C_26_H_29_N_3_O_4_: C, 69.78; H, 6.53; N, 9.39. Found: C, 69.70; H, 6.50; N, 9.34.
***7-((1-(5-(4-chloro-2-isopropyl-5-methylphenoxy)pentyl)-1H-1,2,3-triazol-4-yl)methoxy)-2H-chromen-2-one (*****18*****)***. White solid, m.p. 106–108 °C, 83% yield. ^1^H NMR (300 MHz, DMSO-*d*_6_) *δ* 1.09 (d, *J* = 6.9 Hz, 6H, 2 × CH_3_ iPro), 1.31–1.47 (m, 2H, OCH_2_CH_2_CH_2_CH_2_CH_2_N), 1.75 (p, *J* = 6.8 Hz, 2H, OCH_2_CH_2_CH_2_CH_2_CH_2_N), 1.90 (p, *J* = 7.1 Hz, 2H, OCH_2_CH_2_CH_2_CH_2_CH_2_N), 2.25 (s, 3H, ArCH_3_), 3.11 (p, *J* = 6.9 Hz, 1H, CH iPro), 3.92 (t, *J* = 6.2 Hz, 2H, OCH_2_CH_2_CH_2_CH_2_CH_2_N), 4.40 (t, *J* = 7.0 Hz, 2H, OCH_2_CH_2_CH_2_CH_2_CH_2_N), 5.25 (s, 2H, OCH_2_), 6.29 (d, *J* = 9.5 Hz, 1H, Ar), 6.89 (s, 1H, Ar), 7.00 (dd, *J* = 8.6, 2.5 Hz, 1H, Ar), 7.07–7.18 (m, 2H, Ar), 7.63 (d, *J* = 8.6 Hz, 1H, Ar), 7.99 (d, *J* = 9.5 Hz, 1H, Ar), 8.28 (s, 1H, Ar).^13^C NMR (75 MHz, DMSO-*d*_6_) *δ* 20.0, 22.5, 23.3, 26.7, 28.7, 30.0, 50.4, 62.4, 67.6, 102.1, 112.7, 113.0, 113.5, 113.7, 122.7, 125.5, 126.5, 126.6, 128.9, 133.6, 136.3, 143.0, 143.3, 154.5, 155.7, 161.0, 161.3. Anal. calcd for C_27_H_30_ClN_3_O_4_: C, 65.38; H, 6.10; N, 8.47. Found: C, 65.45; H, 6.14; N, 8.42.
***7-((1-(5-(4-isopropyl-3-methylphenoxy)pentyl)-1H-1,2,3-triazol-4-yl)methoxy)-2H-chromen-2-one (*****19*****)***. White solid, m.p. 109–110 °C, 57% yield. ^1^H NMR (300 MHz, Chloroform-*d*) *δ* 1.19 (d, *J* = 6.8 Hz, 6H, 2 × CH_3_ iPro), 1.46–1.60 (m, 2H, OCH_2_CH_2_CH_2_CH_2_CH_2_N), 1.73–1.88 (m, 2H, OCH_2_CH_2_CH_2_CH_2_CH_2_N), 2.01 (dt, *J* = 15.0, 7.5 Hz, 2H, OCH_2_CH_2_CH_2_CH_2_CH_2_N), 2.30 (s, 3H, ArCH_3_), 3.06 (hept, *J* = 6.9 Hz, 1H, CH iPro), 3.91 (t, *J* = 6.1 Hz, 2H, OCH_2_CH_2_CH_2_CH_2_CH_2_N), 4.40 (t, *J* = 7.2 Hz, 2H, OCH_2_CH_2_CH_2_CH_2_CH_2_N), 5.26 (d, *J* = 0.6 Hz, 2H, OCH_2_), 6.26 (d, *J* = 9.5 Hz, 1H, Ar), 6.62–6.73 (m, 2H, Ar), 6.89–6.98 (m, 2H, Ar), 7.12 (d, *J* = 8.2 Hz, 1H, Ar), 7.33–7.42 (m, 1H, Ar), 7.62 (d, *J* = 9.2 Hz, 2H, Ar). ^13^C NMR (75 MHz, Chloroform-*d*) *δ* 19.5, 23.3, 23.4, 28.6, 28.7, 30.0, 50.4, 62.4, 67.1, 102.2, 111.7, 112.7, 113.0, 113.5, 116.3, 122.8, 125.6, 128.9, 136.3, 139.2, 142.9, 143.3, 155.7, 156.5, 161.0, 161.3. Anal. calcd for C_27_H_31_N_3_O_4_: C, 70.26; H, 6.77; N, 9.10. Found: C, 70.32; H, 6.80; N, 9.14.
***7-((1-(6-(4-chloro-2-isopropyl-5-methylphenoxy)hexyl)-1H-1,2,3-triazol-4-yl)methoxy)-2H-chromen-2-one (*****20*****).*** White solid, m.p. 107–109 °C, 60% yield. ^1^H NMR (300 MHz, Chloroform-*d*) *δ* 1.17 (d, *J* = 6.9 Hz, 6H, 2 × CH_3_ iPro), 1.33–1.48 (m, 2H, OCH_2_CH_2_CH_2_CH_2_CH_2_CH_2_N), 1.54 (m, 2H, OCH_2_CH_2_CH_2_CH_2_CH_2_CH_2_N), 1.71–1.86 (m, 2H, OCH_2_CH_2_CH_2_CH_2_CH_2_CH_2_N), 1.97 (m, 2H, OCH_2_CH_2_CH_2_CH_2_CH_2_CH_2_N), 2.30 (s, 3H, ArCH_3_), 3.21 (hept, *J* = 6.9 Hz, 1H, CH iPro), 3.90 (t, *J* = 6.2 Hz, 2H, OCH_2_CH_2_CH_2_CH_2_CH_2_CH_2_N), 4.39 (t, *J* = 7.2 Hz, 2H, OCH_2_CH_2_CH_2_CH_2_CH_2_CH_2_N), 5.25 (s, 2H, OCH_2_), 6.26 (d, *J* = 9.5 Hz, 1H, Ar), 6.64 (s, 1H, Ar), 6.88–6.98 (m, 2H, Ar), 7.11 (s, 1H, Ar), 7.33–7.43 (m, 1H, Ar), 7.58–7.67 (m, 2H, overlapped Ar and triazole). ^1^H NMR (75 MHz, Chloroform-*d*) *δ* 14.1, 20.0, 22.6, 25.6, 26.2, 26.7, 29.1, 30.2, 50.4, 62.4, 67.8, 102.1, 112.7, 113.0, 113.5, 113.7, 122.8, 125.4, 126.5, 128.9, 133.5, 136.3, 142.9, 143.3, 154.6, 155.7, 161.1, 161.3. Anal. calcd for C_28_H_32_ClN_3_O_4_: C, 65.94; H, 6.32; N, 8.24. Found: C, 65.99; H, 6.30; N, 8.28.
***7-((1-(6-(4-isopropyl-3-methylphenoxy)hexyl)-1H-1,2,3-triazol-4-yl)methoxy)-2H-chromen-2-one (*****21*****).*** White solid, m.p. 101–102 °C, 64% yield ^1^H NMR (300 MHz, Chloroform-*d*) *δ* 1.17 (d, *J* = 7.2 Hz, 6H, 2 × CH_3_ iPro), 1.38–1.48 (m, 4H, OCH_2_CH_2_CH_2_CH_2_CH_2_CH_2_N), 1.62–1.73 (m, 2H, OCH_2_CH_2_CH_2_CH_2_CH_2_CH_2_N), 1.89–1.97 (m, 2H, OCH_2_CH_2_CH_2_CH_2_CH_2_CH_2_N), 2.29 (s, 3H, ArCH_3_), 2.98–3.07 (m, 1H, CH iPro), 3.83–3.91 (m, 2H, OCH_2_CH_2_CH_2_CH_2_CH_2_N), 4.30–4.39 (m, 2H, OCH_2_CH_2_CH_2_CH_2_CH_2_N), 5.24 (s, 2H, OCH_2_), 6.25 (d, *J* = 9.9 Hz, 1H, Ar), 6.62–6.69 (m, 2H, Ar), 6.87–6.93 (m, 2H, Ar), 7.06–7.13 (m, 1H, Ar), 7.03–7.38 (m, 1H, Ar), 7.56–7.63 (m, 2H, 1 from Ar and 1 from Triazole). ^13^C NMR (75 MHz, Chloroform-*d*) *δ* 19.5, 23.4, 25.5, 26.2, 28.6, 29.1, 30.2, 50.4, 62.4, 67.3, 102.1, 111.7, 112.7, 113.0, 113.5, 116.3, 122.8, 125.6, 128.9, 136.3, 139.1, 142.9, 143.3, 155.7, 156.6, 161.0, 161.3. Anal. calcd for C_28_H_33_N_3_O_4_: C, 70.71; H, 6.99; N, 8.84. Found: C, 70.77; H, 7.03; N, 8.88.

##### General Procedure for the Synthesis of Compounds **23**–**30** (Series C)

To obtain **DPA** derivatives, compounds **1**–**2a** (2.0 eq) were dissolved in ACN (0.4 M), then **DPA** (**22**, 1.0 eq) was added to the mixture, and lastly, an aqueous solution (5.5 M) of K_2_CO_3_ (3.0 eq) was added and the solution was allowed to stir overnight at 100 °C. The completion of the reaction was monitored by TLC, employing ninhydrin staining, which showed a positive result exclusively for **DPA**. Once reaction was completed, the crude was extracted with EtOAc for three times. The organic layers were dried over Na_2_SO_4_, filtered, and evaporated under reduced pressure to afford the crude products. Finally, the crude product was purified using 5% of MeOH in DCM, to obtain compounds **23**–**30**.

***2-(4-isopropyl-3-methylphenoxy)-N,N-bis(pyridin-2-ylmethyl)ethan-1-amine (*****23*****).*** Green oil, 57% yield. ^1^H NMR (300 MHz, Chloroform-*d*) *δ* 1.18 (d, *J* = 6.9 Hz, 6H, 2 × CH_3_ iPro), 2.28 (s, 3H, ArCH_3_), 3.03 (m, 3H, overlapped OCH_2_CH_2_N and CH iPro), 3.96 (s, 4H, NCH_2_-pyr), 4.07 (t, *J* = 5.8 Hz, 2H, OCH_2_CH_2_N), 6.62–6.70 (m, 2H, Ar), 7.07–7.17 (m, 3H, Ar), 7.57 (d, *J* = 7.8 Hz, 2H, Ar), 7.60–7.68 (m, 2H, Ar), 8.52 (d, *J* = 4.9 Hz, 2H, Ar). ^13^C NMR (75 MHz, Chloroform-*d*) *δ* 19.5, 23.4, 28.6, 53.1, 60.9, 66.0, 111.8, 116.3, 122.0, 123.0, 125.5, 136.3, 136.5, 139.1, 149.0, 156.3, 159.6. Anal. calcd for C_24_H_29_N_3_O: C, 76.76; H, 7.78; N, 11.19. Found: C, 76.68; H, 7.74; N, 11.15.
***3-(4-isopropyl-3-methylphenoxy)-N,N-bis(pyridin-2-ylmethyl)propan-1-amine (*****24*****).*** Brown oil, 55% yield. ^1^H NMR (300 MHz, Methanol-*d*_4_) *δ* 1.18 (dt, *J* = 6.7, 1.4 Hz, 6H, 2 × CH_3_ iPro), 1.95 (p, *J* = 6.3 Hz, 2H, OCH_2_CH_2_CH_2_N), 2.27 (s, 3H, ArCH_3_), 2.74 (t, *J* = 6.7 Hz, 2H, OCH_2_CH_2_CH_2_N), 3.08 (m, 1H, CH iPro), 3.84 (s, 4H, NCH_2_-pyr), 3.95 (t, *J* = 5.8 Hz, 2H, OCH_2_CH_2_CH_2_N), 6.48–6.66 (m, 2H, Ar), 7.08 (d, *J* = 8.4 Hz, 1H, Ar), 7.24 (m, 2H, Ar), 7.57 (dd, *J* = 7.9, 1.2 Hz, 2H, Ar), 7.69 (td, *J* = 7.6, 1.7 Hz, 2H, Ar), 8.33–8.47 (m, 2H, Ar). ^13^C NMR (75 MHz, Methanol-*d*_4_) *δ* 19.6, 23.9, 27.8, 29.7, 52.1, 61.1, 66.3, 112.9, 117.4, 123.8, 124.9, 126.4, 137.0, 138.7, 140.1, 149.4, 157.9, 160.2. Anal. calcd for C_25_H_31_N_3_O: C, 77.08; H, 8.02; N, 10.79. Found: C, 77.12; H, 8.05; N, 10.83.
***4-(4-chloro-2-isopropyl-5-methylphenoxy)-N,N-bis(pyridin-2-ylmethyl)butan-1-amine (*****25*****)***. Brown oil, 50% yield. ^1^H NMR (300 MHz, Chloroform-*d*) *δ* 1.13 (d, *J* = 6.9 Hz, 6H, 2 × CH_3_ iPro), 1.69–1.80 (m, 4H, OCH_2_CH_2_CH_2_CH_2_N), 2.29 (s, 3H, ArCH_3_), 2.62 (t, *J* = 6.7 Hz, 2H, OCH_2_CH_2_CH_2_CH_2_N), 3.18 (hept, *J* = 6.9 Hz, 1H, CH iPro), 3.83 (m, 6H, overlapped OCH_2_CH_2_CH_2_CH_2_N and NCH_2_-pyr), 6.59 (s, 1H, Ar), 7.06–7.19 (m, 3H, Ar), 7.52 (dt, *J* = 7.9, 1.1 Hz, 2H, Ar), 7.63 (td, *J* = 7.6, 1.8 Hz, 2H, Ar), 8.51 (m, 2H, Ar). ^13^C NMR (75 MHz Chloroform-*d*) *δ* 20.0, 22.5, 23.8, 26.6, 27.1, 53.9, 60.4, 67.8, 113.7, 121.9, 122.9, 125.2, 126.4, 133.4, 136.3, 136.4, 149.0, 154.6, 159.8. Anal. calcd for C_26_H_32_ClN_3_O: C, 71.30; H, 7.36; N, 9.59. Found: C, 71.38; H, 7.39; N, 9.55.
***4-(4-isopropyl-3-methylphenoxy)-N,N-bis(pyridin-2-ylmethyl)butan-1-amine (*****26*****).*** Brown oil, 70% yield. ^1^H NMR (300 MHz, Methanol-*d*_4_) *δ* 1.18 (dd, *J* = 6.9, 2.7 Hz, 6H, 2 × CH_3_ iPro), 1.71 (m, 4H, OCH_2_CH_2_CH_2_CH_2_N), 2.26 (d, *J* = 2.3 Hz, 3H, ArCH_3_), 2.60 (m, 2H, OCH_2_CH_2_CH_2_CH_2_N), 3.08 (m, 1H, CH iPro), 3.81 (m, 6H, overlapped OCH_2_CH_2_CH_2_CH_2_N and NCH_2_-pyr), 6.61 (m, 2H, Ar), 7.08 (dd, *J* = 8.4, 2.4 Hz, 1H, Ar), 7.27 (m, 2H, Ar), 7.62 (d, *J* = 7.9, 2H, Ar), 7.77 (tt, *J* = 7.7, 2.8 Hz, 2H, Ar), 8.42 (m, 2H, Ar). ^13^C NMR (75 MHz, Methanol-*d*_4_) *δ* 19.6, 23.9, 24.5, 28.0, 29.7, 55.1, 61.1, 68.4, 113.0, 117.3, 123.8, 124.9, 126.5, 137.1, 138.7, 140.0, 149.4, 158.0, 160.6. Anal. calcd for C_26_H_33_N_3_O: C, 77.38; H, 8.24; N, 10.41. Found: C, 77.44; H, 8.28; N, 10.45.
***5-(4-chloro-2-isopropyl-5-methylphenoxy)-N,N-bis(pyridin-2-ylmethyl)pentan-1-amine (*****27*****)***. Brown oil, 48% yield. ^1^H NMR (300 MHz, Chloroform-*d*) *δ* 1.15 (d, *J* = 6.9 Hz, 6H, 2 × CH_3_ iPro), 1.41–1.52 (m, 2H, OCH_2_CH_2_CH_2_CH_2_CH_2_N), 1.58 (dt, *J* = 14.4, 7.2 Hz, 2H, OCH_2_CH_2_CH_2_CH_2_CH_2_N), 1.65–1.76 (m, 2H, OCH_2_CH_2_CH_2_CH_2_CH_2_N), 2.30 (s, 3H, ArCH_3_), 2.48–2.62 (m, 2H, OCH_2_CH_2_CH_2_CH_2_CH_2_N), 3.20 (p, *J* = 6.9 Hz, 1H, CH iPro), 3.81 (s, 4H, NCH_2_-Pyridine), 3.86 (t, *J* = 6.3 Hz, 2H, Ar), 6.63 (s, 1H, Ar), 7.08–7.17 (m, 2H, Ar), 7.53 (d, *J* = 7.9 Hz, 2H, Ar), 7.63 (td, *J* = 7.6, 1.8 Hz, 2H, Ar), 8.47–8.55 (m, 2H, Ar). ^13^C NMR (75 MHz, Chloroform-*d*) *δ* 20.2, 22.7, 24.0, 26.8, 26.9, 29.3, 54.4, 60.6, 68.1, 113.9, 122.0, 122.9, 125.4, 126.6, 133.6, 136.4, 136.5, 149.1, 154.8, 160.1. Anal. calcd for C_27_H_34_ClN_3_O: C, 71.74; H, 7.58; N, 9.30. Found: C, 71.79; H, 7.60; N, 9.35.
***5-(4-isopropyl-3-methylphenoxy)-N,N-bis(pyridin-2-ylmethyl)pentan-1-amine (*****28*****).*** Brown oil, 60% yield. ^1^H NMR (300 MHz, Methanol-*d*_4_) *δ* 1.17 (d, *J* = 6.9 Hz, 6H, 2 × CH_3_ iPro), 1.35–1.50 (m, 2H, OCH_2_CH_2_CH_2_CH_2_CH_2_N), 1.54–1.69 (m, 4H, OCH_2_CH_2_CH_2_CH_2_CH_2_N), 2.26 (s, 3H, ArCH_3_), 2.51–2.62 (m, 2H, OCH_2_CH_2_CH_2_CH_2_CH_2_N), 3.07 (hept, *J* = 6.9 Hz, 1H, CH iPro), 3.80 (s, 4H, NCH_2_-pyr), 3.86 (t, *J* = 6.3 Hz, 2H, OCH_2_CH_2_CH_2_CH_2_CH_2_N),), 6.59–6.70 (m, 2H, Ar), 7.09 (d, *J* = 8.3 Hz, 1H, Ar), 7.26 (ddd, *J* = 7.5, 5.0, 1.3 Hz, 2H, Ar), 7.62 (dt, *J* = 7.9, 1.1 Hz, 2H. Ar), 7.76 (td, *J* = 7.7, 1.8 Hz, 2H, Ar), 8.41–8.43 (m, 2H, Ar). ^13^C NMR (75 MHz, Chloroform-*d*) *δ* 19.6, 23.6, 23.9, 28.8, 29.3, 29.9, 54.4, 60.5, 67.7, 111.9, 116.5, 122.2, 123.1, 125.7, 136.4, 139.1, 149.2, 156.9. Anal. calcd for C_27_H_35_N_3_O: C, 77.66; H, 8.45; N, 10.06. Found: C, 77.75; H, 8.48; N, 10.10.
***6-(4-chloro-2-isopropyl-5-methylphenoxy)-N,N-bis(pyridin-2-ylmethyl)hexan-1-amine (*****29*****).*** Brown oil, 68% yield. ^1^H NMR (300 MHz, Methanol-*d*_4_) *δ* 1.14 (d, *J* = 6.9 Hz, 6H, 2 × CH_3_ iPro), 1.25–1.48 (m, 4H, OCH_2_CH_2_CH_2_CH_2_CH_2_CH_2_N), 1.47–1.65 (m, 2H, OCH_2_CH_2_CH_2_CH_2_CH_2_CH_2_N), 1.72 (dt, *J* = 7.9, 6.2 Hz, 2H, OCH_2_CH_2_CH_2_CH_2_CH_2_CH_2_N), 2.29 (s, 3H, ArCH_3_), 2.51–2.59 (m, 2H, OCH_2_CH_2_CH_2_CH_2_CH_2_CH_2_N), 3.19 (p, *J* = 6.9 Hz, 1H, CH iPro), 3.79 (s, 4H, NCH_2_-pyr), 3.90 (t, *J* = 6.2 Hz, 2H, OCH_2_CH_2_CH_2_CH_2_CH_2_CH_2_N), 6.76 (s, 1H, Ar), 7.06 (s, 1, Ar H), 7.26 (ddd, *J* = 7.5, 5.0, 1.3 Hz, 2H, Ar), 7.61 (d, *J* = 7.9 Hz, 2H, Ar), 7.77 (td, *J* = 7.7, 1.8 Hz, 2H, Ar), 8.42 (dd, *J* = 5.0, 1.8 Hz, 2H, Ar). ^13^C NMR (75 MHz, Chloroform-*d*) *δ* 17.9, 21.2, 21.9, 22.2, 63.3, 109.0, 117.2, 118.1, 121.7, 128.7, 131.6, 144.3. Anal. calcd for C_28_H_36_ClN_3_O: C, 72.16; H, 7.79; N, 9.02. Found: C, 72.25; H, 7.82; N, 9.00.
***6-(4-isopropyl-3-methylphenoxy)-N,N-bis(pyridin-2-ylmethyl)hexan-1-amine (*****30*****).*** Brown oil, 55% yield. ^1^H NMR (300 MHz, Methanol-*d*_4_) *δ* 1.18 (d, *J* = 6.9 Hz, 6H, 2 × CH_3_ iPro), 1.26–1.43 (m, 4H, OCH_2_CH_2_CH_2_CH_2_CH_2_CH_2_N), 1.56 (p, *J* = 7.1 Hz, 2H, OCH_2_CH_2_CH_2_CH_2_CH_2_CH_2_N), 1.62–1.73 (m, 2H, OCH_2_CH_2_CH_2_CH_2_CH_2_CH_2_N), 2.27 (s, 3H, ArCH_3_), 2.56 (t, *J* = 7.4 Hz, 2H, OCH_2_CH_2_CH_2_CH_2_CH_2_CH_2_N), 3.07 (p, *J* = 6.9 Hz, 1H, CH iPro), 3.81 (s, 4H, NCH_2_-pyr), 3.86 (t, *J* = 6.4 Hz, 2H, OCH_2_CH_2_CH_2_CH_2_CH_2_CH_2_N), 6.60–6.71 (m, 2H, Ar), 7.09 (d, *J* = 8.2 Hz, 1H, Ar), 7.26 (ddd, *J* = 7.5, 5.0, 1.3 Hz, 2H, Ar), 7.62 (dt, *J* = 7.9, 1.2 Hz, 2H, Ar), 7.76 (td, *J* = 7.7, 1.8 Hz, 2H, Ar), 8.43 (dd, *J* = 5.0, 1.8 Hz, 2H, Ar). ^13^C NMR (75 MHz, Chloroform-*d*) *δ* 47.7, 52.0, 55.1, 56.0, 56.1, 57.9, 58.5, 83.8, 89.3, 96.9, 141.2, 145.5, 151.9, 153.0, 154.7, 165.3, 166.8, 168.2, 177.5, 186.3, 188.7. Anal. calcd for C_28_H_37_N_3_O: C, 77.92; H, 8.64; N, 9.74. Found: C, 77.96; H, 8.68; N, 9.79.

##### General Procedure for the Synthesis of Compounds **32** and **33** (Series D)

Compounds **32** and **33** were synthesized via a coupling esterification reaction. Specifically, equimolar amounts of **1**, **2** and 3-carboxycoumarin acid (**31**), along with DCC (1.5 equivalents) and DMAP (0.5 equivalents), were dissolved in dry dichloromethane. The resulting pale-yellow mixture was stirred for 24 h. The reaction mixture was then filtered under reduced pressure to remove the dicyclohexylurea (DCU) byproduct, and the desired compounds were subsequently purified by column chromatography using a mobile phase of 10% ethyl acetate in *n*-hexane.

***4-chloro-2-isopropyl-5-methylphenyl 2-oxo-2H-chromene-3-carboxylate (*****32*****)***. White solid, m.p. 115–117 °C, 56% yield. ^1^H NMR (300 MHz, Chloroform-*d*) *δ* 1.21 (d, *J* = 6.9 Hz, 6H, 2 × CH_3_ iPro), 2.34 (s, 3H, ArCH_3_), 3.09 (m, 1H, CH iPro), 7.02 (s, 1H, Ar), 7.23–7.46 (m, 3H, Ar), 7.66–7.68 (m, 2H, Ar), 8.72 (s, 1H, Ar). ^13^C NMR (75 MHz, Chloroform-*d*) *δ* 19.8, 22.6, 23.1, 27.4, 117.1, 117.5, 117.9, 124.5, 125.2, 127.4, 130.0, 132.3, 134.7, 135.1, 139.6, 146.2, 150.2, 155.6, 156.5, 162.1. Anal. calcd for C_20_H_17_ClO_4_: C, 67.33; H, 4.80. Found: C, 67.30; H, 4.84.
***4-isopropyl-3-methylphenyl 2-oxo-2H-chromene-3-carboxylate (33).*** White solid, m.p. 128–130 °C, 88% yield. ^1^H NMR (300 MHz, Chloroform-*d*) *δ* 1.24 (m, 6H, 2 × CH_3_ iPro), 2.32–2.41 (m, 3H, ArCH_3_), 3.08–3.18 (m, 1H CH iPro), 7.04 (ddt, *J* = 8.6, 5.9, 3.5 Hz, 2H, Ar), 7.23–7.47 (m, 3H), Ar, 7.65–7.72 (m, 2H, Ar), 8.68–8.77 (m, 1H, Ar). ^13^C NMR (75 MHz, Chloroform-*d*) *δ* 19.5, 23.4, 25.1, 25.7, 29.1, 34.0, 117.0, 117.9, 118.0, 119.0, 122.9, 125.1, 125.9, 129.9, 134.9, 136.8, 145.0, 148.0, 149.8, 155.5, 156.6, 161.9. Anal. calcd for C_20_H_18_O_4_: C, 74.52; H, 5.63. Found: C, 74.59; H, 5.68.

### 3.2. CA Inhibition Assay

An Applied Photophysics stopped-flow (Applied Photophysics Limited, Leatherhead, UK) instrument measured the CA-catalyzed CO_2_ hydration activity [80]. The indicator used was phenol red (0.2 mM), with measurements taken at the absorbance maximum of 557 nm. Hepes (10 mM, pH 7.5) supplemented with 0.1 M Na_2_SO_4_ served as reaction buffer, and the CA-catalyzed CO_2_ hydration reaction was monitored for 10–100 s. The CO_2_ concentrations varied from 1.7 to 17 mM. The uncatalyzed rates were also measured and subtracted. Stock solutions of inhibitors (10 mM) were provided in distilled–deionized water containing 10% of DMSO and dilutions up to 0.001 μM were prepared with the reaction buffer. Inhibitor and enzyme solutions were preincubated together for 6 h at room temperature prior to assay in order to allow for the formation of the E-I complex. The inhibition constants were obtained by nonlinear least-squares methods using PRISM 3 (GraphPad, Boston, MA, USA, version 11.0.1) and the Cheng–Prusoff equation, representing the mean from at least three different determinations. The enzyme concentrations were in the range of 4–15 nM. All CA isoforms were commercially available or recombinant ones obtained *in-house*, as reported earlier [81].

### 3.3. Molecular Docking and Dynamics Simulation Studies

Molecular docking studies were performed using the Glide program within the Maestro Suite (Schrödinger Inc., New York, NY, USA). The crystal structures of the target proteins were obtained from the Protein Data Bank (PDB), with PDB ID: 6G9U for hCA IX [82] and 1JD0 for hCA XII [83]. The co-crystallized ligands, namely 4-[2-[3-(cyclooctylamino)-2,5,6-trifluoro-4-sulfamoylphenyl]sulfanylethyl]benzoic acid (sulfonamide) and 5-acetamido-1,3,4-thiadiazole-2-sulfonamide (acetazolamide, AAZ), were located in the active sites of the respective proteins. Active site analysis was carried out using the Maestro interface. Ligands were optimized using the LigPrep module with the MMFF94s force field. Protein preparation was performed using the Protein Preparation Wizard, applying default force field parameters. A total of 32 conformers per ligand were generated, and the top-ranked compound with its best-scoring conformer pose in complex with the protein was selected for further study and analysis.

We carried out molecular dynamics simulations using the Desmond V 5.9 software package from Schrödinger LLC. Initially, the docked complex was optimized with the OPLS force field. An orthorhombic cubic box was set up, and the complex was placed at its center. To maintain a distance of approximately 10 Å between the protein atoms and the box walls, TIP3P water molecules and buffers were added. Na^+^ and Cl^−^ counterions were introduced to establish the simulation environment, and the box volume was adjusted accordingly. The Desmond protocol as detailed in our previous paper, was used to minimize bias in the system using OPLS-2005 force field parameters. This was followed by an equilibration run to allow the system to adjust to the presence of oxygen molecules. The Berendsen NVT ensemble was maintained, with the system restrained to 10 K for the solute’s heavy atoms. The simulation was conducted at 300 K and 1 atm, with a relaxation time of 20 ps for both pressure and temperature, which were held constant. The Martyne–Tobias–Klein barostat and Nose-Hoover thermostat methods were utilized. The simulation was run with an NPT ensemble for approximately 100 ns. Frames were extracted from the simulation, and interaction diagrams were created to analyze trajectory fluctuations [84].

### 3.4. Cell-Based Assays

#### 3.4.1. Cell Cultures

MM cell lines used in this study were AMO, AMO-BZB, NCI-H929, and NCI-H929-BZB. The AMO cell line and its bortezomib-resistant counterpart, AMO-BZB, were kindly provided by Dr. C. Driessen (University of Tübingen, Germany), with AMO-BZB generated by exposure to stepwise increasing concentrations of bortezomib (BZB). The NCI-H929 cell line was obtained from DSMZ (Braunschweig, Germany), whereas the bortezomib-resistant NCI-H929-BZB cell line was generated by culturing NCI-H929 cells in the presence of gradually increasing concentrations of BZB, as previously described [85].

Human MM cell lines were cultured in RPMI-1640 medium (Corning, Corning, NY, USA) supplemented with 10% fetal bovine serum (FBS; Gibco^®^, Life Technologies, Carlsbad, CA, USA), 100 U/mL penicillin, and 100 µg/mL streptomycin (P/S; Gibco^®^, Life Technologies, Carlsbad, CA, USA). Cells were incubated at 37 °C in a humidified atmosphere containing 5% CO_2_. AMO-BZB and NCI-H929-BZB cells were maintained in the same medium as their parental isogenic counterparts, with the addition of 20 nM bortezomib to preserve resistance.

HEK-293 cells were cultured in DMEM (Corning, Corning, NY, USA) supplemented with 10% fetal bovine serum (FBS; Gibco^®^, Life Technologies, Carlsbad, CA, USA), 100 U/mL penicillin, and 100 µg/mL streptomycin (P/S; Gibco^®^, Life Technologies, Carlsbad, CA, USA), and maintained at 37 °C in a 5% CO_2_ atmosphere.

#### 3.4.2. Cell Viability and Apoptosis Assays

Cell viability was assessed using the CellTiter-Glo (CTG) assay kit (Promega, Madison, WI, USA), following the manufacturer’s instructions. MM and HEK-293T cells were seeded in 48-well plates and treated with selected compounds at increasing concentrations for 48 h. Luminescence was measured using a GloMax-Multi Detection System (Promega, Madison, WI, USA). IC_50_ values (mean ± SD) were calculated using GraphPad Prism software v. 10.2.0 from three independent experiments.

Apoptosis was evaluated by a flow cytometry-based assay (BD Biosciences, San Jose, CA, USA) using Annexin V conjugated to phycoerythrin (PE) and 7-aminoactinomycin D (7-AAD). MM cells were seeded in 24-well plates and treated with selected compounds for 48 h. Following treatment, cells were collected in 5 mL polystyrene tubes, washed with binding buffer, and stained with Annexin V-PE and 7-AAD for 15 min at room temperature in the dark. Data were acquired using a BD FACS Fortessa X-20 flow cytometer (BD Biosciences, San Jose, CA, USA) and analyzed with FlowJo software (version 10). A representative experiment out of three independent experiments is presented [21].

### 3.5. Antioxidant Assays

The antioxidant and chelating capacity were evaluated using a series of in vitro tests based on the experimental framework of a previous study [86]. The compounds were screened for reducing power using the FRAP and CUPRAC assays, and for radical scavenging activity using the DPPH and ABTS assays. The results of these four assays were quantified and standardized against the antioxidant Trolox and expressed as milligrams of Trolox equivalent per gram of compound (mg TE/g). Finally, the ability of the compounds to bind metal ions was evaluated using a metal chelation assay, with activity reported as milligrams of EDTA equivalent per gram of compound (mg EDTAE/g). Triplicate measurements were taken for all experiments, and differences across compounds were tested for statistical significance. Analysis was carried out using one-way ANOVA (GraphPad Prism, version 9.2), followed by Tukey’s post hoc analysis to compare individual groups. A *p*-value of less than 0.05 was considered statistically significant (and characterized by a different letter in Table 4).

## 4. Conclusions

In summary, we designed, synthesized, and biologically investigated a new series of chlorothymol- and 4-isopropyl-3-methylphenol-based derivatives incorporating either a coumarin or a dipicolylamine scaffold, with the aim of targeting the tumor-associated CA isoforms IX and XII. The results clearly demonstrate that conjugation of synthetic phenolic monoterpenes with the coumarin scaffold represents an effective strategy to achieve potent and selective inhibition of the cancer-related CA isoforms, whereas the dipicolylamine moiety failed to produce effective inhibitory activity against any of the hCA isoforms investigated herein. Among the coumarin-based derivatives, compounds **7**, **9**, **13**, and **20** exhibited strong potency and selectivity toward hCA IX and hCA XII. In particular, compounds **9** and **20** were further investigated through molecular docking and molecular dynamics simulations, which provided mechanistic insight into their binding modes and isoform selectivity. These studies highlighted the critical role of stereochemistry and substituent orientation in stabilizing key interactions with residues located at the active-site entrance, notably Thr199, Gln67, and His94. Notably, the *E* isomer of compound **20** displayed a stable and persistent binding mode within hCA IX throughout the MD simulations, supporting its preferential selectivity for this isoform. Importantly, selected compounds (**7**, **9**, **13**, and **20**) also demonstrated biologically relevant activity in cellular models of multiple myeloma, including proteasome inhibitor-resistant cell lines. Among them, compound **13** significantly reduced MM cell viability and induced potent apoptotic effects in both drug-sensitive and drug-resistant cells, while maintaining a favorable selectivity profile over non-tumor cells. Collectively, these findings further support the therapeutic potential of targeting hCA IX and hCA XII and suggest that CA inhibition may contribute to overcoming mechanisms of drug resistance. Further investigations in co-culture systems that better recapitulate the MM microenvironment, as well as in advanced in vivo models, will be required to refine the specificity profile of the selected compounds, better define their in vivo therapeutic potential, and characterize their pharmacokinetic and pharmacodynamic properties in the oncologic setting. Following the suggestions from in silico and in vitro analyses, a more suitable selection of the linker can be optimized. Lastly, antioxidant activity was assessed for these hybrids highlighting the importance of the **DPA**-based hybrids for this property.

## Figures and Tables

**Table 1:** Inhibitory activity of parent compounds (**1**–**3**, **22**, **31**), intermediate (**12**), reference drug acetazolamide (**AAZ**), and final derivatives against four human CA isoforms.

Compound	Structure	K_I_ (nM) *			
		hCA I	hCA II	hCA IX	hCA XII
Chloro thymol **1**	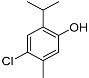	91,300	55,850	66,500	34,700
4-isopropyl-3-methylphenol **2**	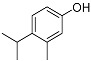	>100,000	>100,000	38,800	24,900
Umbelliferon (**UMB**) **3**	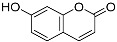	>10,000	>10,000	24.9	45.1 [47]
**4**	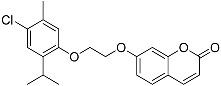	>100,000	>100,000	30.4	85.8
**5**	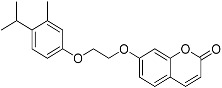	>100,000	>100,000	93.8	390.3
**6**	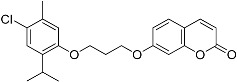	>100,000	>100,000	56.9	484.1
**7**	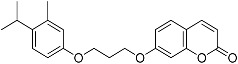	>100,000	>100,000	22.8	81.4
**8**	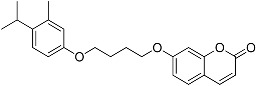	>100,000	>100,000	63.9	195.7
**9**	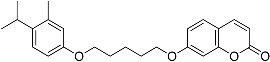	>100,000	>100,000	29.2	86.8
**10**	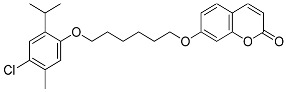	>100,000	>100,000	61.8	213.4
**11**	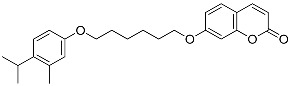	>100,000	>100,000	29.7	279.7
**12**	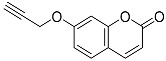	>10,000	>10,000	1350	730 [47]
**13**	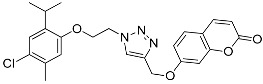	>100,000	>100,000	27.6	418.4
**14**	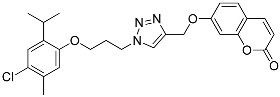	>100,000	>100,000	64.9	217.0
**15**	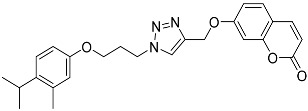	>100,000	>100,000	178.3	82.4
**16**	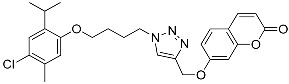	>100,000	>100,000	92.1	458.6
**17**	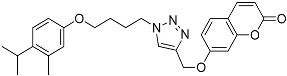	>100,000	>100,000	61.5	91.8
**18**	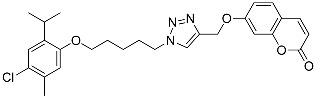	>100,000	>100,000	65.9	281.7
**19**	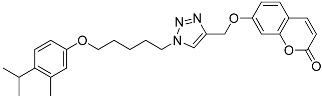	>100,000	>100,000	42.3	372.6
**20**	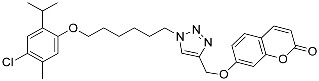	>100,000	>100,000	32.0	9.3
**21**	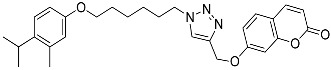	>100,000	>100,000	200.0	71.9
**22** **DPA**	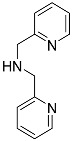	>100,000	>100,000	>100,000	>100,000
**23**	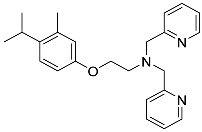	>100,000	>100,000	>100,000	>100,000
**24**	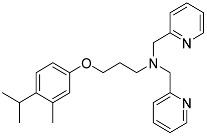	>100,000	>100,000	>100,000	>100,000
**25**	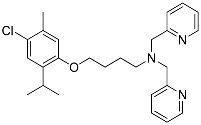	>100,000	>100,000	>100,000	>100,000
**26**	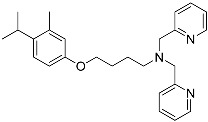	>100,000	>100,000	>100,000	>100,000
**27**	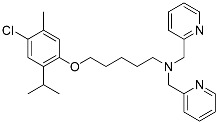	>100,000	>100,000	>100,000	>100,000
**28**	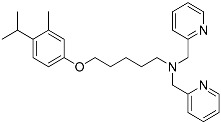	>100,000	>100,000	>100,000	>100,000
**29**	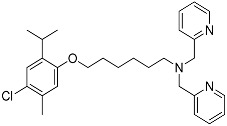	>100,000	>100,000	>100,000	>100,000
**30**	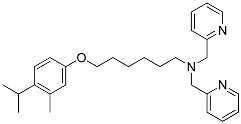	>100,000	>100,000	>100,000	>100,000
**31**	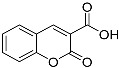	93,000	447,000	47,000	9000 [67]
**32**	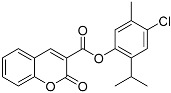	>100,000	>100,000	2295	4589
**33**	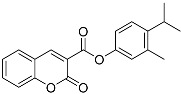	>100,000	>100,000	2934	6940
**Acetazolamide** **AAZ**	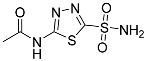	250.0	12.1	25.7	5.7

* Mean from 3 different assays by a stopped flow technique (errors were in the range of ±5–10% of the reported values).

**Table 2:** Binding energies of the best performing compounds (**9** and **20**).

Compound	hCA IX Binding Energy	hCA XII Binding Energy
**9**(*E*)	−7.352 kcal/mol	−6.328 kcal/mol
**9**(*Z*)	−7.345 kcal/mol	−5.959 kcal/mol
**20**(*E*)	−7.353 kcal/mol	−4.741 kcal/mol
**20**(*Z*)	−6.727 kcal/mol	−4.861 kcal/mol

**Table 3:** IC_50_ values were determined for the reported compounds in proteosome inhibitor (PI) sensitive (AMO, H929) and PI resistant (ABZB, H929-BZB) MM cell lines, 48 h after treatment. IC_50_ values (mean ± SD) were calculated using GraphPad Prism software v. 10.2.0 from three independent experiments.

	Multiple Myeloma Cell Lines (IC_50_, µM)			
	AMO	ABZB	H929	H929-BZB
**1** Chlorothymol	14.9 ± 0.6	14.1 ± 4.4	9.8 ± 0.2	17.5 ± 0.2
**2** 4-isopropyl-3-methylphenol	47.5 ± 5.1	>100	40.9 ± 1.6	91.5 ± 1.4
**3 (UMB)** Umbelliferon	>200	>100	>200	>200
**7**	76.9 ± 0.8	85.4 ± 5.2	57.3 ± 1.5	38.6 ± 4.5
**9**	>200	>100	>100	70.0 ± 2.5
**13**	15.8 ± 6.1	58.6 ± 0.1	19.5 ± 4.1	27.5 ± 5.6
**20**	>200	>100	>200	>200
**AAZ**	>200	>100	>200	>200

**Table 4:** The antioxidant properties of the tested compounds *.

Compounds	DPPH (mg TE/g)	ABTS (mg TE/g)	CUPRAC (mg TE/g)	FRAP (mg TE/g)	Chelating (mg EDTAE/g)
**4**	4.33 ± 0.13 ^de^	28.46 ± 0.63 ^j^	154.06 ± 0.71 ^i^	13.66 ± 0.49 ^kl^	na
**5**	na	18.04 ± 0.32 ^kl^	123.68 ± 1.76 ^l^	46.09 ± 2.13 ^hi^	6.62 ± 0.18 ^g^
**6**	na	16.49 ± 0.75 ^kl^	181.34 ± 0.96 ^d^	50.01 ± 3.29 ^ghi^	na
**7**	2.25 ± 0.14 ^fg^	28.67 ± 1.94 ^j^	181.25 ± 2.69 ^d^	59.71 ± 0.59 ^de^	na
**8**	na	13.93 ± 1.78 ^lm^	179.91 ± 0.65 ^de^	68.38 ± 3.74 ^c^	39.98 ± 0.15 ^c^
**9**	2.31 ± 0.39 ^fg^	27.64 ± 1.93 ^j^	143.48 ± 2.56 ^j^	19.41 ± 0.65 ^k^	na
**10**	2.47 ± 0.55 ^fg^	25.48 ± 2.17 ^j^	145.25 ± 2.08 ^j^	15.39 ± 0.75 ^kl^	na
**11**	1.49 ± 0.56 ^gh^	28.20 ± 2.36 ^j^	161.28 ± 0.43 ^h^	29.81 ± 1.56 ^j^	na
**13**	na	9.58 ± 1.03 ^mn^	85.79 ± 1.56 ^m^	28.53 ± 1.67 ^j^	na
**14**	0.54 ± 0.10 ^hi^	16.26 ± 1.61 ^kl^	144.41 ± 0.68 ^j^	56.97 ± 2.92 ^ef^	na
**15**	0.47 ± 0.01 ^hi^	14.51 ± 0.45 ^kl^	125.98 ± 0.29 ^l^	48.73 ± 1.63 ^ghi^	na
**16**	3.55 ± 0.90 ^ef^	41.27 ± 1.22 ^h^	76.95 ± 2.75 ^n^	6.52 ± 0.28 ^mn^	na
**17**	na	6.75 ± 0.04 ^no^	181.71 ± 1.14 ^d^	82.00 ± 4.52 ^ab^	na
**18**	na	7.97 ± 0.51 ^no^	164.35 ± 1.28 ^gh^	65.70 ± 2.22 ^cd^	na
**19**	na	5.58 ± 0.12 ^no^	170.72 ± 0.22 ^fg^	53.95 ± 5.30 ^efg^	na
**20**	na	17.87 ± 0.13 ^kl^	185.96 ± 1.95 ^d^	77.60 ± 0.10 ^b^	8.69 ± 0.50 ^fg^
**21**	na	3.50 ± 0.38 ^o^	169.99 ± 2.90 ^fg^	68.59 ± 1.47 ^c^	na
**23**	13.12 ± 1.15 ^a^	92.44 ± 0.76 ^a^	286.38 ± 2.99 ^a^	85.76 ± 0.14 ^a^	48.12 ± 3.33 ^a^
**24**	11.39 ± 1.04 ^b^	82.75 ± 0.74 ^b^	251.48 ± 2.82 ^b^	51.23 ± 0.03 ^fgh^	38.75 ± 2.37 ^c^
**25**	12.63 ± 0.48 ^ab^	54.11 ± 1.71 ^f^	135.20 ± 4.14 ^k^	44.93 ± 0.04 ^i^	9.00 ± 0.70 ^fg^
**26**	9.45 ± 0.65 ^c^	68.10 ± 2.01 ^d^	56.19 ± 0.48 ^o^	16.58 ± 0.01 ^kl^	44.79 ± 3.03 ^b^
**27**	9.46 ± 0.86 ^c^	62.27 ± 0.99 ^e^	83.76 ± 0.50 ^mn^	13.44 ± 0.40 ^kl^	42.61 ± 0.50 ^b^
**28**	3.93 ± 0.08 ^e^	47.37 ± 0.25 ^g^	36.51 ± 1.89 ^p^	12.10 ± 0.05 ^lm^	19.14 ± 2.27 ^e^
**29**	na	46.39 ± 4.14 ^g^	14.79 ± 2.75 ^q^	4.58 ± 0.01 ^n^	32.97 ± 0.34 ^d^
**30**	4.76 ± 0.11 ^de^	36.14 ± 0.80 ^i^	41.05 ± 0.02 ^p^	10.33 ± 0.19 ^lmn^	na
**32**	5.56 ± 0.21 ^d^	75.17 ± 0.28 ^c^	228.15 ± 4.74 ^c^	50.24 ± 1.35 ^ghi^	9.56 ± 0.68 ^f^
**33**	na	18.78 ± 0.67 ^k^	174.06 ± 2.94 ^ef^	69.43 ± 0.10 ^c^	na

* Values are reported as mean ± SD of three parallel measurements. TE: Trolox equivalent; EDTAE: EDTA equivalent. na: not active. Different letters indicate the differences between the tested compounds (ANOVA assay with Tukey’s post hoc analysis, *p* < 0.05).

## Data Availability

The original contributions presented in this study are included in the article/Supplementary Material. Further inquiries can be directed to the corresponding author.

## Supplementary Materials

The following supporting information can be downloaded at: https://www.mdpi.com/article/10.3390/ph19050717/s1, Figure S1. Ligand–receptor interaction diagrams of the coumarin- and **DPA**-based compounds. Amino acids and metal cofactor are color-coded based on their physicochemical properties: hydrophobic residues (green), polar residues (blue), positively charged residues (orange), negatively charged residues (red), and zinc metal (grey); Table S1. IC_50_ values were determined for the reported compounds in HEK293 cells, 48 h after treatment. IC_50_ values (mean ± SD) were calculated using GraphPad Prism software v. 10.2.0 from three independent experiments. Selectivity Index (SI) was calculated by the ratio of the corresponding IC_50_ value for each cell line; NMR spectra of the newly synthesized compounds.

## References

[1] Luo Q., Smith D.P. (2025). Global cancer burden: Progress, projections, and challenges. Lancet.

[2] Vishakha S., Navneesh N., Kurmi B.D., Gupta G.D., Verma S.K., Jain A., Patel P. (2024). An Expedition on Synthetic Methodology of FDA-approved Anticancer Drugs (2018–2021). Anticancer Agents Med. Chem..

[3] Roma-Rodrigues C., Mendes R., Baptista P.V., Fernandes A.R. (2019). Targeting Tumor Microenvironment for Cancer Therapy. Int. J. Mol. Sci..

[4] Angeli A., Carta F., Nocentini A., Winum J.Y., Zalubovskis R., Akdemir A., Onnis V., Eldehna W.M., Capasso C., Simone G. (2020). Carbonic Anhydrase Inhibitors Targeting Metabolism and Tumor Microenvironment. Metabolites.

[5] Robat-Jazi B., Mahalleh M., Dashti M., Nejati N., Ahmadpour M., Alinejad E., Mohammadi S., Lorestani P., Hamidieh A.A., Habibi M.A. (2025). A Systematic Review and Meta-analysis on the Safety and Efficacy of CAR T Cell Therapy Targeting GPRC5D in Patients with Multiple Myeloma: A New Insight in Cancer Immunotherapy. Anticancer Agents Med. Chem..

[6] Camacho X., Cabrera M., Perroni C., Tassano M., Fernández M., Oddone N., Riva E., Gambini J.P., Cabral P. (2026). Theranostic Potential of [177Lu]Lu-DOTA-Tocilizumab in Multiple Myeloma: A Preclinical Evaluation. Anticancer Agents Med. Chem..

[7] Radandish M., Mashhadi N., Aghayan A.H., Taghizadeh M., Salehianfard S., Yahyazadeh S., Vakili O., Igder S. (2025). In-depth insight into tumor-infiltrating stromal cells linked to tertiary lymphoid structures and their prospective function in cancer immunotherapy. Exp. Hematol. Oncol..

[8] Qian Y.R., Liu P., Xu H., Lv Y., Zhang X.F., Xiang J.X. (2026). Microenvironment plays a critical role in modulating tumor cell dormancy: Current perspectives and potential treatment options. World J. Clin. Oncol..

[9] Liao S., Wu G., Xie Z., Lei X., Yang X., Huang S., Deng X., Wang Z., Tang G. (2024). pH regulators and their inhibitors in tumor microenvironment. Eur. J. Med. Chem..

[10] Flinck M., Kramer S.H., Pedersen S.F. (2018). Roles of pH in control of cell proliferation. Acta Physiol..

[11] Hunter A., Hendrikse A., Renan M., Abratt R. (2006). Does the tumor microenvironment influence radiation-induced apoptosis?. Apoptosis.

[12] Dhup S., Dadhich R.K., Porporato P.E., Sonveaux P. (2012). Multiple biological activities of lactic acid in cancer: Influences on tumor growth, angiogenesis and metastasis. Curr. Pharm. Des..

[13] Kato Y., Ozawa S., Miyamoto C., Maehata Y., Suzuki A., Maeda T., Baba Y. (2013). Acidic extracellular microenvironment and cancer. Cancer Cell Int..

[14] Yu M., Yang D., Chen X., Yang Y., Zhang B., Jiang X., Xing L., Yang Y., Sun Y., Li N. (2026). Metabolic reprogramming in cancer: Dysregulation of glucose, lipid, and amino acid pathways and therapeutic opportunities. Mol. Biomed..

[15] Kalinin S., Malkova A., Sharonova T., Sharoyko V., Bunev A., Supuran C.T., Krasavin M. (2021). Carbonic Anhydrase IX Inhibitors as Candidates for Combination Therapy of Solid Tumors. Int. J. Mol. Sci..

[16] Yang H., Chen R., Zheng X., Luo Y., Yao M., Ke F., Guo X., Liu X., Liu Q. (2025). Cooperative Role of Carbonic Anhydrase IX/XII in Driving Tumor Invasion and Metastasis: A Novel Targeted Therapeutic Strategy. Cells.

[17] Wykoff C.C., Beasley N.J., Watson P.H., Turner K.J., Pastorek J., Sibtain A., Wilson G.D., Turley H., Talks K.L., Maxwell P.H. (2000). Hypoxia-inducible expression of tumor-associated carbonic anhydrases. Cancer Res..

[18] McDonald P.C., Dedhar S. (2014). Carbonic anhydrase IX (CAIX) as a mediator of hypoxia-induced stress response in cancer cells. Subcell. Biochem..

[19] Gastelum G., Kraut J., Veena M., Baibussinov A., Lamb C., Lyons K., Chang E.Y., Frost P. (2023). Acidification of intracellular pH in MM tumor cells overcomes resistance to hypoxia-mediated apoptosis in vitro and in vivo. Front. Oncol..

[20] Filippi I., Saltarella I., Aldinucci C., Carraro F., Ria R., Vacca A., Naldini A. (2018). Different Adaptive Responses to Hypoxia in Normal and Multiple Myeloma Endothelial Cells. Cell. Physiol. Biochem..

[21] Melfi F., D’Agostino I., Carradori S., Carta F., Angeli A., Costa G., Renzi G., Čikoš A., Vullo D., Rešetar J. (2025). O-derivatization of natural tropolone and β-thujaplicin leading to effective inhibitors of human carbonic anhydrases IX and XII. Eur. J. Med. Chem..

[22] Martin S.K., Diamond P., Gronthos S., Peet D.J., Zannettino A.C. (2011). The emerging role of hypoxia, HIF-1 and HIF-2 in multiple myeloma. Leukemia.

[23] Alipoor S.D., Shrestha M., Liu A., Chang H. (2026). Deciphering epigenetic crosstalk in multiple myeloma pathogenesis and treatment. Clin. Epigenetics.

[24] Hu A., Chen H., Liang J., Liu C., Li F., Mu C. (2021). Cell-based therapeutics for the treatment of hematologic diseases inside the bone marrow. J. Control. Release.

[25] Paradzik T., Bandini C., Mereu E., Labrador M., Taiana E., Amodio N., Neri A., Piva R. (2021). The Landscape of Signaling Pathways and Proteasome Inhibitors Combinations in Multiple Myeloma. Cancers.

[26] Bilski R., Kupczyk D., Kaczorowska-Bilska K., Tkaczenko H., Kurhaluk N., Kosmalski T., Słomka A., Studzińska R. (2026). Oxidative Stress in Multiple Myeloma: Pathogenic Mechanisms, Biomarkers, and Redox-Targeted Therapeutic Strategies. Int. J. Mol. Sci..

[27] Ikeda S., Tagawa H. (2021). Impact of hypoxia on the pathogenesis and therapy resistance in multiple myeloma. Cancer Sci..

[28] Muz B., de la Puente P., Azab F., Luderer M., Azab A.K. (2014). Hypoxia promotes stem cell-like phenotype in multiple myeloma cells. Blood Cancer J..

[29] Tiwari A., Trivedi R., Lin S.Y. (2022). Tumor microenvironment: Barrier or opportunity towards effective cancer therapy. J. Biomed. Sci..

[30] De Raeve H.R., Vermeulen P.B., Vanderkerken K., Harris A.L., Van Marck E. (2004). Microvessel density, endothelial-cell proliferation and carbonic anhydrase IX expression in haematological malignancies, bone-marrow metastases and monoclonal gammopathy of undetermined significance. Virchows Arch..

[31] Petrilla C., Galloway J., Kudalkar R., Ismael A., Cottini F. (2023). Understanding DNA Damage Response and DNA Repair in Multiple Myeloma. Cancers.

[32] Caillot M., Dakik H., Mazurier F., Sola B. (2021). Targeting Reactive Oxygen Species Metabolism to Induce Myeloma Cell Death. Cancers.

[33] Yen C.H., Hsu C.M., Hsiao S.Y., Hsiao H.H. (2020). Pathogenic Mechanisms of Myeloma Bone Disease and Possible Roles for NRF2. Int. J. Mol. Sci..

[34] Xu J., Dong X., Dong J., Peng Y., Xing M., Chen L., Zhao Q., Chen B. (2024). Leveraging diverse cellular stress patterns for predicting clinical outcomes and therapeutic responses in patients with multiple myeloma. J. Cell. Mol. Med..

[35] Liang Q., Zhang L., Huang Q., Lv W., Liang Z., Liu S., Nie R., Xia Z., Liang Y., Wang Y. (2025). Oxidative Stress Score as a Simplified Surrogate for Prognostic Stratification and Therapeutic Decision-Making in Multiple Myeloma. Pharmaceuticals.

[36] Abe K., Ikeda S., Nara M., Kitadate A., Tagawa H., Takahashi N. (2023). Hypoxia-induced oxidative stress promotes therapy resistance via upregulation of heme oxygenase-1 in multiple myeloma. Cancer Med..

[37] Gastelum G., Veena M., Lyons K., Lamb C., Jacobs N., Yamada A., Baibussinov A., Sarafyan M., Shamis R., Kraut J. (2021). Can Targeting Hypoxia-Mediated Acidification of the Bone Marrow Microenvironment Kill Myeloma Tumor Cells?. Front. Oncol..

[38] Lin Z., Cheng X., Zheng H. (2023). Umbelliferon: A review of its pharmacology, toxicity and pharmacokinetics. Inflammopharmacology.

[39] Lacy A., O’Kennedy R. (2004). Studies on coumarins and coumarin-related compounds to determine their therapeutic role in the treatment of cancer. Curr. Pharm. Des..

[40] Sharma Y., Kalra S., Vashisht A., Sharma R. (2025). Coumarin-based Strategies for Breast Cancer: A Multifaceted Perspective. Mini Rev. Med. Chem..

[41] Sharmah H., Ahmed L.A., Kemisetti D., Kumar S., Samanthula K.S., Panigrahy U.P., Ghosh N.S. (2026). Current advances in 7-hydroxycoumarin derivatives as potential therapeutic agents for Alzheimer’s disease. Mol. Divers..

[42] Singh S., Kaushik N., Paliwal A., Sengar M.S., Paliwal D. (2025). Biological Activity and Therapeutic Potential of Coumarin Derivatives: A Comprehensive Review. Curr. Drug Discov. Technol..

[43] Peter S., Sibali L.L. (2025). Recent Developments on Coumarin Hybrids as Antimicrobial Agents. Antibiotics.

[44] Chavez Alvarez A.C., Moreau E. (2026). Coumarin-Based Prodrugs: Therapeutic Promise or Still Confined to Preclinical Exploration?. Pharmaceutics.

[45] Moi D., Carradori S., Gallorini M., Mencarelli N., Deplano A., Angeli A., Vittorio S., Supuran C.T., Onnis V. (2025). Investigation on Human Carbonic Anhydrase IX and XII Inhibitory Activity and A549 Antiproliferative Activity of a New Class of Coumarinamides. Pharmaceuticals.

[46] Redij A., Carradori S., Petreni A., Supuran C.T., Toraskar M.P. (2023). Coumarin-pyrazoline Hybrids as Selective Inhibitors of the Tumor-associated Carbonic Anhydrase IX and XII. Anticancer Agents Med. Chem..

[47] Melfi F., Mencarelli N., Carradori S., Gallorini M., Angeli A., Poli G., Cataldi A., D’Agostino I., Di Credico A., Di Baldassarre A. (2026). Hybridization Approach Applied to Umbelliferon and Vanilloids toward New Inhibitors of Carbonic Anhydrases IX and XII with In Vitro Antiproliferative and Anti-inflammatory Activities. J. Med. Chem..

[48] Rahman F., Wushur I., Malla N., Åstrand O.A.H., Rongved P., Winberg J.O., Sylte I. (2021). Zinc-Chelating Compounds as Inhibitors of Human and Bacterial Zinc Metalloproteases. Molecules.

[49] Kildahl-Andersen G., Schnaars C., Prandina A., Radix S., Le Borgne M., Jordheim L.P., Gjøen T., Andresen A.M.S., Lauksund S., Fröhlich C. (2019). Synthesis and biological evaluation of zinc chelating compounds as metallo-β-lactamase inhibitors. Med. Chem. Commun..

[50] Singh A.K., Kumar A., Singh H., Sonawane P., Paliwal H., Thareja S., Pathak P., Grishina M., Jaremko M., Emwas A.H. (2022). Concept of Hybrid Drugs and Recent Advancements in Anticancer Hybrids. Pharmaceuticals.

[51] Deng S., Wen X., Wang J. (2025). Recent Advances in the Linkers of Drug Conjugates. Curr. Med. Chem..

[52] Mishra K.B. (2025). 1,5-Disubstituted 1,2,3-triazoles: Molecular scaffolds for medicinal chemistry and biomolecular mimetics. Eur. J. Med. Chem..

[53] Khandelwal R., Vasava M., Abhirami R.B., Karsharma M. (2024). Recent advances in triazole synthesis via click chemistry and their pharmacological applications: A review. Bioorg. Med. Chem. Lett..

[54] Herrera-Bravo J., Belén L.H., Reyes M.E., Silva V., Fuentealba S., Paz C., Loren P., Salazar L.A., Sharifi-Rad J., Calina D. (2024). Thymol as adjuvant in oncology: Molecular mechanisms, therapeutic potentials, and prospects for integration in cancer management. Naunyn Schmiedebergs Arch. Pharmacol..

[55] Noman A.M., Sultan M.T., Zafar S., Maaz M., Mazhar A., Hussain M., Imran M., Mujtaba A., Hussain M.T., Alsagaby S.A. (2025). Thymol and Carvacrol: Molecular Mechanisms, Therapeutic Potential, and Synergy With Conventional Therapies in Cancer Management. Food Sci. Nutr..

[56] Patwa N., Singh G., Sharma V., Chaudhary P., Sharma B., Haque S., Yadav V., Satapathy S.R., Tuli H.S. (2025). Targeting Gastrointestinal Cancers with Carvacrol: Mechanistic Insights and Therapeutic Potential. Biomolecules.

[57] Musmula M., Akkoc S. (2026). Apoptotic effect of carvacrol on different cancer cells and its potential as an active medicine ingredient. J. Asian Nat. Prod. Res..

[58] Kornicka A., Balewski Ł., Lahutta M., Kokoszka J. (2023). Umbelliferone and Its Synthetic Derivatives as Suitable Molecules for the Development of Agents with Biological Activities: A Review of Their Pharmacological and Therapeutic Potential. Pharmaceuticals.

[59] Chen X., Lim C.S., Lee D., Lee S., Park S.J., Kim H.M., Yoon J. (2017). Two-photon fluorescence sensors for imaging NMDA receptors and monitoring release of Zn2+ from the presynaptic terminal. Biosens. Bioelectron..

[60] Xu H., Wang Z., Li Y., Ma S., Hu P., Zhong X. (2013). A quantum dot-based "off-on" fluorescent probe for biological detection of zinc ions. Analyst.

[61] Guan Q., Xing S., Wang L., Zhu J., Guo C., Xu C., Zhao Q., Wu Y., Chen Y., Sun H. (2024). Triazoles in Medicinal Chemistry: Physicochemical Properties, Bioisosterism, and Application. J. Med. Chem..

[62] Mizukami Y., Kakehi Y., Li F., Yamamoto T., Tajima K., Isono T., Satoh T. (2024). Chemically Recyclable Unnatural (1→6)-Polysaccharides from Cellulose-Derived Levoglucosenone and Dihydrolevoglucosenone. ACS Macro Lett..

[63] Citarella A., Amenta A., Passarella D., Micale N. (2022). Cyrene: A Green Solvent for the Synthesis of Bioactive Molecules and Functional Biomaterials. Int. J. Mol. Sci..

[64] Camp J.E. (2018). Bio-available Solvent Cyrene: Synthesis, Derivatization, and Applications. ChemSusChem.

[65] Kasana S., Nigam V., Singh S., Kurmi B.D., Patel P. (2024). A New Insight into The Huisgen Reaction: Heterogeneous Copper Catalyzed Azide-Alkyne Cycloaddition for the Synthesis of 1,4-Disubstituted Triazole (From 2018-2023). Chem. Biodivers..

[66] Munawar S., Zahoor A.F., Hussain S.M., Ahmad S., Mansha A., Parveen B., Ali K.G., Irfan A. (2023). Steglich esterification: A versatile synthetic approach toward the synthesis of natural products, their analogues/derivatives. Heliyon.

[67] Mahammad Ghouse S., Bahatam K., Angeli A., Pawar G., Chinchilli K.K., Yaddanapudi V.M., Mohammed A., Supuran C.T., Nanduri S. (2023). Synthesis and biological evaluation of new 3-substituted coumarin derivatives as selective inhibitors of human carbonic anhydrase IX and XII. J. Enzym. Inhib. Med. Chem..

[68] Saleem B.A.A., Qurtam A.A., Ahmed M., Al-Aouadi R.F.A., Alrikabi A.A.A., Hetta H.F., Bräse S., Alotaibi G., Alkhammash A., Farhan S.M. (2026). Discovery of a Novel Coumarin/Thiazole Chalcone Hybrid as a Potent Dual Inhibitor of Tubulin and Carbonic Anhydrases IX and XII with Promising Anti-Proliferative Activity. Molecules.

[69] Satyo L., Amoako D.G., Somboro A.M., Sosibo S.C., Kumalo H.M., Mhlongo N.N., Khan R.B. (2020). Molecular Insights Into Di(2-Picolyl) Amine-Induced Cytotoxicity and Apoptosis in Human Kidney (HEK293) Cells. Int. J. Toxicol..

[70] Wang J., Meng F., Chen Z., He Y., Kim J.S., Parra K.G., Jiang K., Sargent T.G., Yeo Y. (2025). Phosphatidylserine blockade by dipicolylamine-zinc enhances chemoimmunotherapy of B16F10 melanoma. J. Pharm. Sci..

[71] Liu S., Zhang M., Sun X., Wang P., Zhang G., Li W. (2025). Application of Hypoxia-Activated Prodrugs in Tumor Therapy: Design Strategies and Perspectives. J. Med. Chem..

[72] Maresca A., Temperini C., Vu H., Pham N.B., Poulsen S.A., Scozzafava A., Quinn R.J., Supuran C.T. (2009). Non-zinc mediated inhibition of carbonic anhydrases: Coumarins are a new class of suicide inhibitors. J. Am. Chem. Soc..

[73] Alhujaily M., Alahmari M., Asiri A., Shafie A. (2026). Advances in Coumarin-Based Dual-Mode Probes for Bioimaging and Cytotoxicity Evaluation: A Review. Crit. Rev. Anal. Chem..

[74] Leal L.E., Moreira E.S., Correia B.L., Bueno P.S.A., Comar J.F., de Sá-Nakanishi A.B., Cuman R.K.N., Bracht A., Bersani-Amado C.A., Bracht L. (2024). Comparative study of the antioxidant and anti-inflammatory effects of the natural coumarins 1,2-benzopyrone, umbelliferone and esculetin: In silico, in vitro and in vivo analyses. Naunyn Schmiedebergs Arch. Pharmacol..

[75] Delgado-Marín L., Sánchez-Borzone M., García D.A. (2017). Neuroprotective effects of gabaergic phenols correlated with their pharmacological and antioxidant properties. Life Sci..

[76] Allegra A., Petrarca C., Di Gioacchino M., Casciaro M., Musolino C., Gangemi S. (2022). Modulation of Cellular Redox Parameters for Improving Therapeutic Responses in Multiple Myeloma. Antioxidants.

[77] Xiong S., Chng W.J., Zhou J. (2021). Crosstalk between endoplasmic reticulum stress and oxidative stress: A dynamic duo in multiple myeloma. Cell. Mol. Life Sci..

[78] Admas S., Teketelew B.B., Alemu N., Marelgn L., Gelaw Y. (2026). The role of antioxidants in multiple myeloma: Diagnostic, prognostic, and therapeutic applications: A narrative review. Ther. Adv. Hematol..

[79] Pekarik V., Peskova M., Duben J., Remes M., Heger Z. (2020). Direct fluorogenic detection of palladium and platinum organometallic complexes with proteins and nucleic acids in polyacrylamide gels. Sci. Rep..

[80] Khalifah R.G. (1971). The carbon dioxide hydration activity of carbonic anhydrase. I. Stop flow kinetic studies on the native human isoenzymes B and C. J. Biol. Chem..

[81] Paciotti R., Carradori S., Angeli A., D’Agostino I., Ferraroni M., Coletti C., Supuran C.T. (2025). Unprecedented carbonic anhydrase inhibition mechanism: Targeting histidine 64 side chain through a halogen bond. Arch. Pharm..

[82] Kazokaitė J., Niemans R., Dudutienė V., Becker H.M., Leitāns J., Zubrienė A., Baranauskienė L., Gondi G., Zeidler R., Matulienė J. (2018). Novel fluorinated carbonic anhydrase IX inhibitors reduce hypoxia-induced acidification and clonogenic survival of cancer cells. Oncotarget.

[83] Whittington D.A., Waheed A., Ulmasov B., Shah G.N., Grubb J.H., Sly W.S., Christianson D.W. (2001). Crystal structure of the dimeric extracellular domain of human carbonic anhydrase XII, a bitopic membrane protein overexpressed in certain cancer tumor cells. Proc. Natl. Acad. Sci. USA.

[84] Saravanan V., Chagaleti B.K., Packiapalavesam S.D., Kathiravan M. (2024). Ligand based pharmacophore modelling and integrated computational approaches in the quest for small molecule inhibitors against hCA IX. RSC Adv..

[85] Torcasio R., Gallo Cantafio M.E., Veneziano C., De Marco C., Ganino L., Valentino I., Occhiuzzi M.A., Perrotta I.D., Mancuso T., Conforti F. (2024). Targeting of mitochondrial fission through natural flavanones elicits anti-myeloma activity. J. Transl. Med..

[86] Cairone F., Cesa S., Arpante I., Di Simone S.C., Mendez A.H., Ferrante C., Menghini L., Filippi A., Fraschetti C., Zengin G. (2025). Pomegranate Juices: Analytical and Bio-Toxicological Comparison of Pasteurization and High-Pressure Processing in the Development of Healthy Products. Foods.

