# Supplementary material for: Coumarin– and Dipicolylamine–Terpenoid Hybrids as Selective Carbonic Anhydrases IX and XII Inhibitors: Mechanistic Insights and Selective Anti-Cancer Potential
Source: Pharmaceuticals (Basel). 2026 Apr 30;19(5):717. doi: 10.3390/ph19050717 (PMC13209515; doi:10.3390/ph19050717)

Supporting Information

Coumarin– and Dipicolylamine–Terpenoid Hybrids as Selective Carbonic Anhydrases IX and XII Inhibitors: Mechanistic Insights and Selective Anti-Cancer Potential

Venkatesan Saravanan 1, Andrea Angeli 2,*, Francesco Melfi 3, Nicola Amodio 4, Ilenia Valentino 4, Massimo Gentile 5, Ilaria D'Agostino 6, Kathiravan Muthukumaradoss 7, Gokhan Zengin 8, Davide Moi 9, Rahime Simsek 10, Claudiu T. Supuran 2 and Simone Carradori 3

1 *School of Pharmacy, SBV Chennai, Sri Balaji Vidyapeeth, Pondicherry 607402, India; ven-kuu111@gmail.com*

2 *Neurofarba Department, University of Florence, Sesto Fiorentino, 50019 Florence, Italy; claudiu.supuran@unifi.it*

3 *Department of Pharmacy, "G. d'Annunzio" University of Chieti-Pescara, 66100 Chieti, Italy; francesco.melfi@unich.it (F.M.); simone.carradori@unich.it (S.C.)*

4 *Department of Experimental and Clinical Medicine, University "Magna Græcia" of Catanzaro, Campus "S. Venuta", 88100 Catanzaro, Italy; amodio@unicz.it (N.A.); ilenia.valentino@studenti.unicz.it (I.V.)*

5 *Department of Pharmacy, Health and Nutritional Science, University of Calabria, 87036 Rende, Italy; massimo.gentile@unical.it*

6 *Department of Pharmacy, University of Pisa, via Bonanno Pisano 6, 56126 Pisa, Italy; ilaria.dagostino@unipi.it*

7 *Department of Pharmaceutical Chemistry, Faculty of Medicine and Health Sciences, SRM Institute of Science and Technology, Kattankulathur, SRM College of Pharmacy, Chengalpattu District, Tamil Nadu 603203, India; kathirak@srmist.edu.in*

8 *Department of Biology, Science Faculty, Selcuk University, 42130 Konya, Türkiye; gokhanzengin@selcuk.edu.tr*

9 *Dipartimento di Scienze della Vita e dell'Ambiente, Cittadella Universitaria di Monserrato, Università degli Studi di Cagliari, S.P. 8 CA, 09042 Monserrato, Italy; davide.moi@unica.it*

10 *Department of Pharmaceutical Chemistry, Faculty of Pharmacy, Hacettepe University, 06100 Ankara, Türkiye; rsimsek@hacettepe.edu.tr*

* *Correspondence: andrea.angeli@unifi.it*

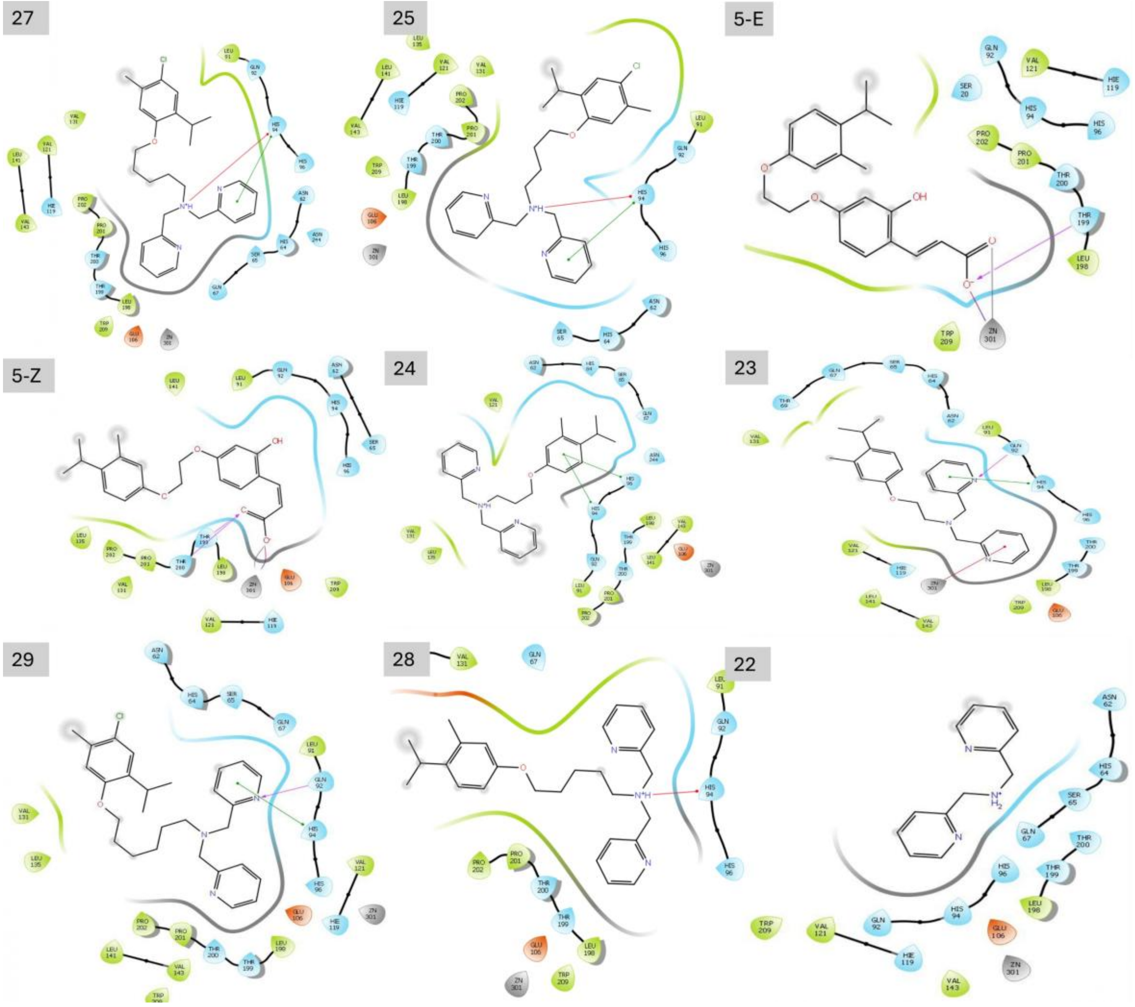

Figure S1. Ligand–receptor interaction diagrams of the coumarin- and **DPA**-based compounds. Amino acids and metal cofactor are color-coded based on their physicochemical properties: hydrophobic residues (green), polar residues (blue), positively charged residues (orange), negatively charged residues (red), and zinc metal (grey).

Table S1. IC_{50} values were determined for the reported compounds in HEK293 cells, 48 hours after treatment. IC_{50} values (mean ± SD) were calculated using GraphPad Prism software v. 10.2.0 from three independent experiments. Selectivity Index (SI) was calculated by the ratio of the corresponding IC_{50} value for each cell line.

	**HEK293 cells** (IC_{50}, μM)	**SI** (IC_{50} HEK293/IC_{50} AMO)	**SI** (IC_{50} HEK293/IC_{50} ABZB)	**SI** (IC_{50} HEK293/IC_{50} H929)	**SI** (IC_{50} HEK293/IC_{50} H929-BZB)
**1** Chlorothymol	32.7 ± 9.9	2.19	2.32	3.34	1.87
**2** 4-isopropyl-3-methylphenol	>200	>4.21	-	>4.89	>2.18
**3** Umbelliferon	>200	-	-	-	-
**7**	37.9 ± 4.4	0.49	0.44	0.66	0.98
**9**	>200	-	-	-	>2.85
**13**	34.6 ± 0.9	2.19	0.59	1.98	1.40
**20**	>200	-	-	-	-
**AAZ**	>200	-	-	-	-

NMR spectra of the newly synthesized compounds.

7-(2-(4-chloro-2-isopropyl-5-methylphenoxy)ethoxy)-2H-chromen-2-one (4)

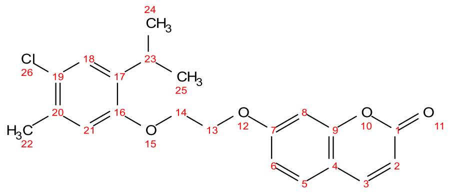

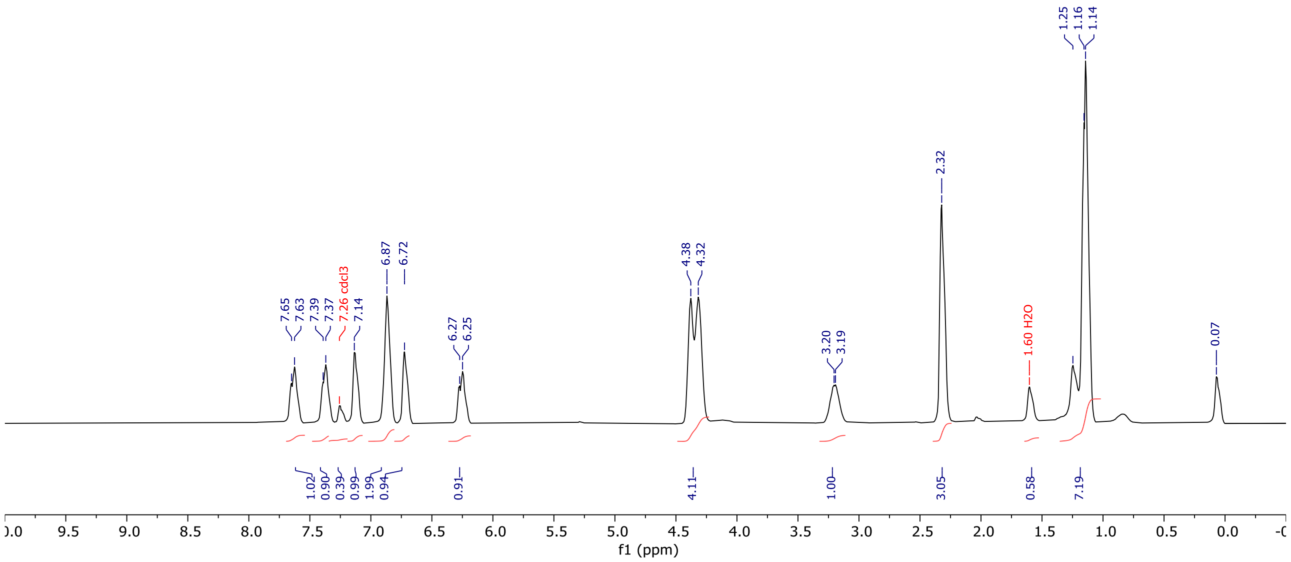

7-(2-(4-chloro-2-isopropyl-5-methylphenoxy)ethoxy)-2H-chromen-2-one (4)

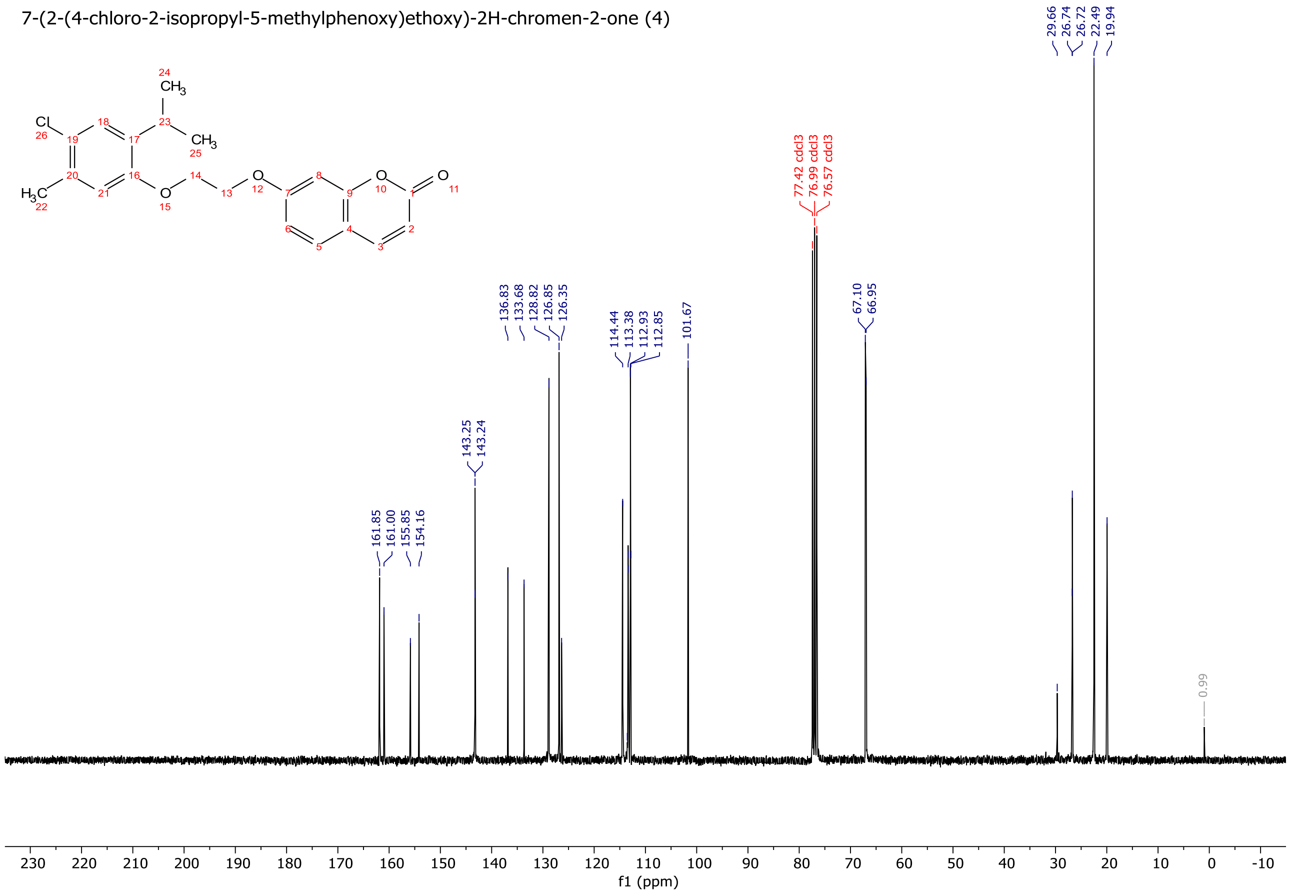

7-(2-(4-isopropyl-3-methylphenoxy)ethoxy)-2H-chromen-2-one (5)

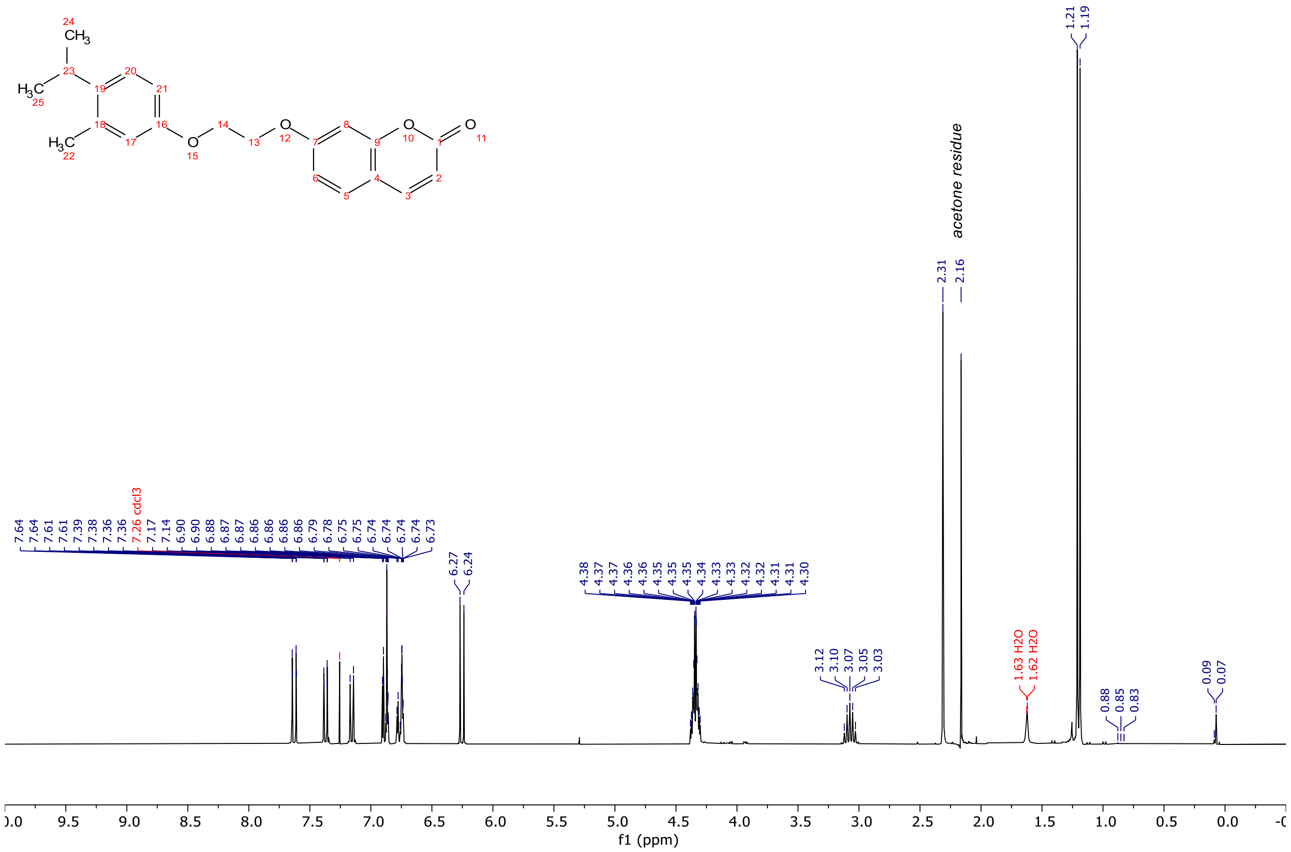

7-(2-(4-isopropyl-3-methylphenoxy)ethoxy)-2H-chromen-2-one (5)

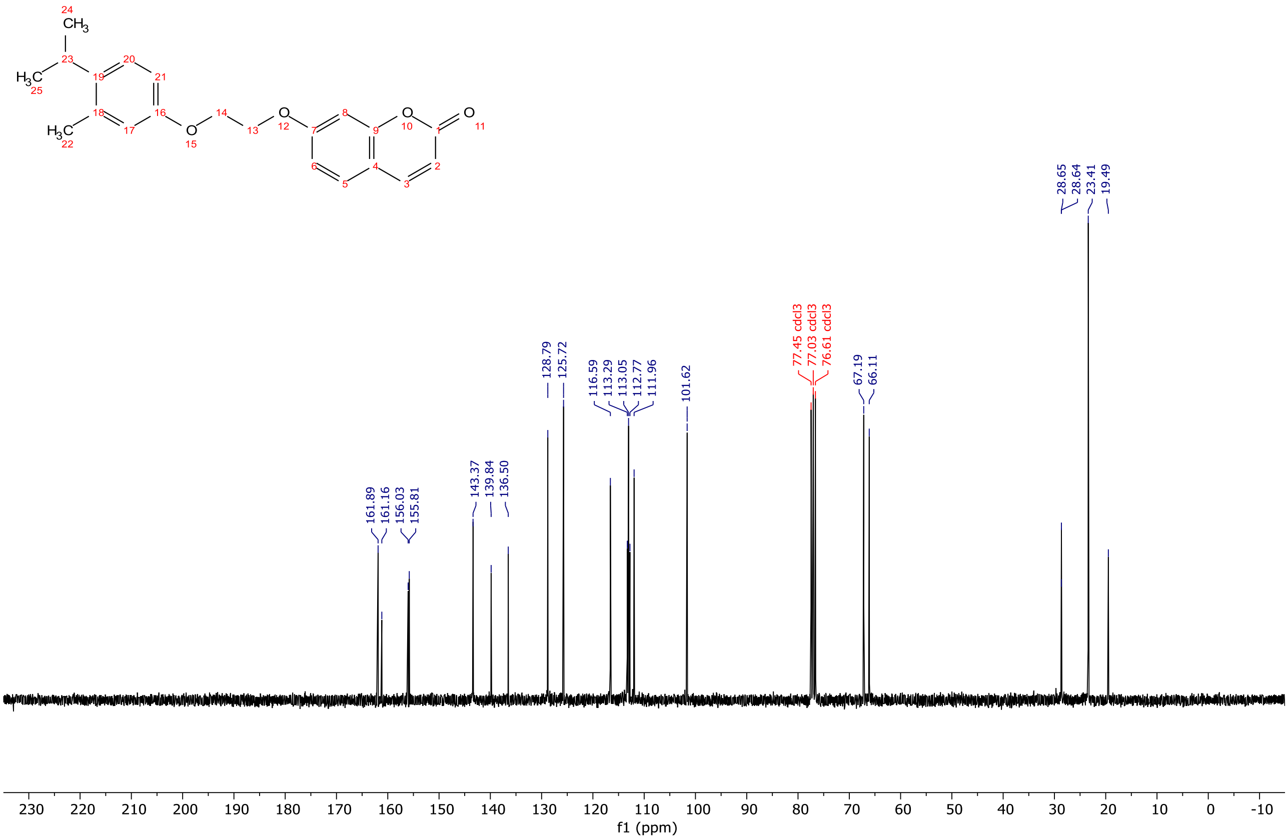

7-(3-(4-chloro-2-isopropyl-5-methylphenoxy)propoxy)-2H-chromen-2-one (6)

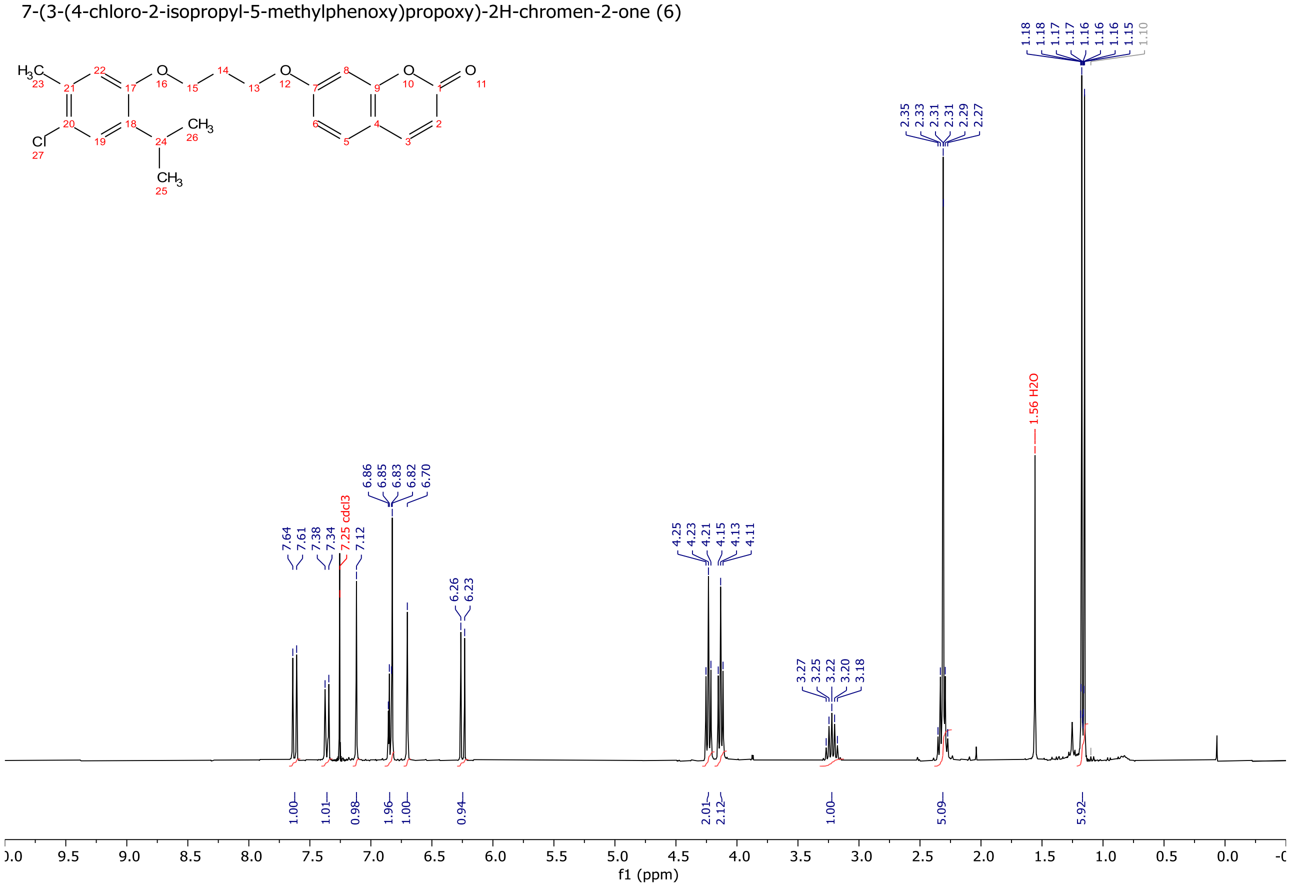

7-(3-(4-chloro-2-isopropyl-5-methylphenoxy)propoxy)-2H-chromen-2-one (6)

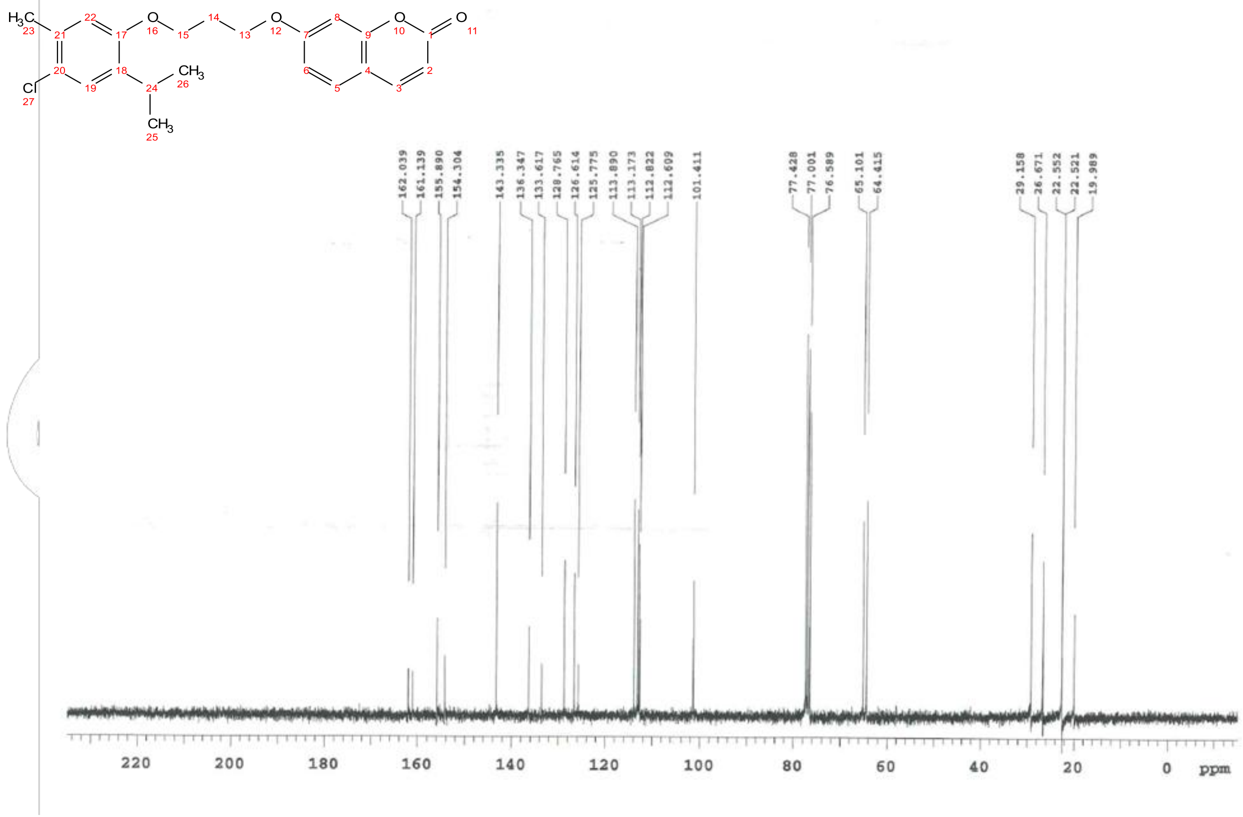

7-(3-(4-isopropyl-3-methylphenoxy)propoxy)-2H-chromen-2-one (7)

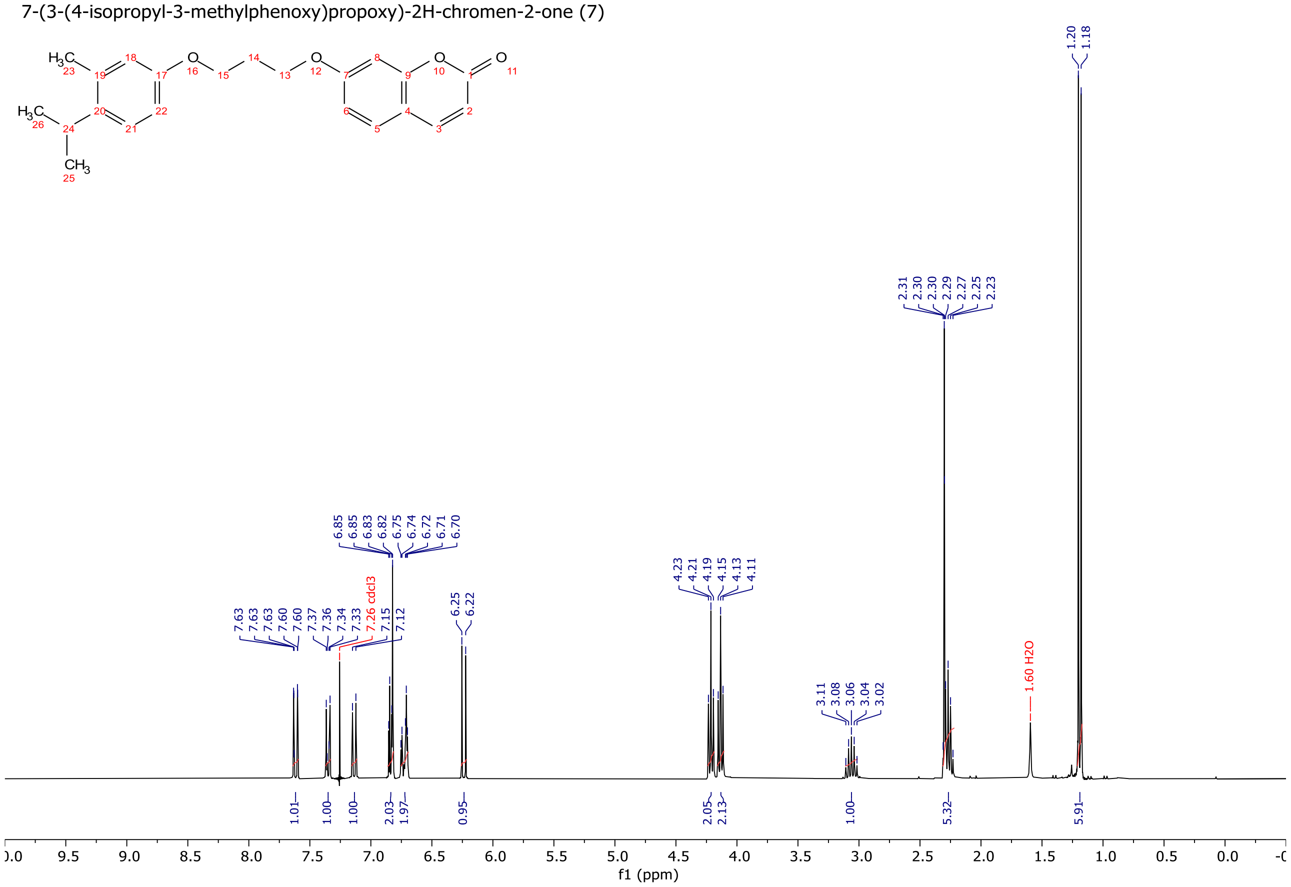

7-(3-(4-isopropyl-3-methylphenoxy)propoxy)-2H-chromen-2-one (7)

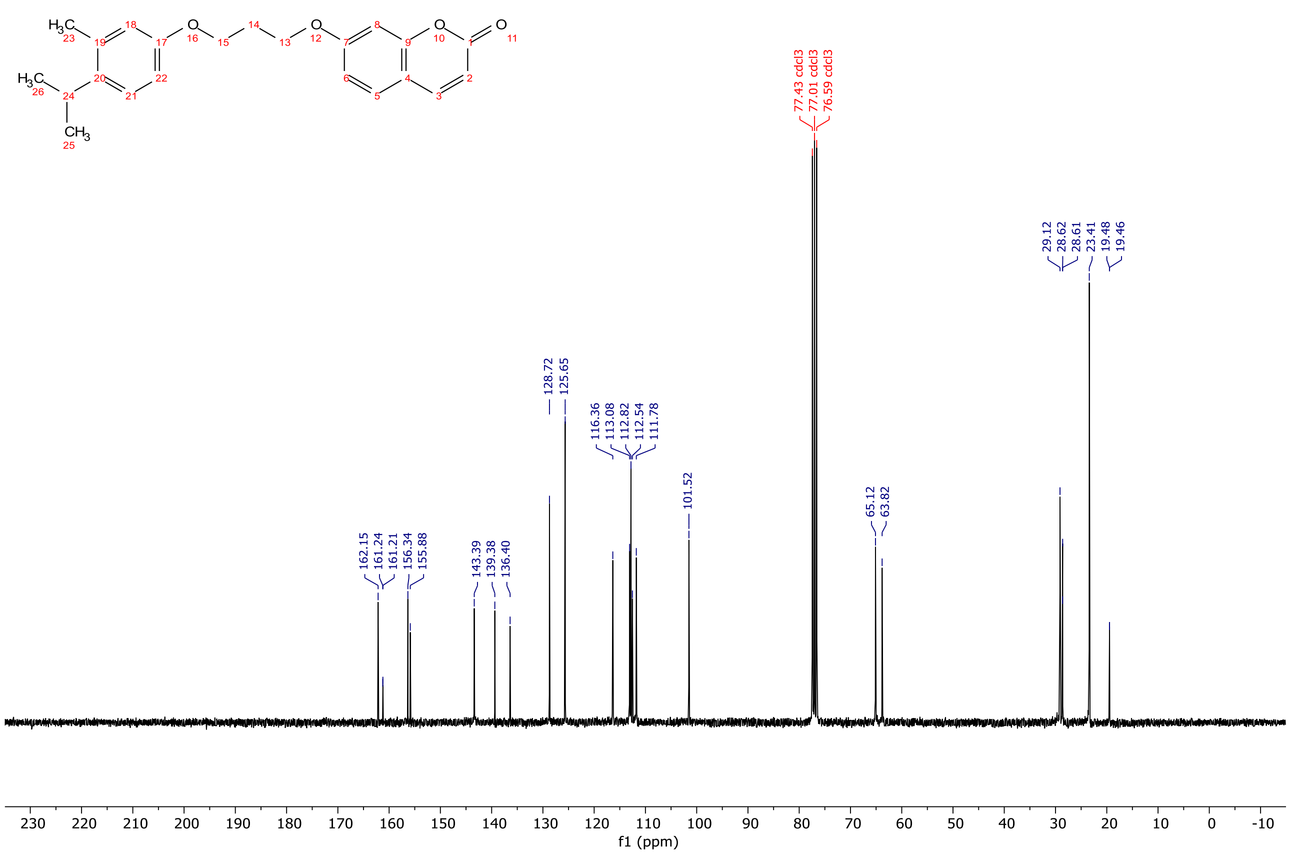

7-(4-(4-isopropyl-3-methylphenoxy)butoxy)-2H-chromen-2-one (8)

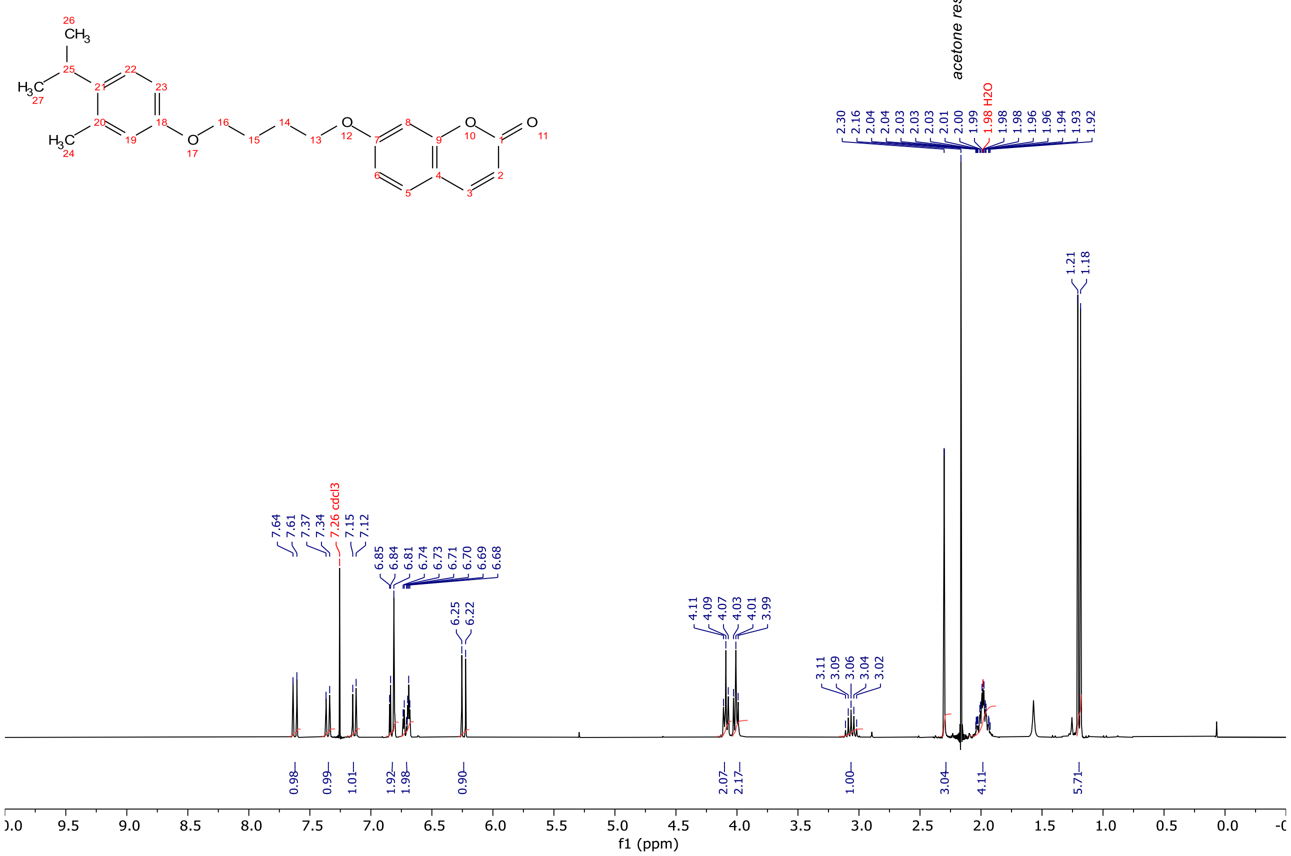

7-(4-(4-isopropyl-3-methylphenoxy)butoxy)-2H-chromen-2-one (8)

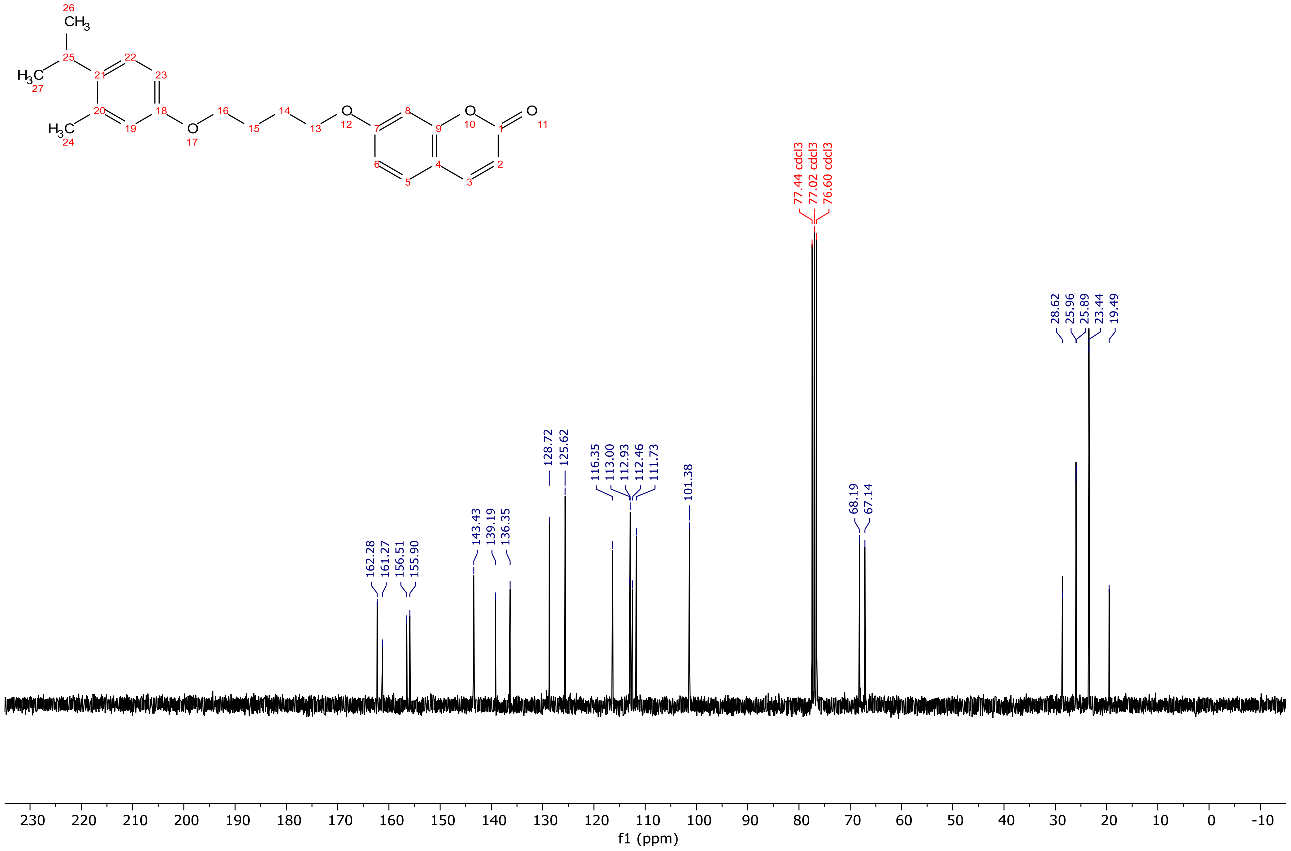

7-((5-(4-isopropyl-3-methylphenoxy)pentyl)oxy)-2H-chromen-2-one (9)

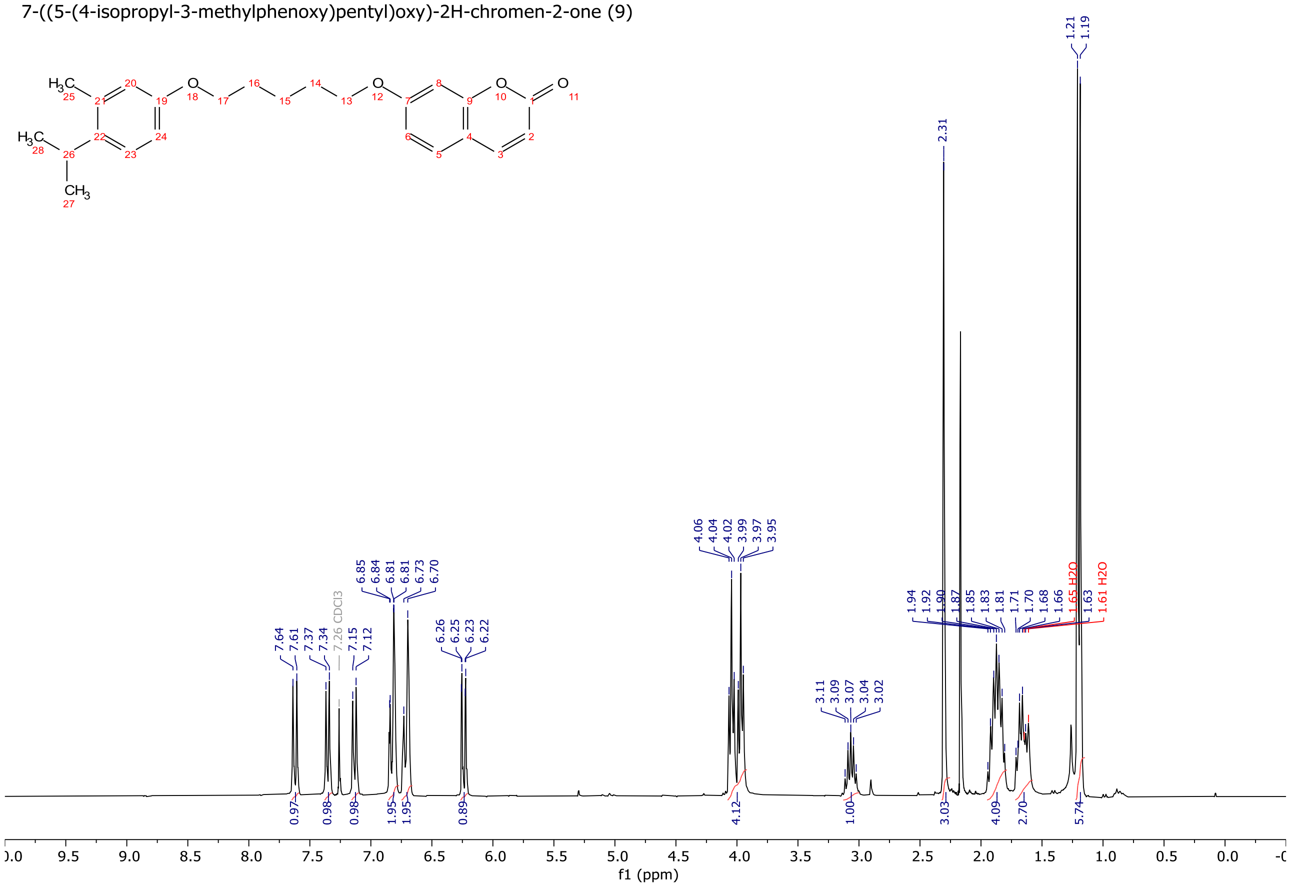

7-((5-(4-isopropyl-3-methylphenoxy)pentyl)oxy)-2H-chromen-2-one (9)

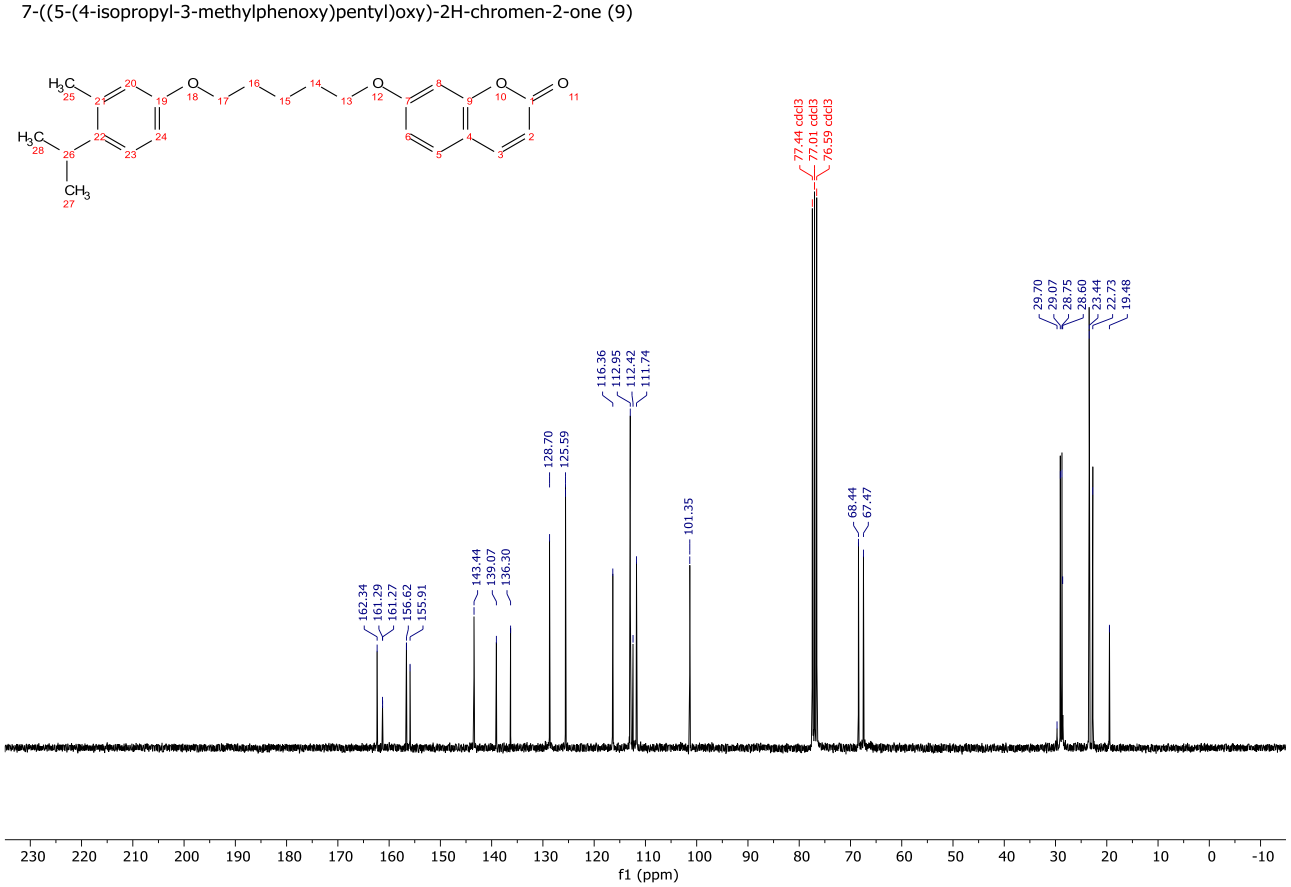

7-((6-(4-chloro-2-isopropyl-5-methylphenoxy)hexyl)oxy)-2H-chromen-2-one (10)

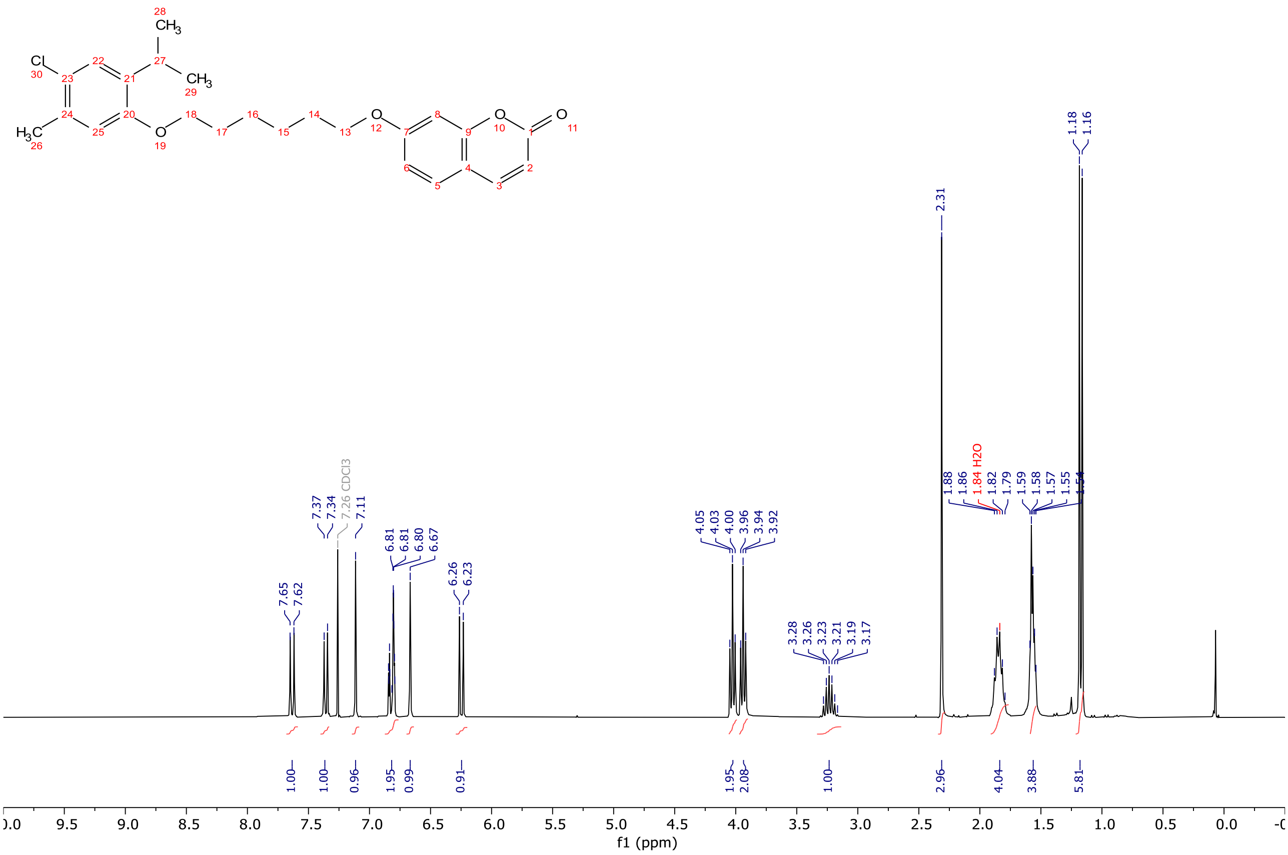

7-((6-(4-chloro-2-isopropyl-5-methylphenoxy)hexyl)oxy)-2H-chromen-2-one (10)

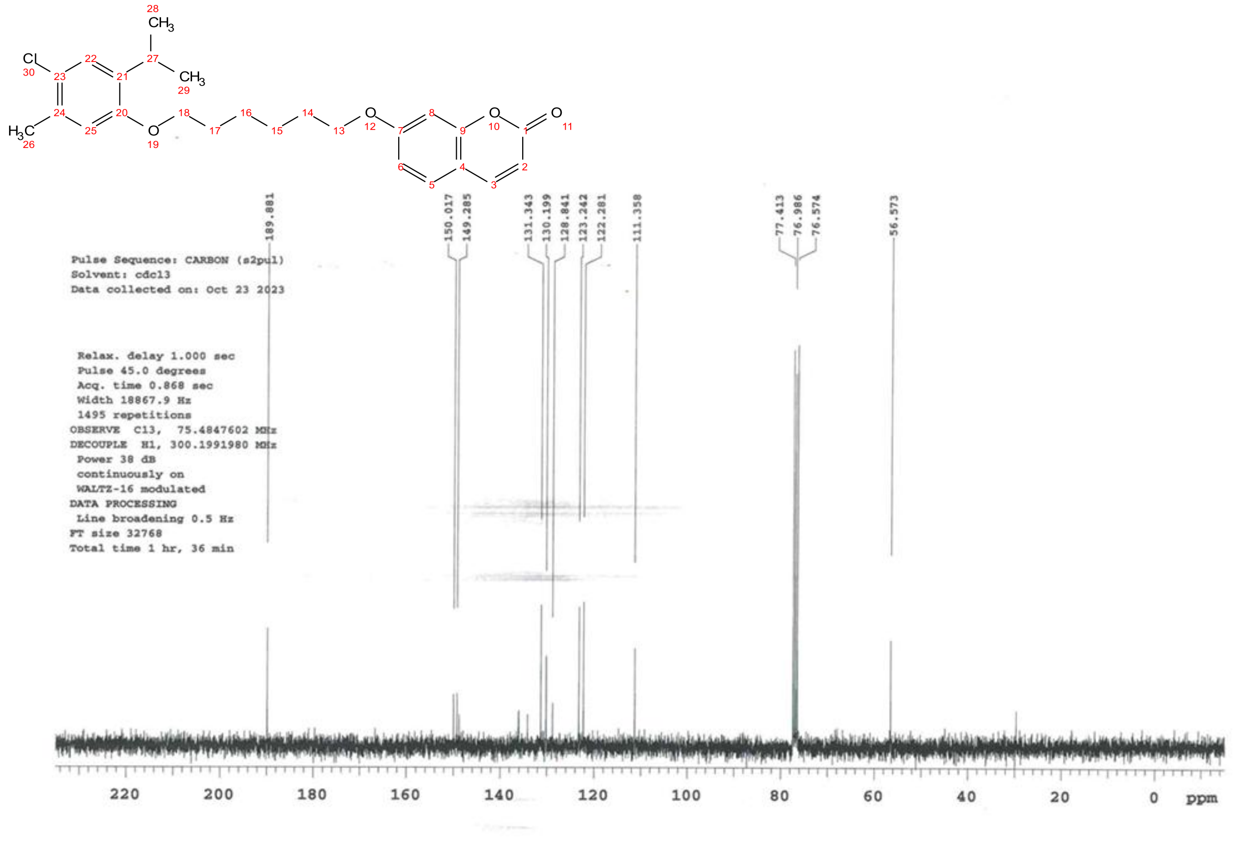

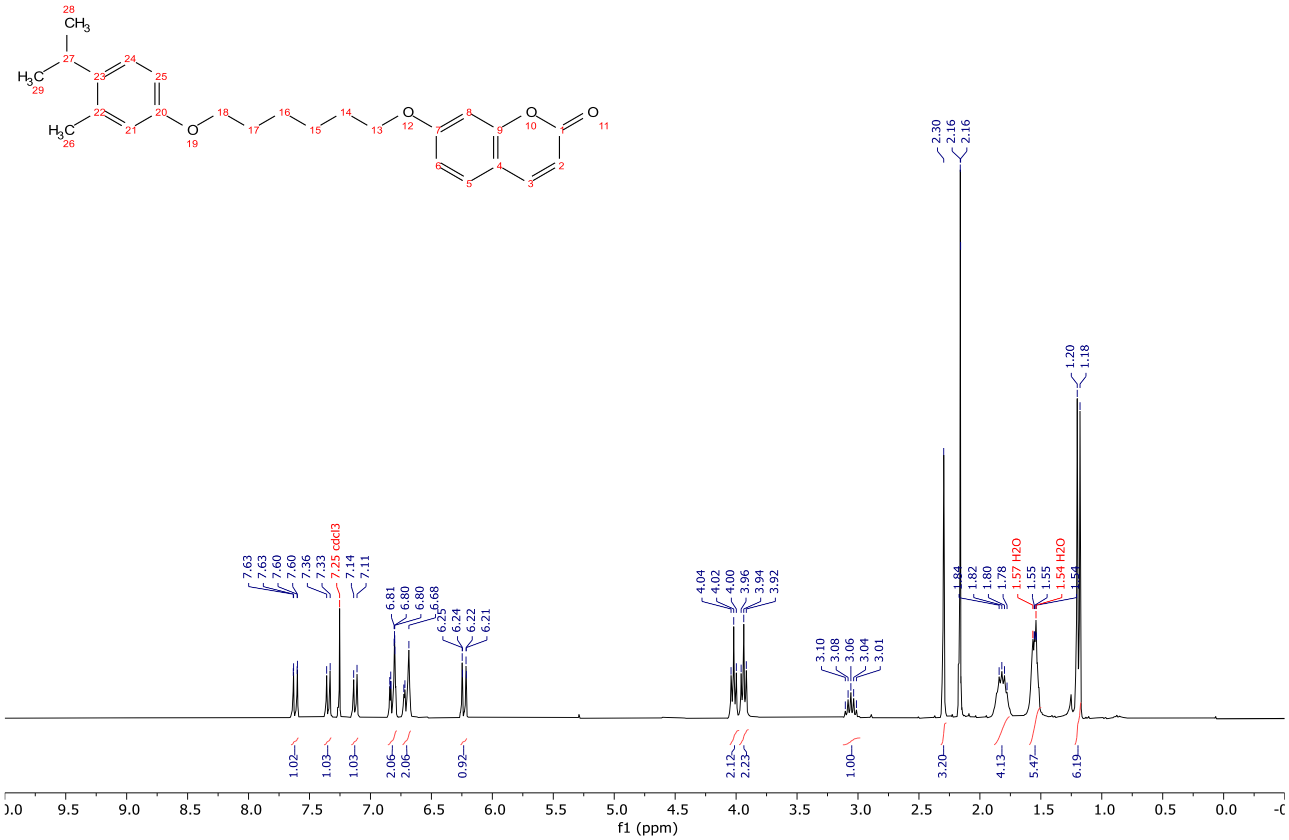

7-((6-(4-isopropyl-3-methylphenoxy)hexyl)oxy)-2H-chromen-2-one (11)

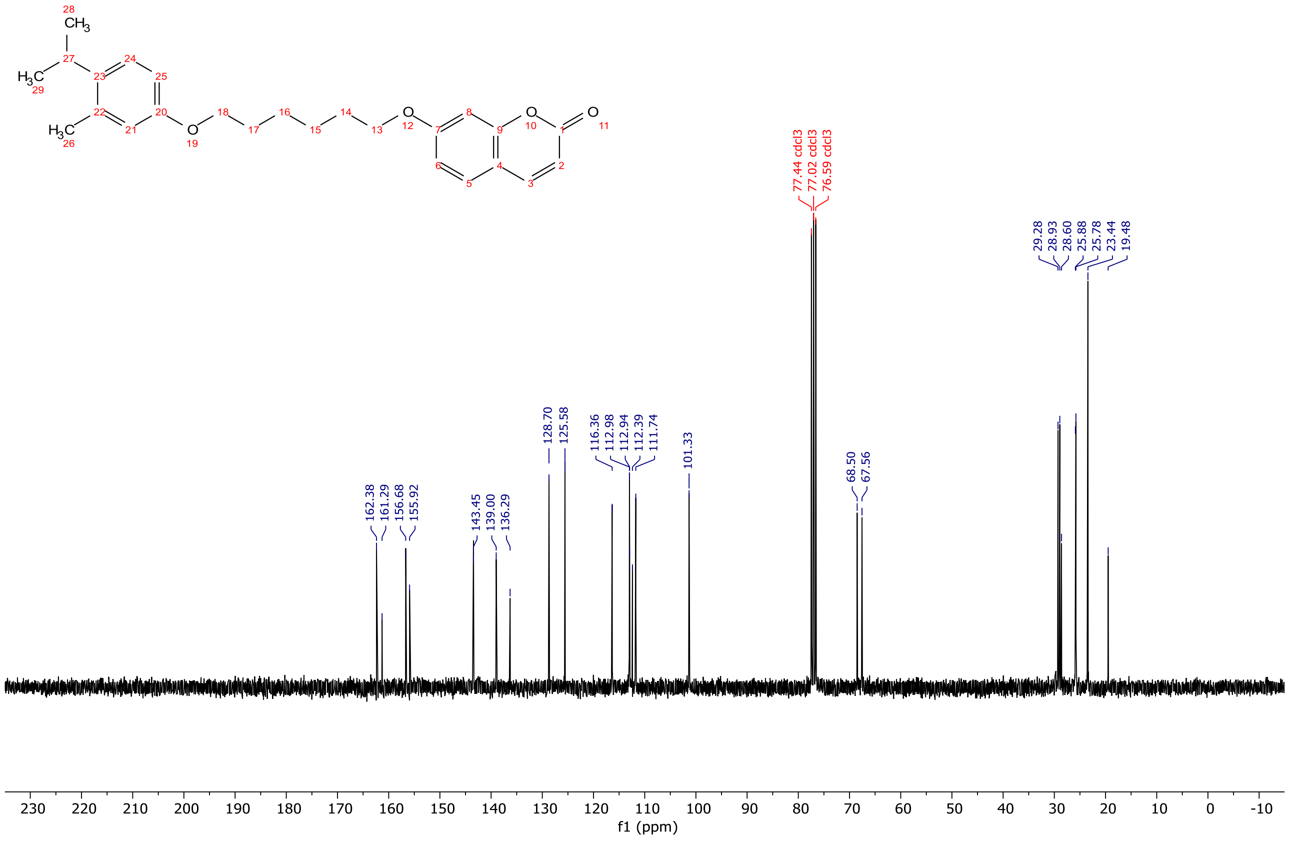

7-((1-(2-(4-chloro-2-isopropyl-5-methylphenoxy)ethyl)-1H-1,2,3-triazol-4-yl)methoxy)-2H-chromen-2-one (13)

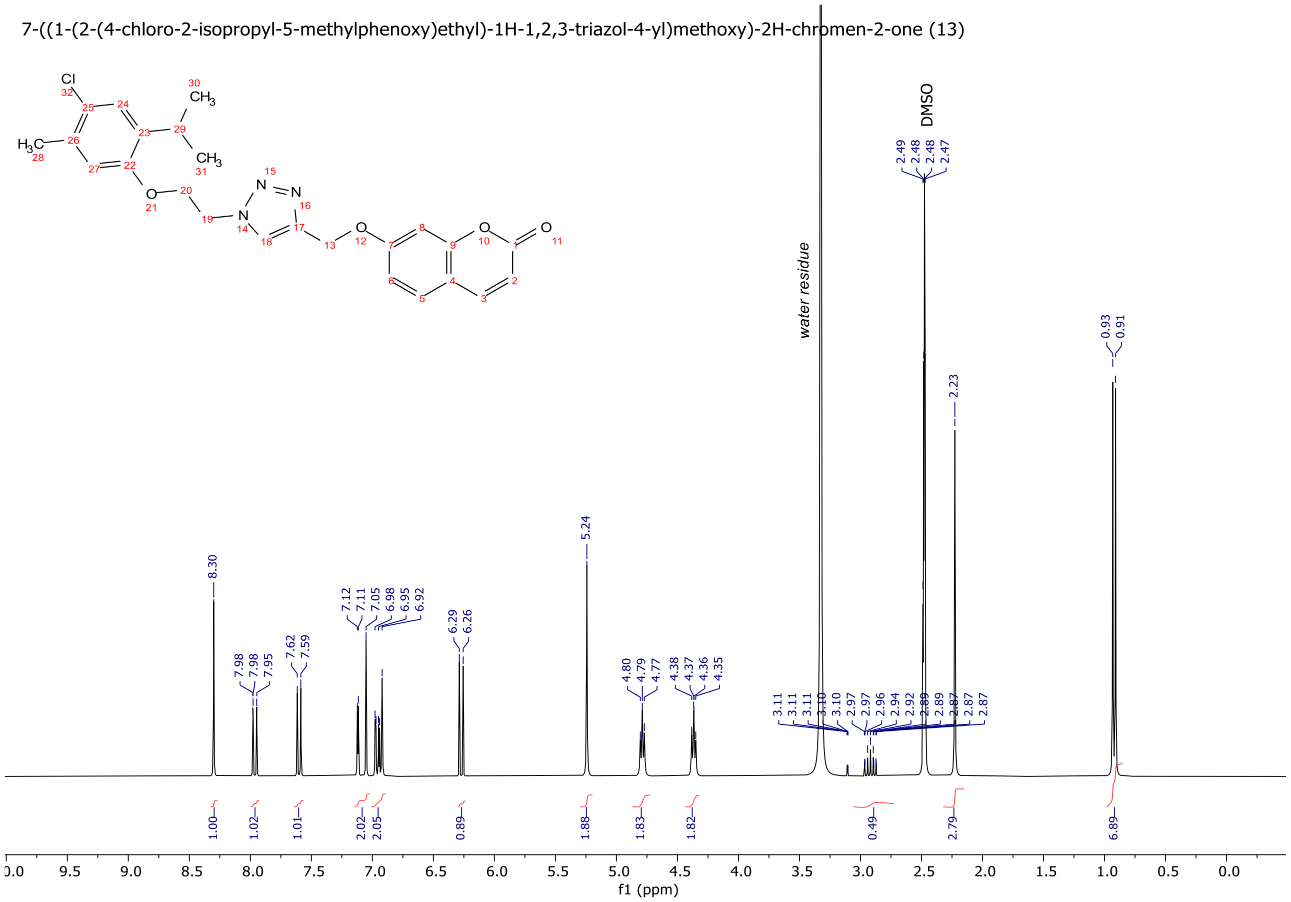

7-((1-(3-(4-chloro-2-isopropyl-5-methylphenoxy)propyl)-1H-1,2,3-triazol-4-yl)methoxy)-2H-chromen-2-one (14).

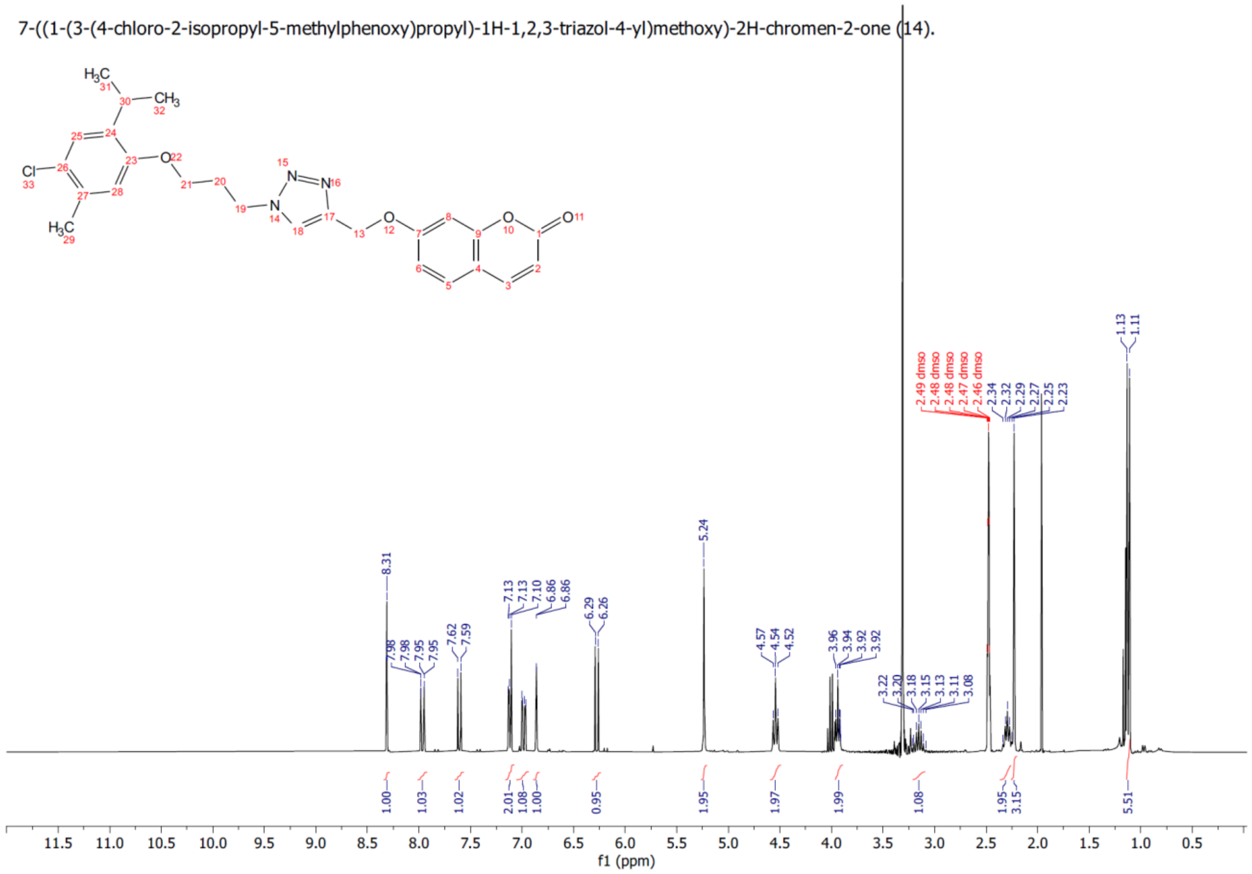

7-((1-(3-(4-chloro-2-isopropyl-5-methylphenoxy)propyl)-1H-1,2,3-triazol-4-yl)methoxy)-2H-chromen-2-one (14)

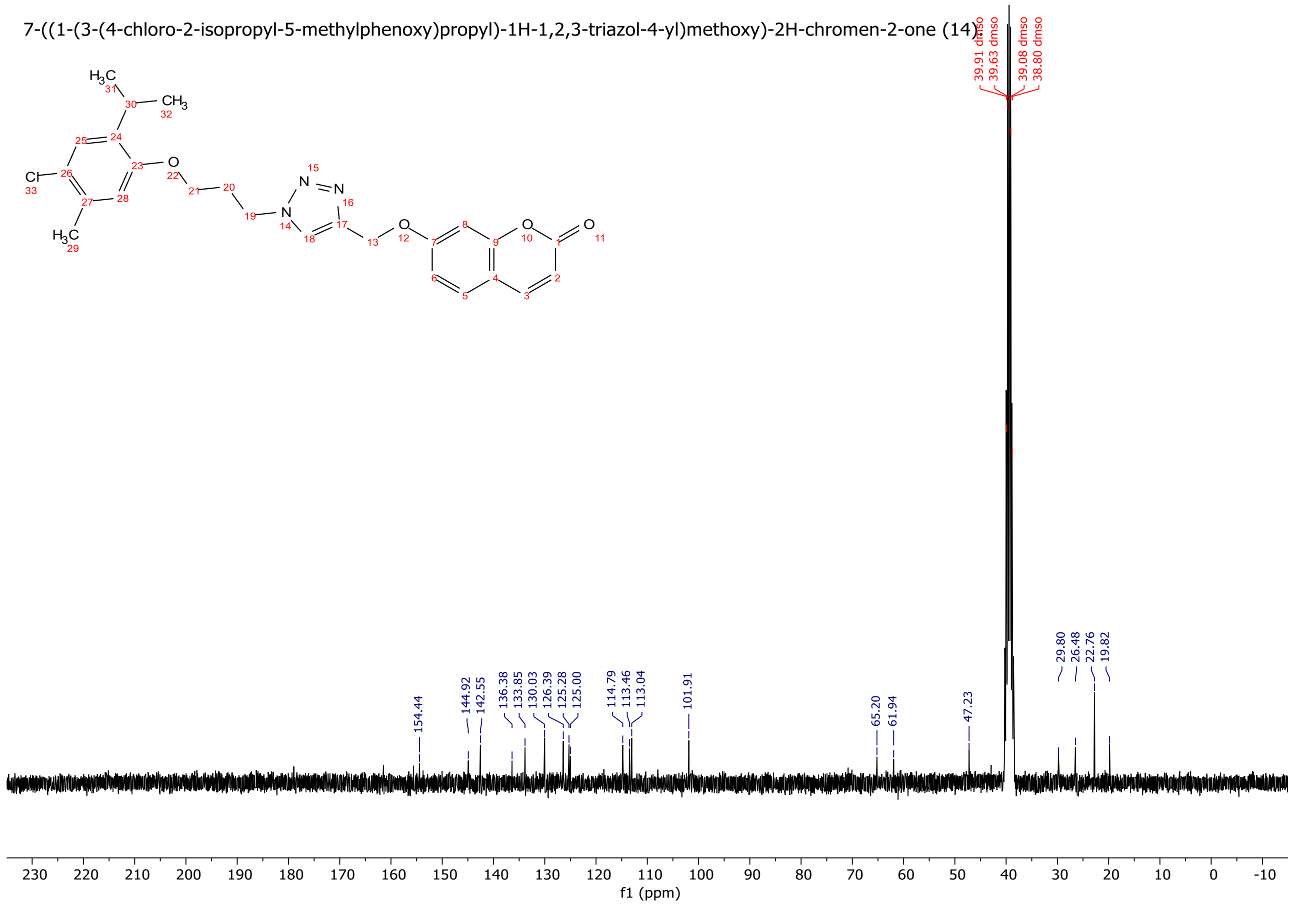

7-((1-(3-(4-isopropyl-3-methylphenoxy)propyl)-1H-1,2,3-triazol-4-yl)methoxy)-2H-chromen-2-one (15)

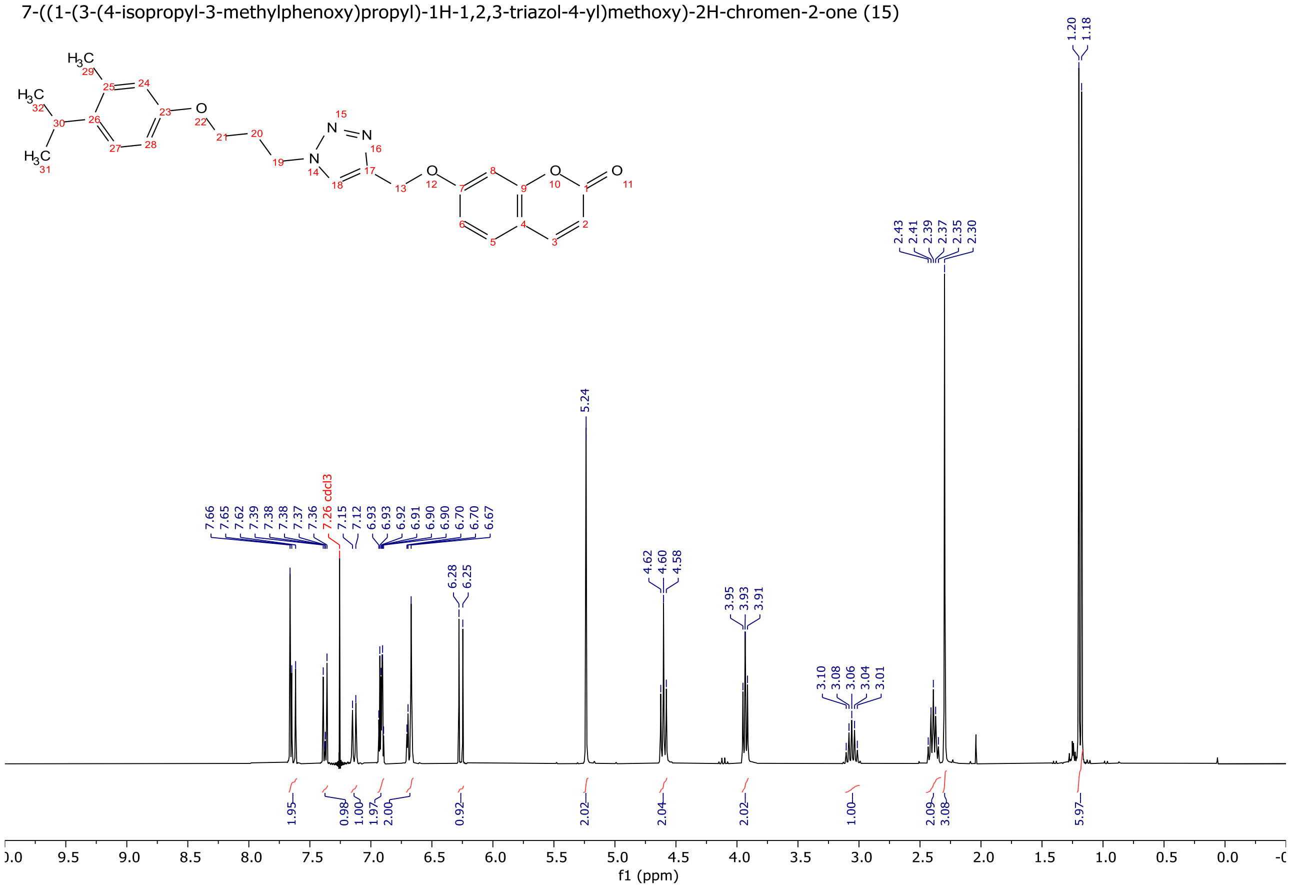

7-((1-(3-(4-isopropyl-3-methylphenoxy)propyl)-1H-1,2,3-triazol-4-yl)methoxy)-2H-chromen-2-one (15)

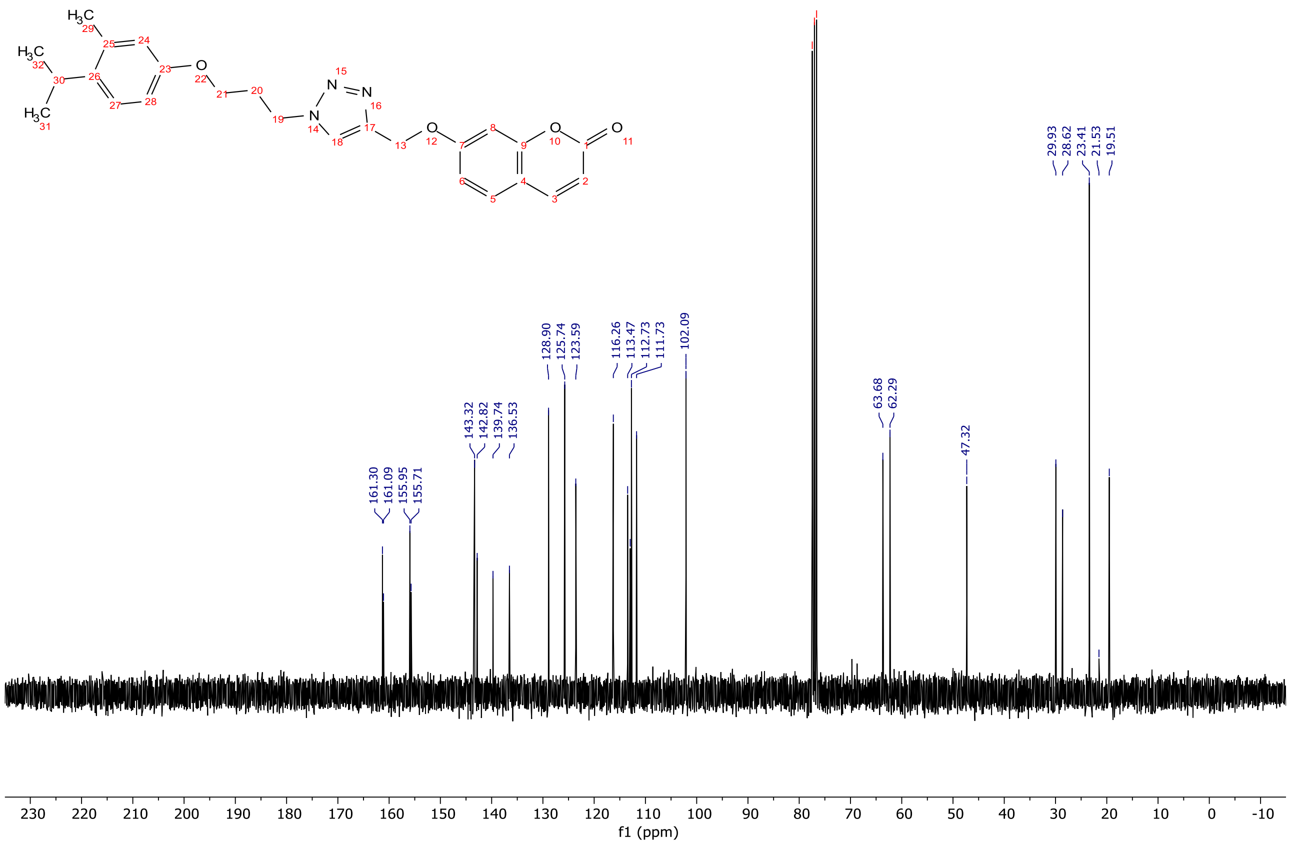

7-((1-(4-(4-chloro-2-isopropyl-5-methylphenoxy)butyl)-1H-1,2,3-triazol-4-yl)methoxy)-2H-chromen-2-one (16).

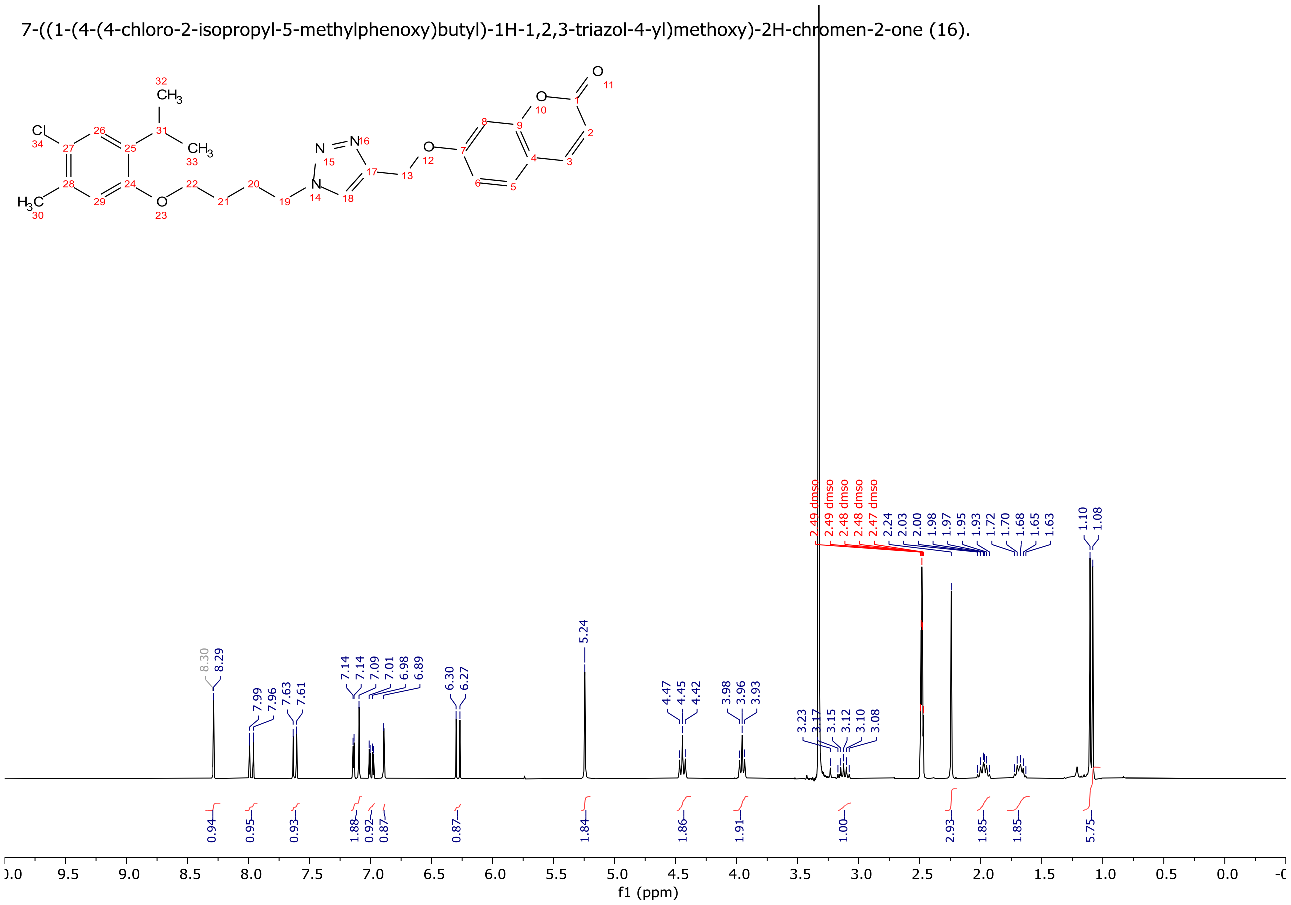

7-((1-(4-(4-chloro-2-isopropyl-5-methylphenoxy)butyl)-1H-1,2,3-triazol-4-yl)methoxy)-2H-chromen-2-one (16).

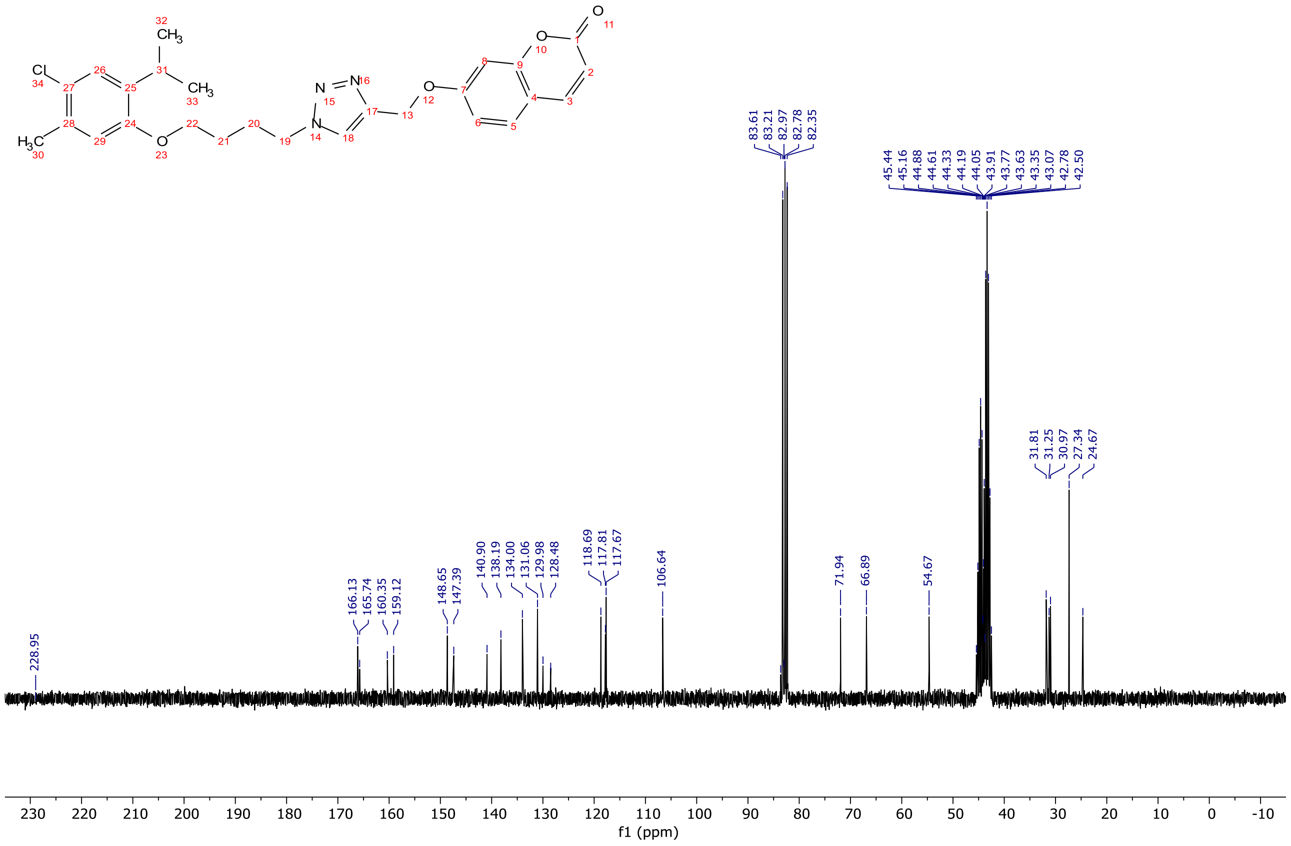

7-((1-(4-(4-isopropyl-3-methylphenoxy)butyl)-1H-1,2,3-triazol-4-yl)methoxy)-2H-chromen-2-one (17).

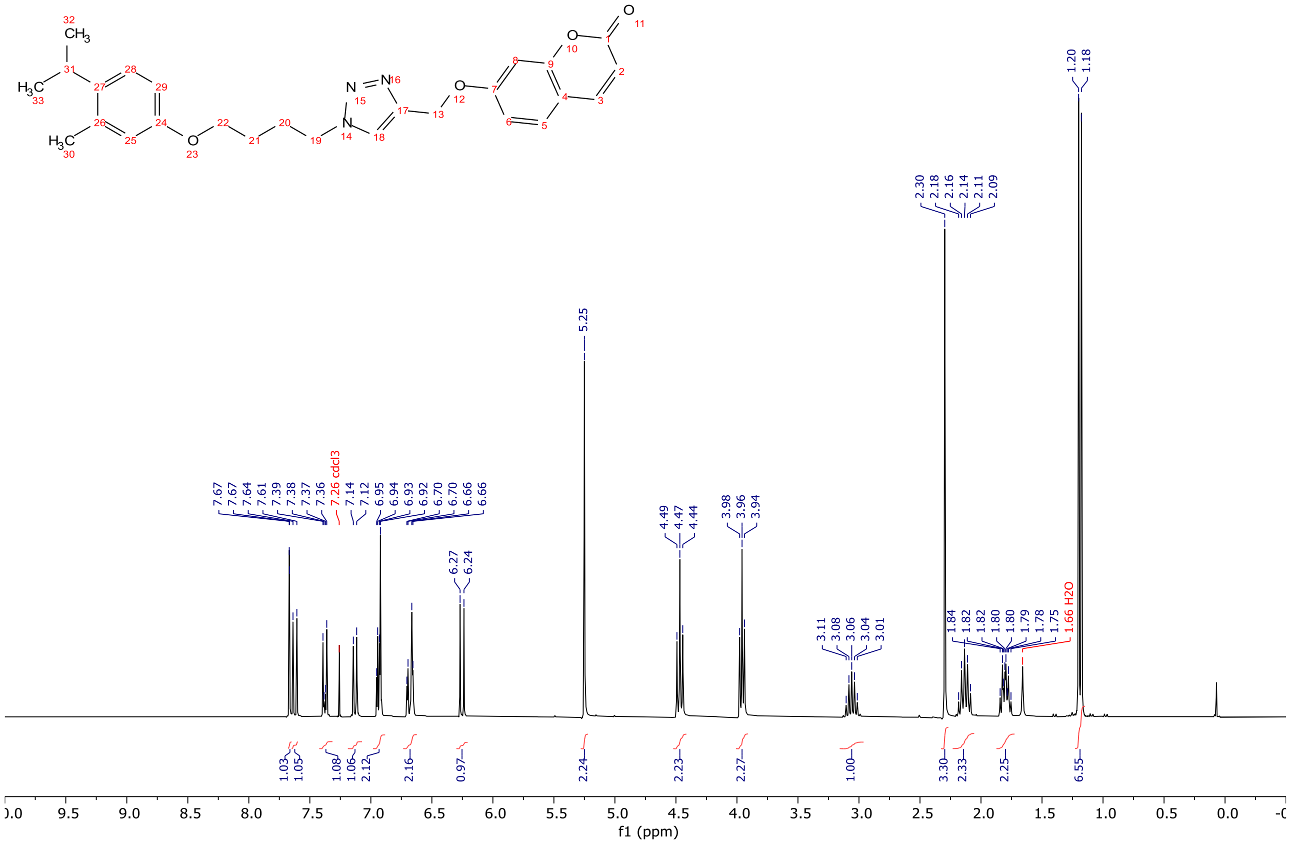

7-((1-(4-(4-isopropyl-3-methylphenoxy)butyl)-1H-1,2,3-triazol-4-yl)methoxy)-2H-chromen-2-one (17).

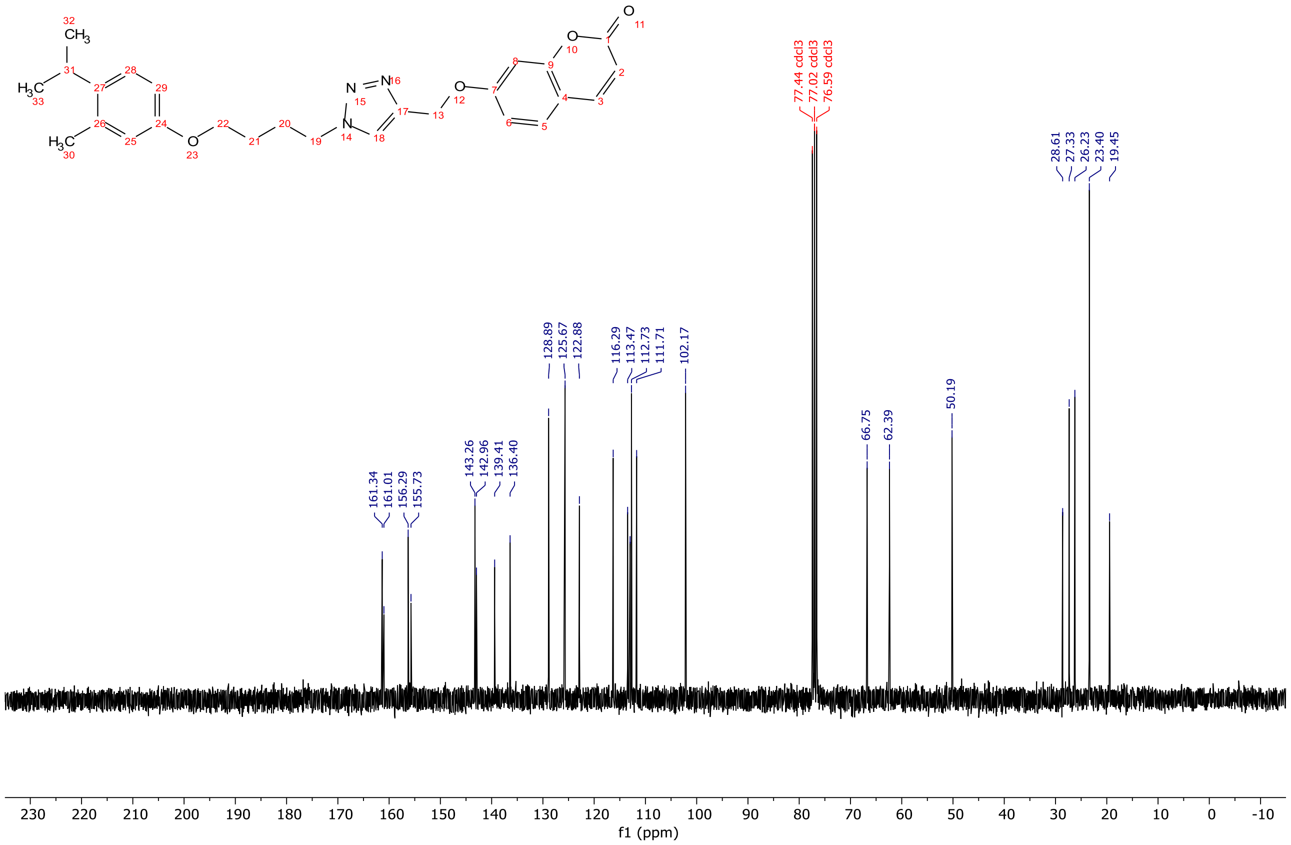

7-((1-(5-(4-chloro-2-isopropyl-5-methylphenoxy)pentyl)-1H-1,2,3-triazol-4-yl)methoxy)-2H-chromen-2-one (18).

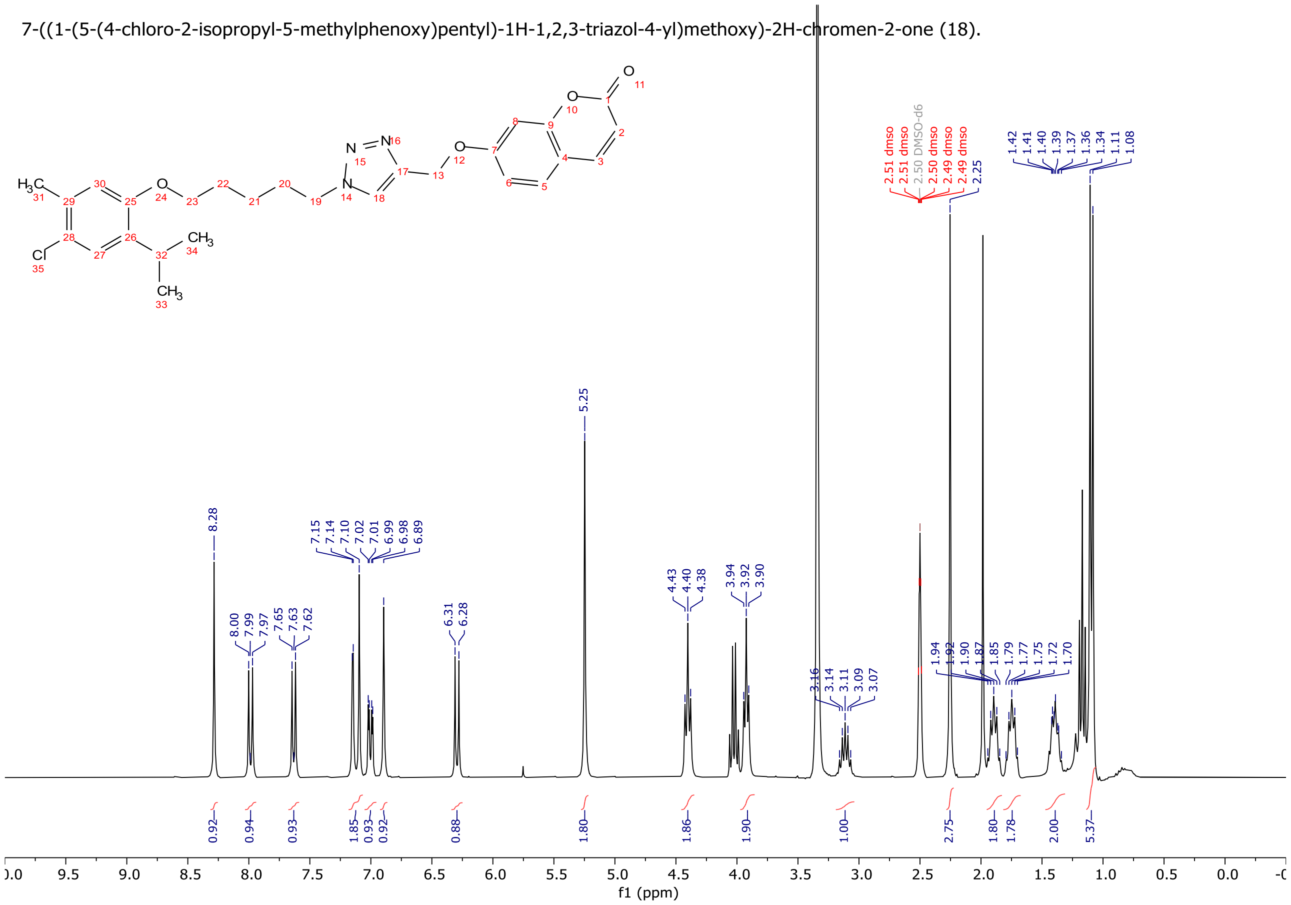

7-((1-(5-(4-chloro-2-isopropyl-5-methylphenoxy)pentyl)-1H-1,2,3-triazol-4-yl)methoxy)-2H-chromen-2-one (18).

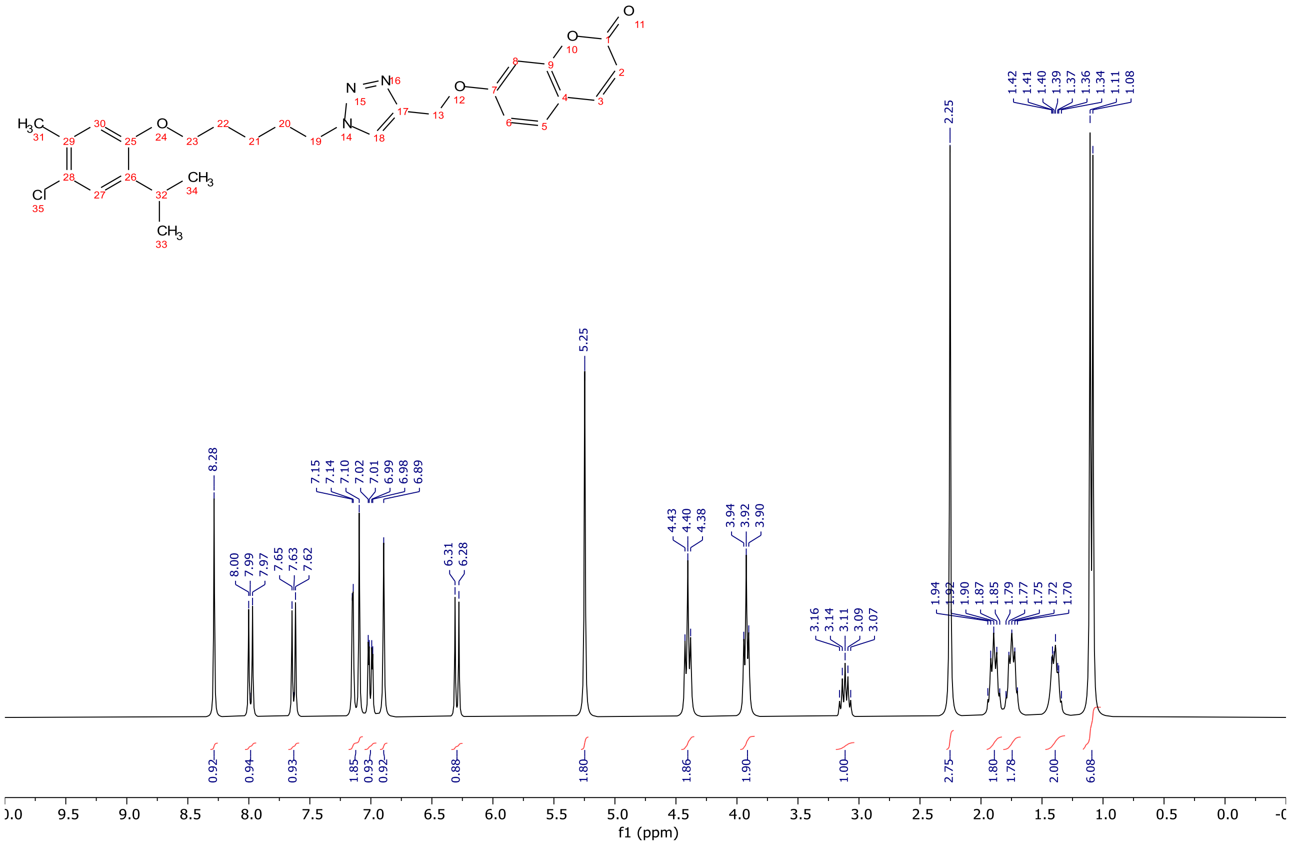

7-((1-(5-(4-chloro-2-isopropyl-5-methylphenoxy)pentyl)-1H-1,2,3-triazol-4-yl)methoxy)-2H-chromen-2-one (18).

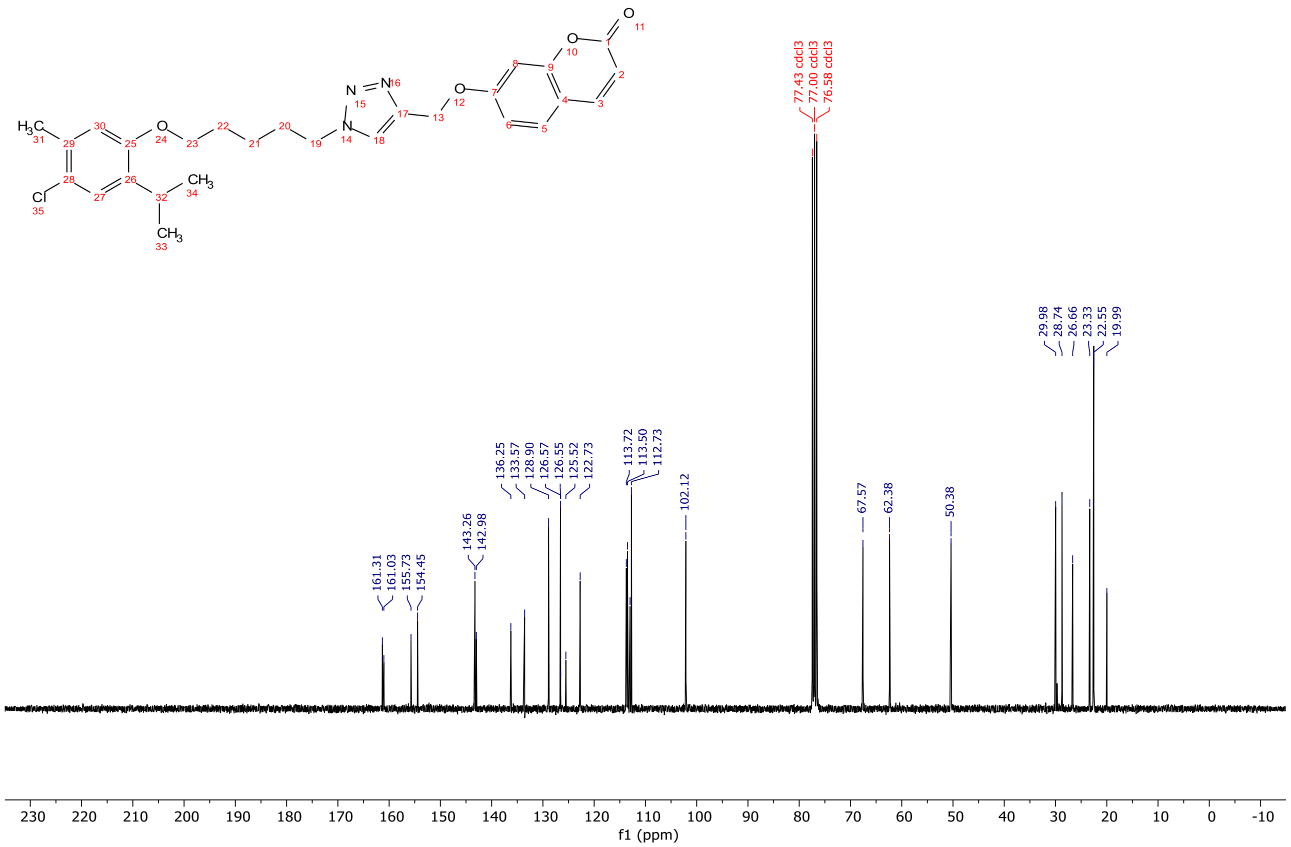

7-((1-(5-(4-isopropyl-3-methylphenoxy)pentyl)-1H-1,2,3-triazol-4-yl)methoxy)-2H-chromen-2-one (19).

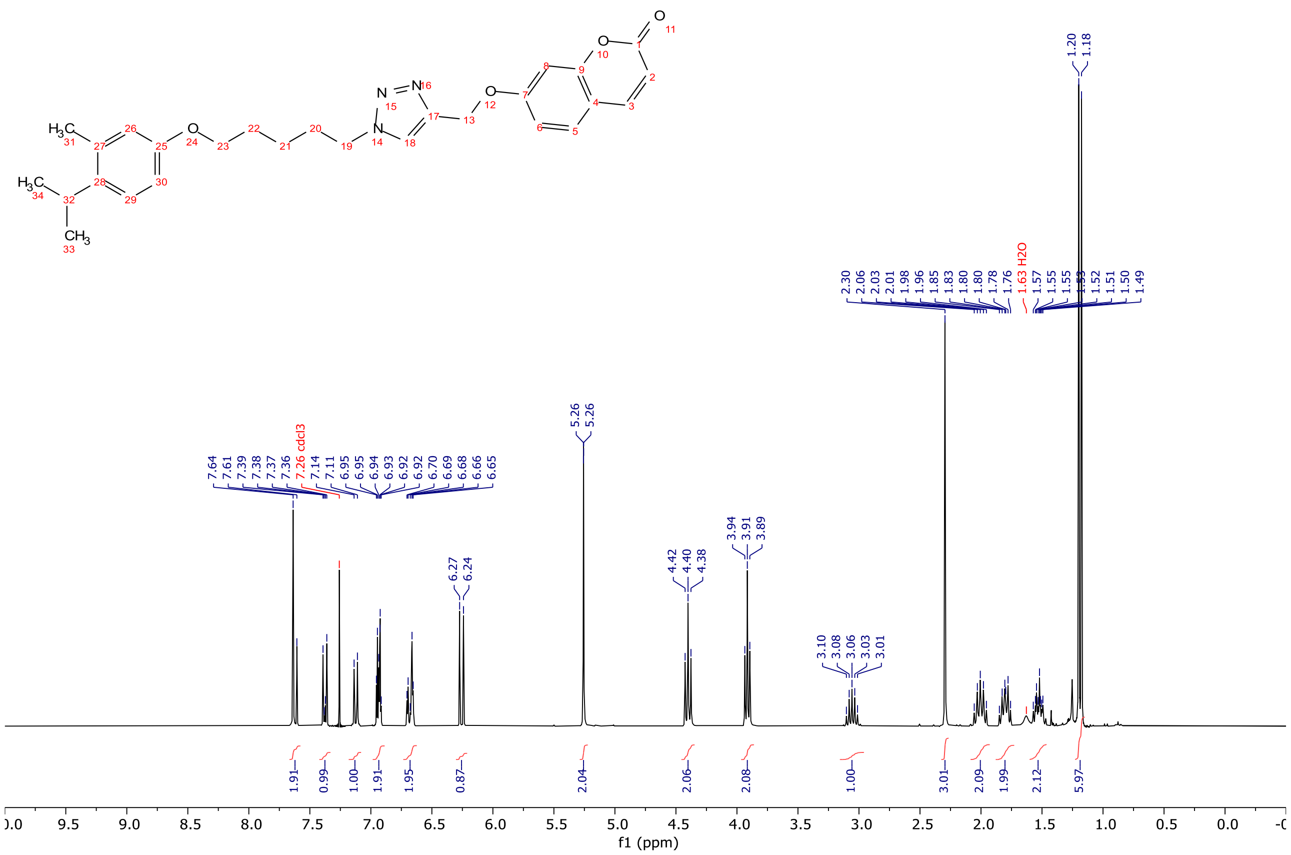

7-((1-(5-(4-isopropyl-3-methylphenoxy)pentyl)-1H-1,2,3-triazol-4-yl)methoxy)-2H-chromen-2-one (19).

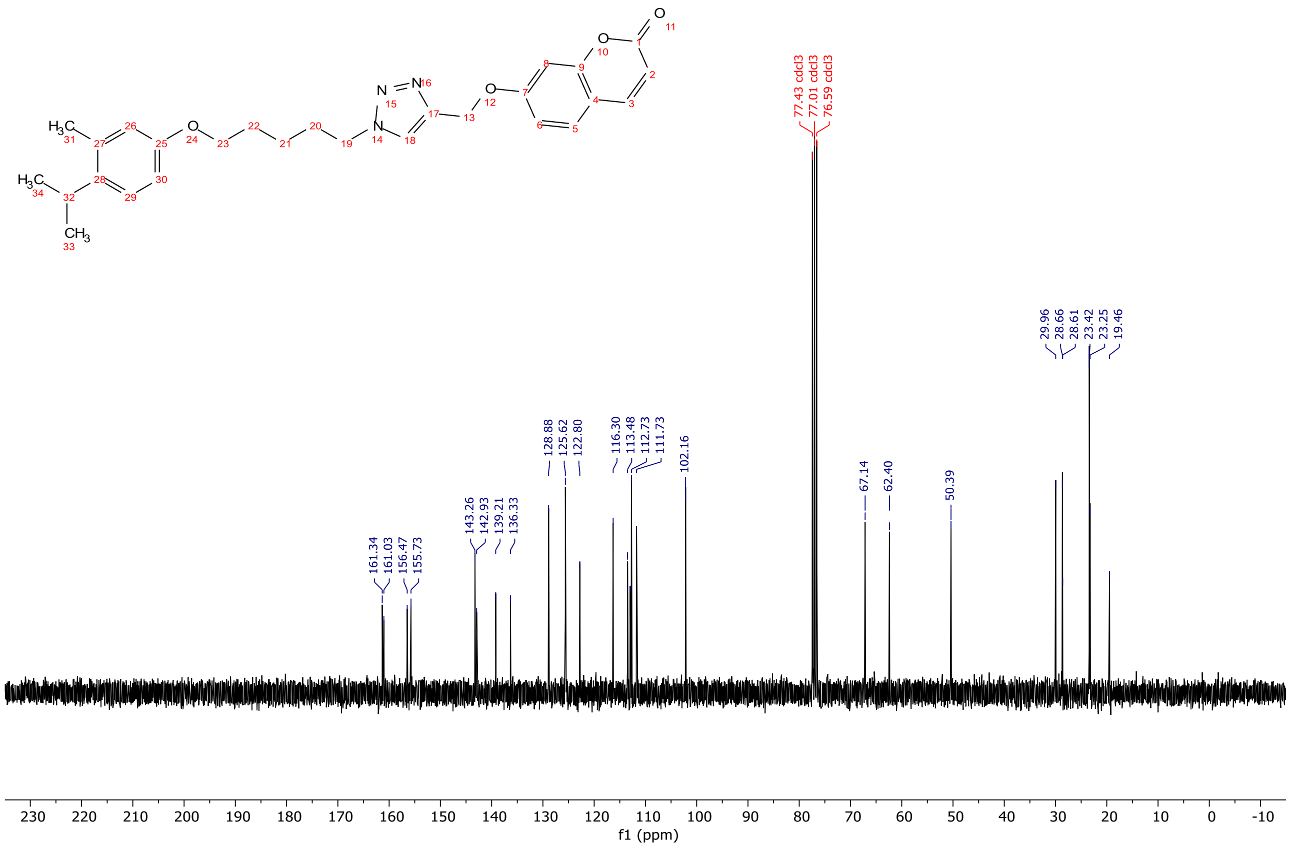

7-((1-(6-(4-chloro-2-isopropyl-5-methylphenoxy)hexyl)-1H-1,2,3-triazol-4-yl)methoxy)-2H-chromen-2-one (20).

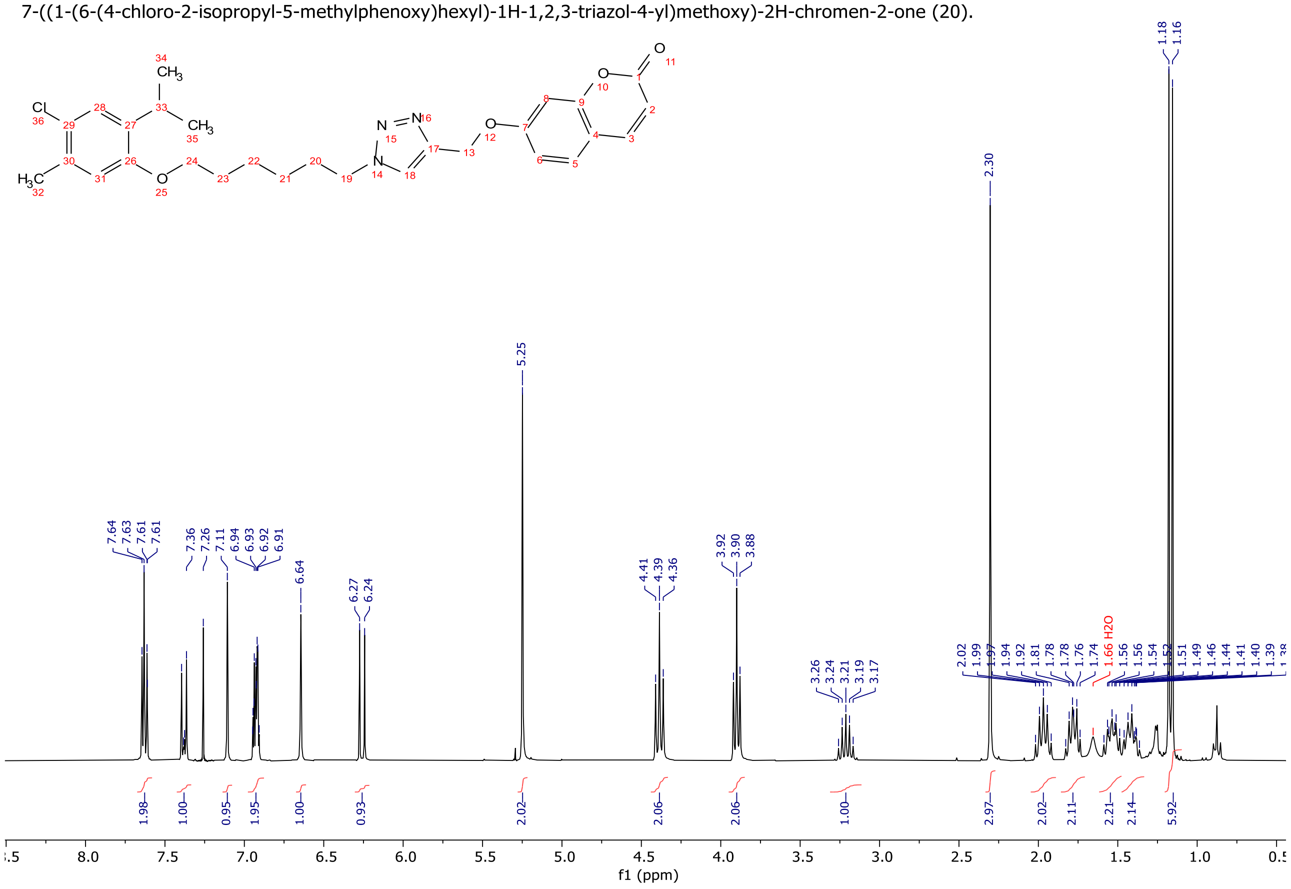

7-((1-(6-(4-chloro-2-isopropyl-5-methylphenoxy)hexyl)-1H-1,2,3-triazol-4-yl)methoxy)-2H-chromen-2-one (20).

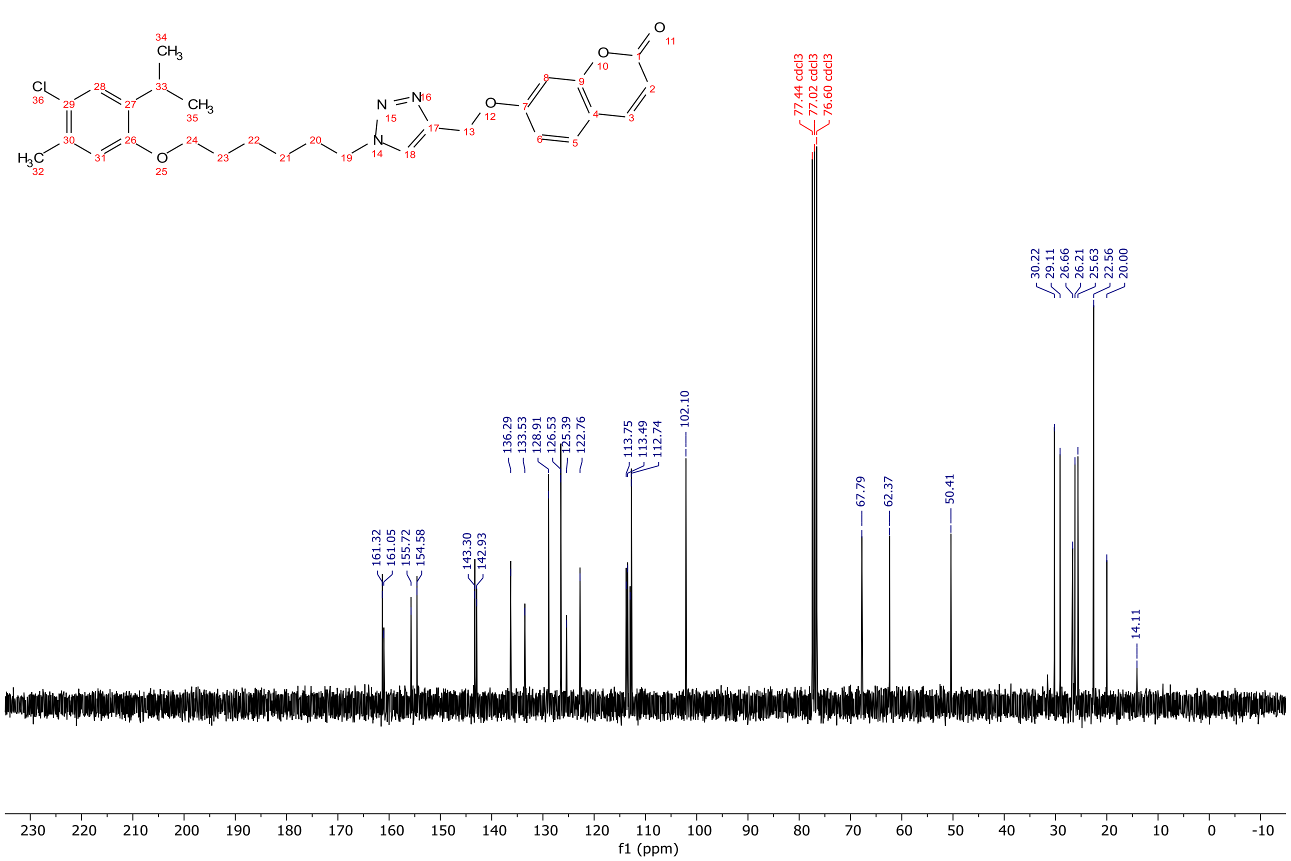

7-((1-(6-(4-isopropyl-3-methylphenoxy)hexyl)-1H-1,2,3-triazol-4-yl)methoxy)-2H-chromen-2-one (21).

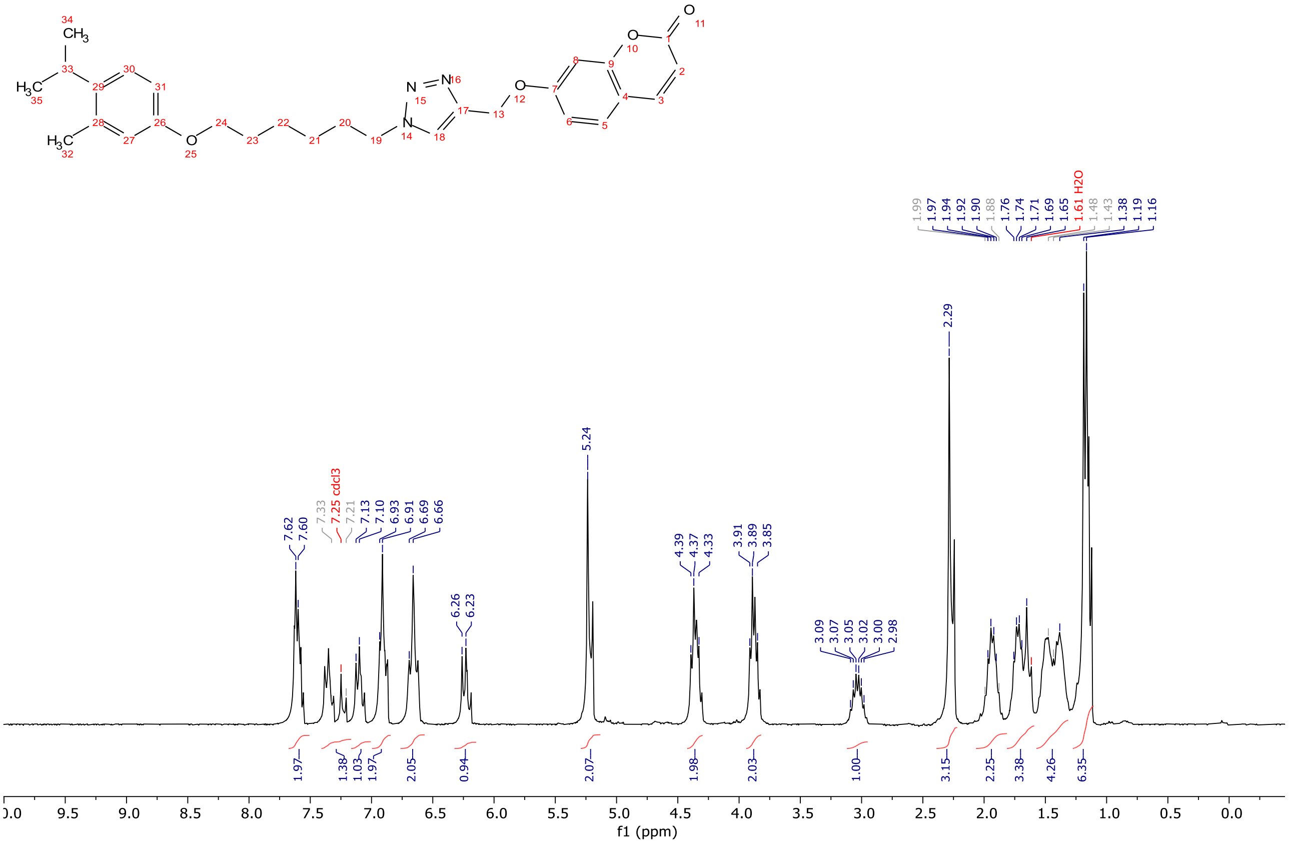

7-((1-(6-(4-isopropyl-3-methylphenoxy)hexyl)-1H-1,2,3-triazol-4-yl)methoxy)-2H-chromen-2-one (21).

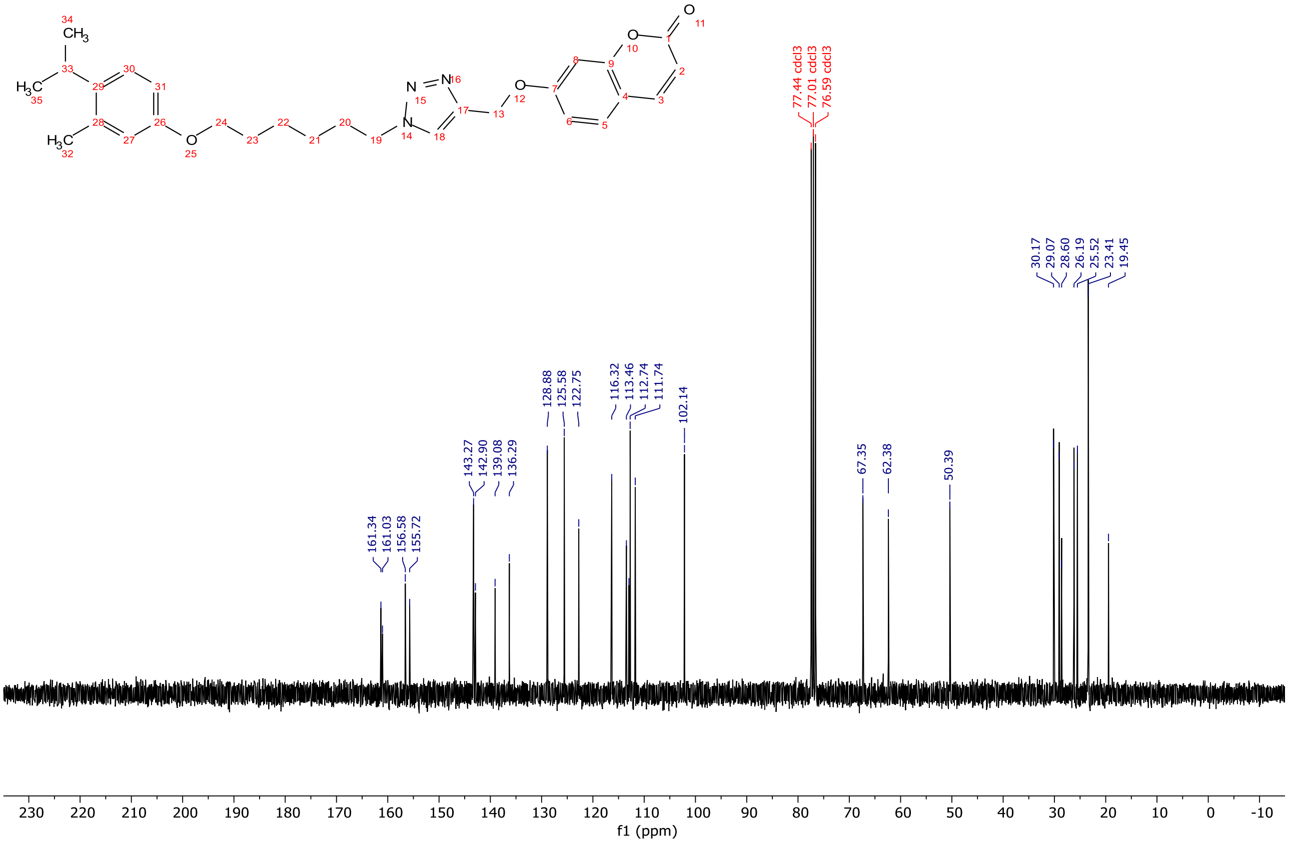

2-(4-isopropyl-3-methylphenoxy)-N,N-bis(pyridin-2-ylmethyl)ethan-1-amine (23)

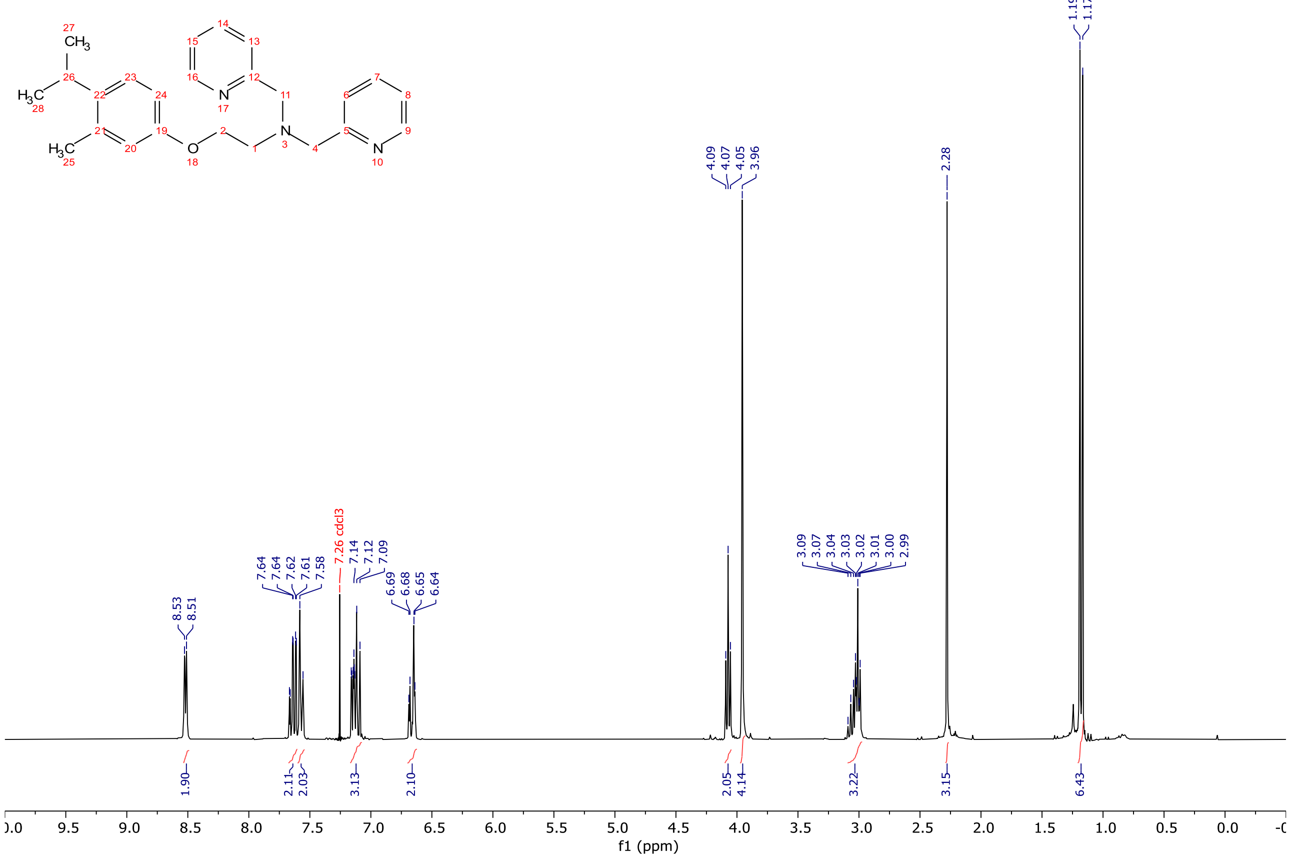

2-(4-isopropyl-3-methylphenoxy)-N,N-bis(pyridin-2-ylmethyl)ethan-1-amine (23)

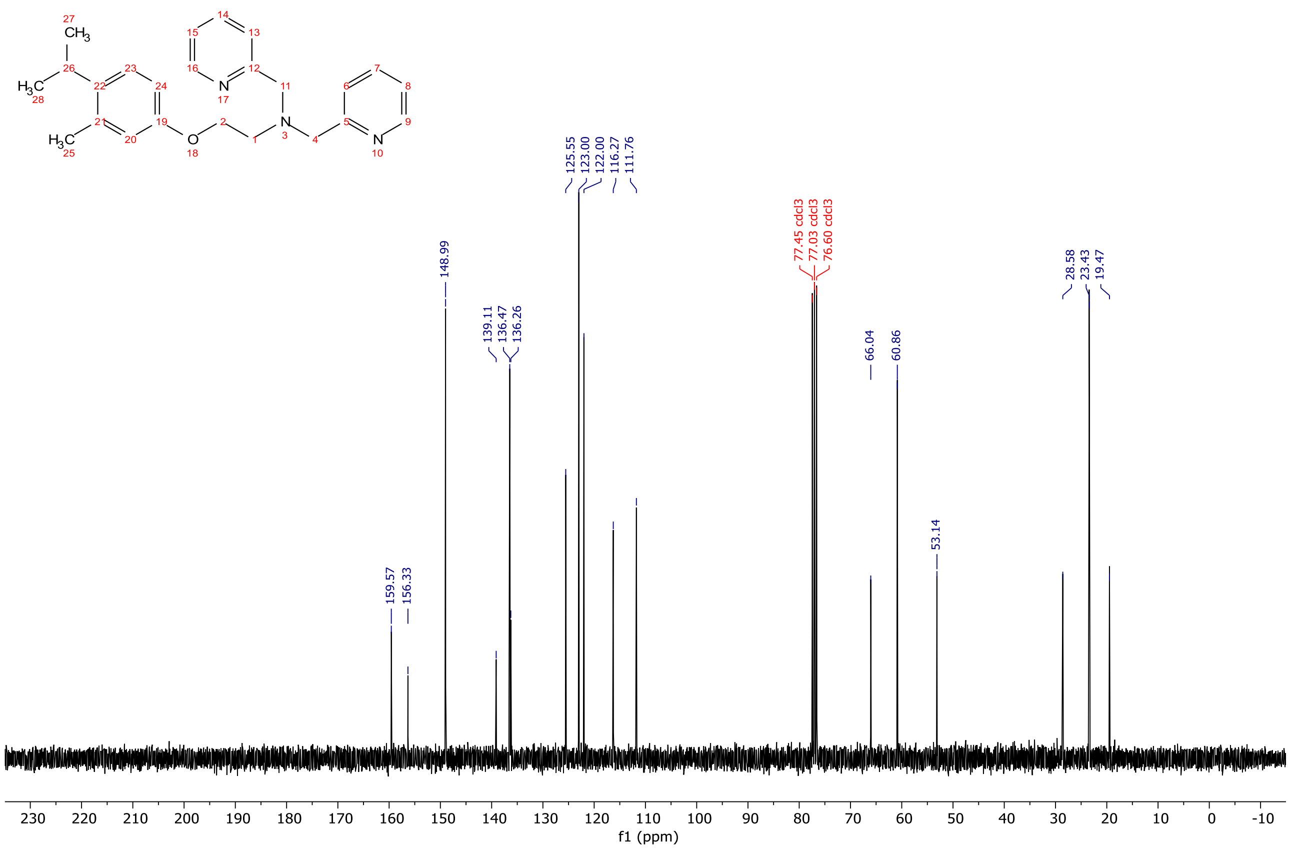

3-(4-isopropyl-3-methylphenoxy)-N,N-bis(pyridin-2-ylmethyl)propan-1-amine (24).

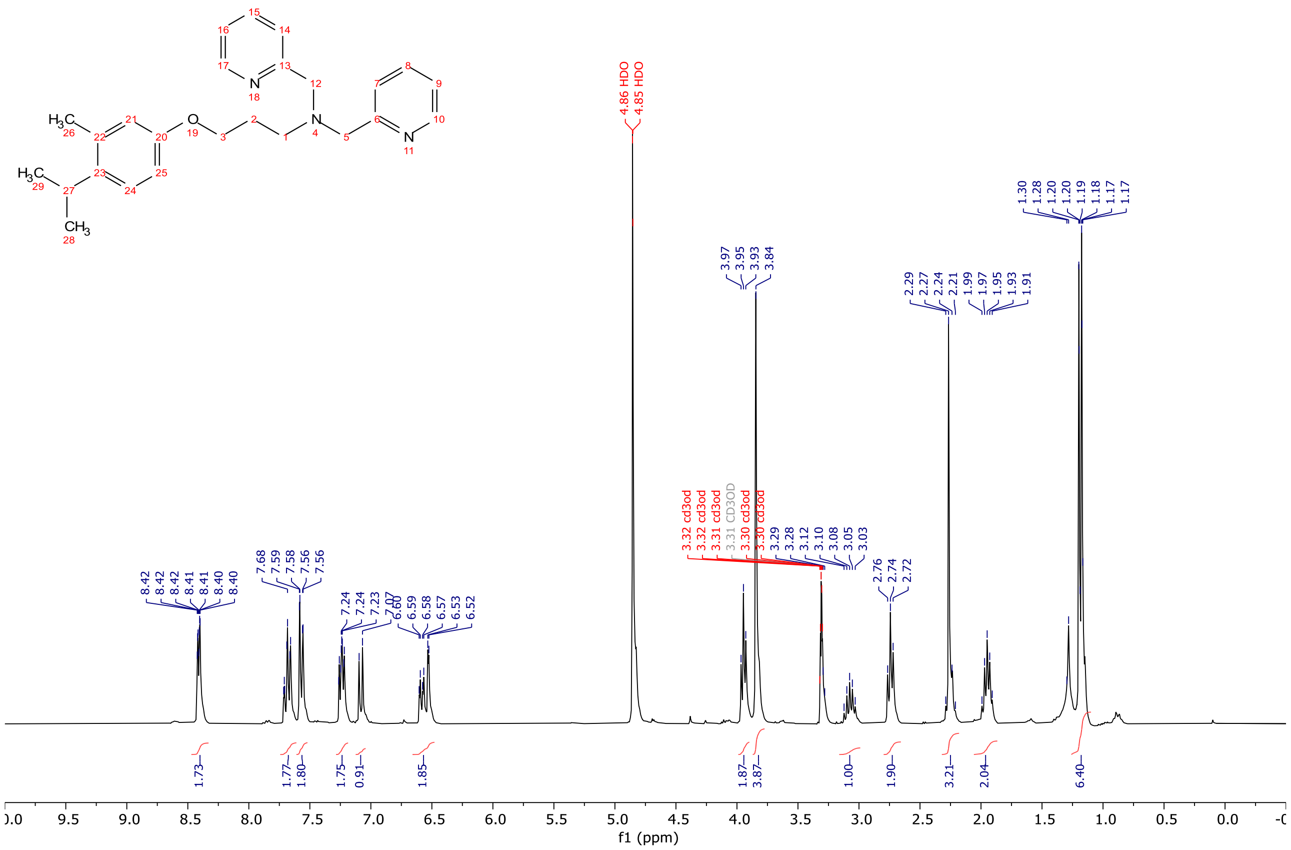

3-(4-isopropyl-3-methylphenoxy)-N,N-bis(pyridin-2-ylmethyl)propan-1-am

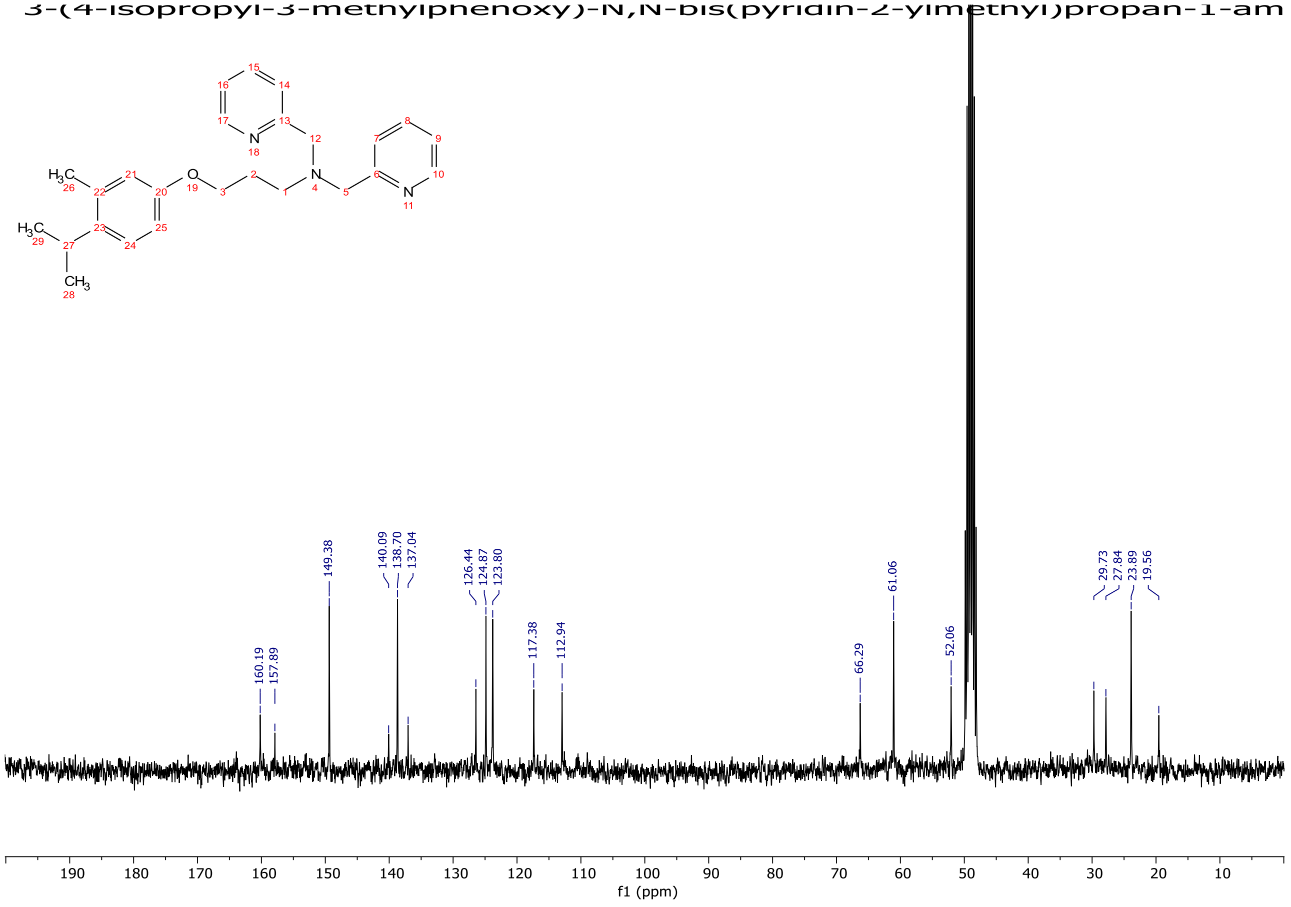

4-(4-chloro-2-isopropyl-5-methylphenoxy)-N,N-bis(pyridin-2-ylmethyl)butan-1-amine (25).

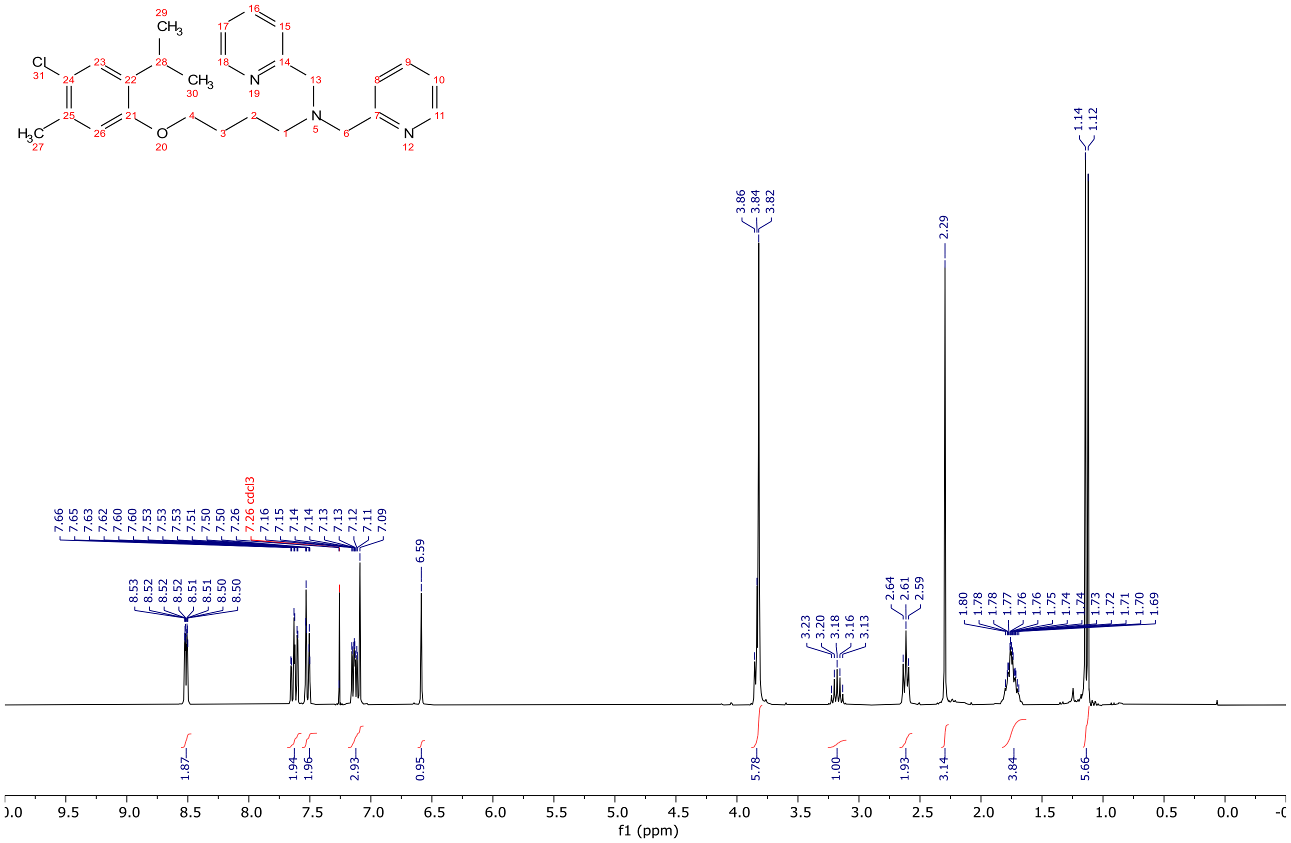

4-(4-chloro-2-isopropyl-5-methylphenoxy)-N,N-bis(pyridin-2-ylmethyl)butan-1-amine (25).

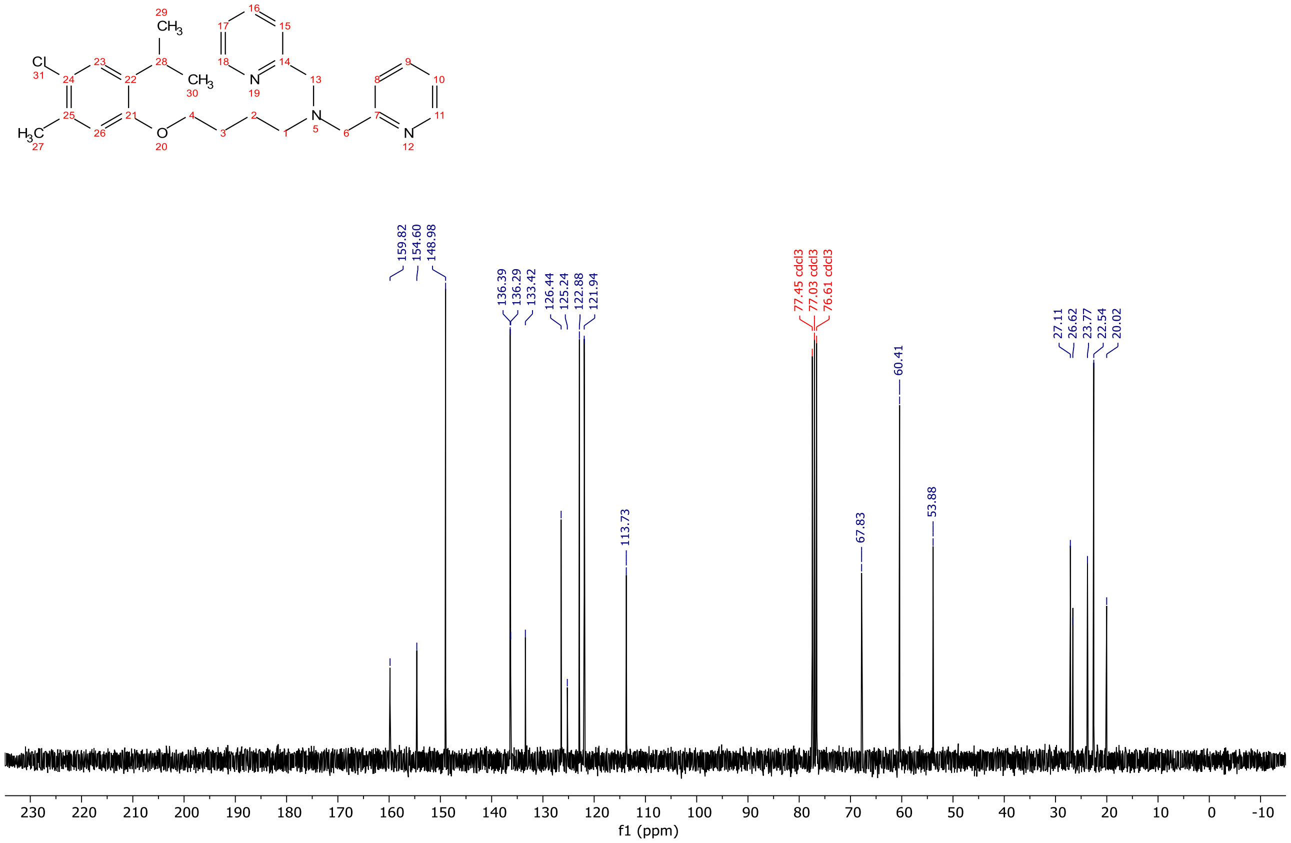

4-(4-isopropyl-3-methylphenoxy)-N,N-bis(pyridin-2-ylmethyl)butan-1-amine (26).

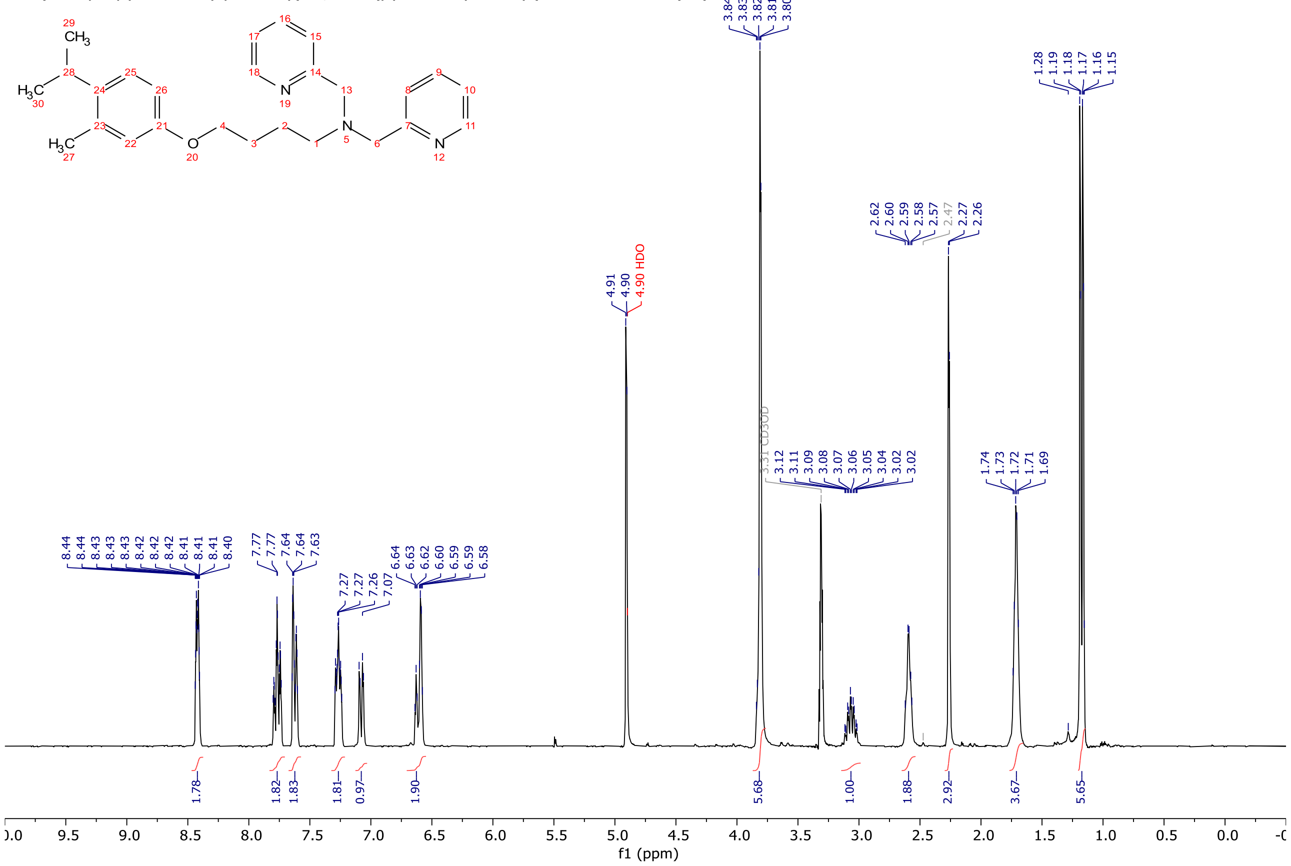

4-(4-isopropyl-3-methylphenoxy)-N,N-bis(pyridin-2-ylmethyl)butan-1-amine (26).

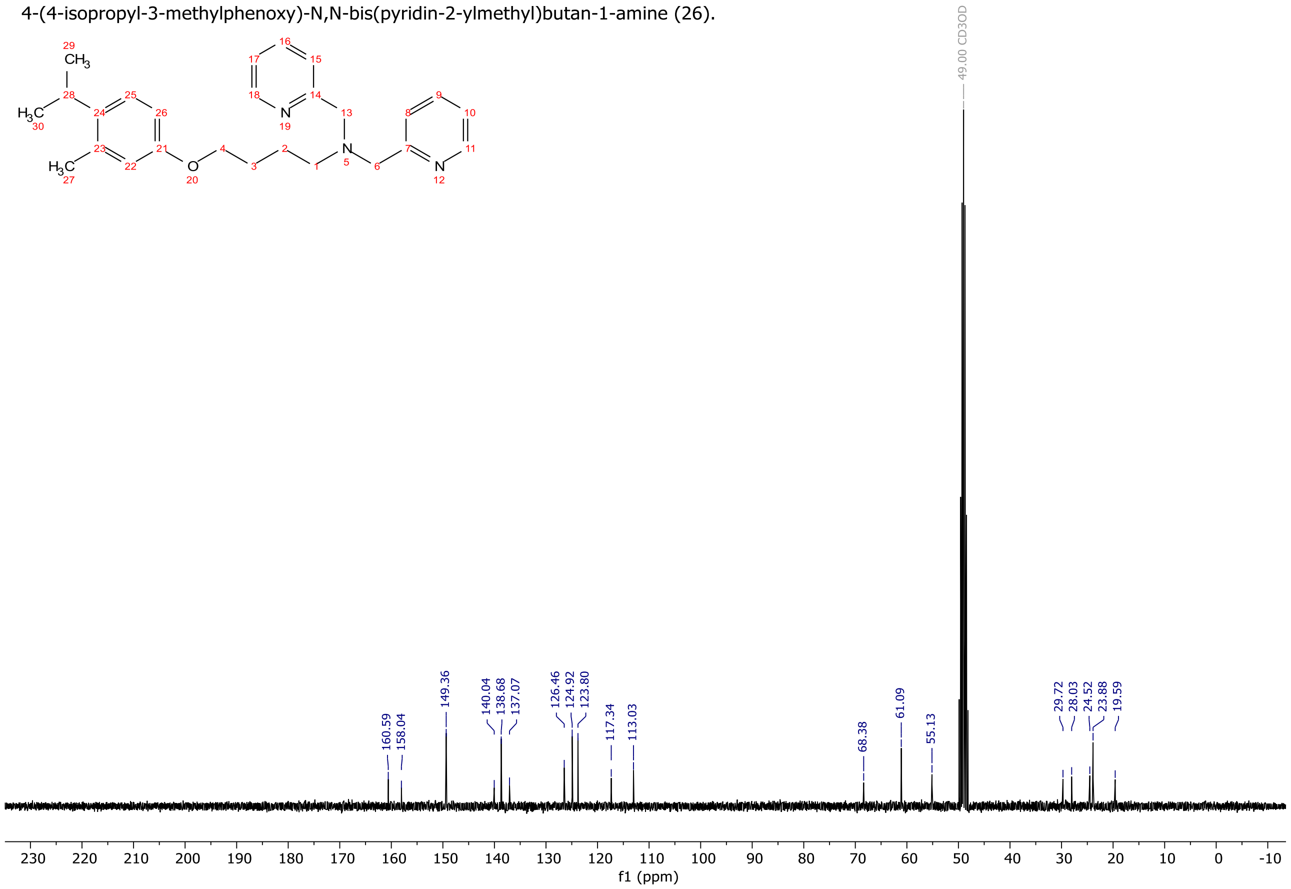

5-(4-chloro-2-isopropyl-5-methylphenoxy)-N,N-bis(pyridin-2-ylmethyl)pentan-1-amine (27).

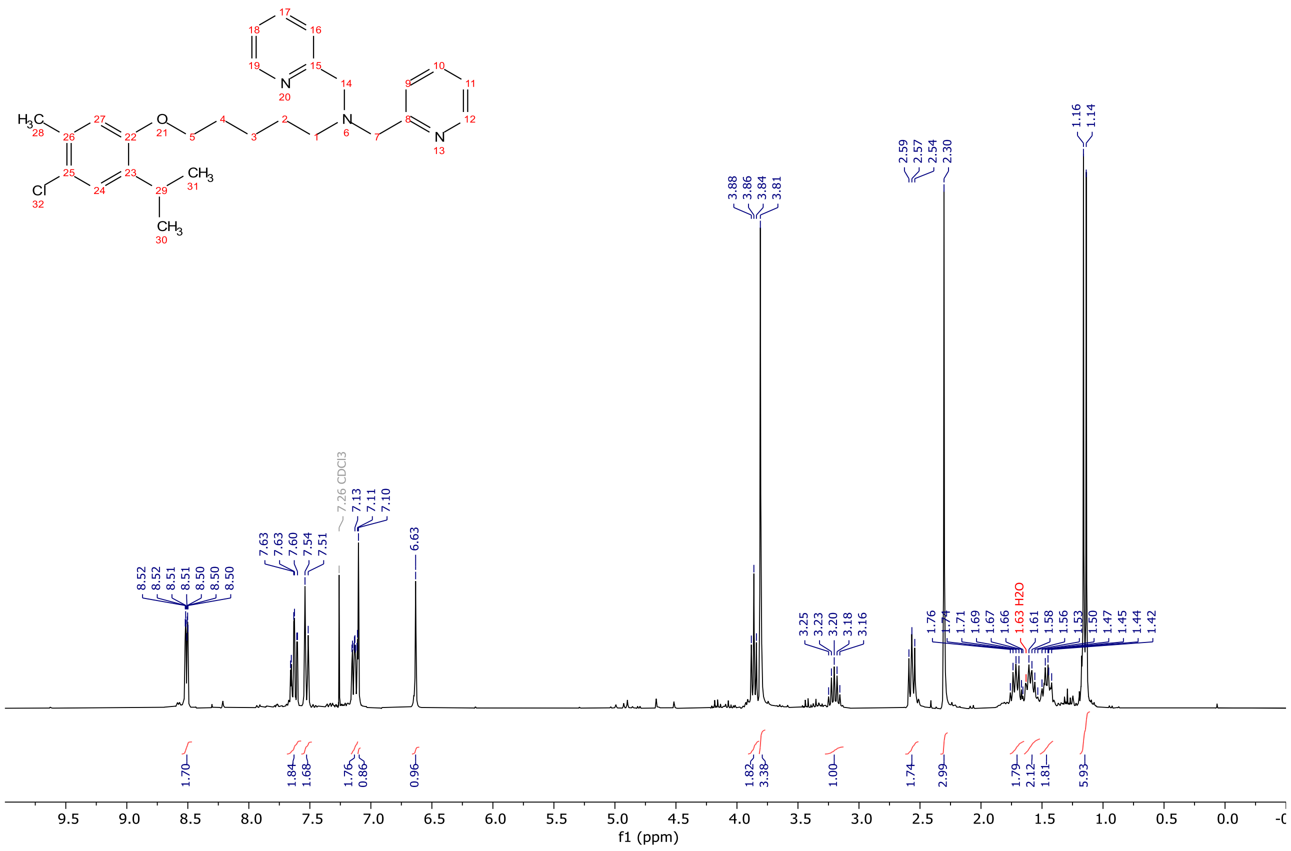

5-(4-chloro-2-isopropyl-5-methylphenoxy)-N,N-bis(pyridin-2-ylmethyl)pentan-1-amine (27).

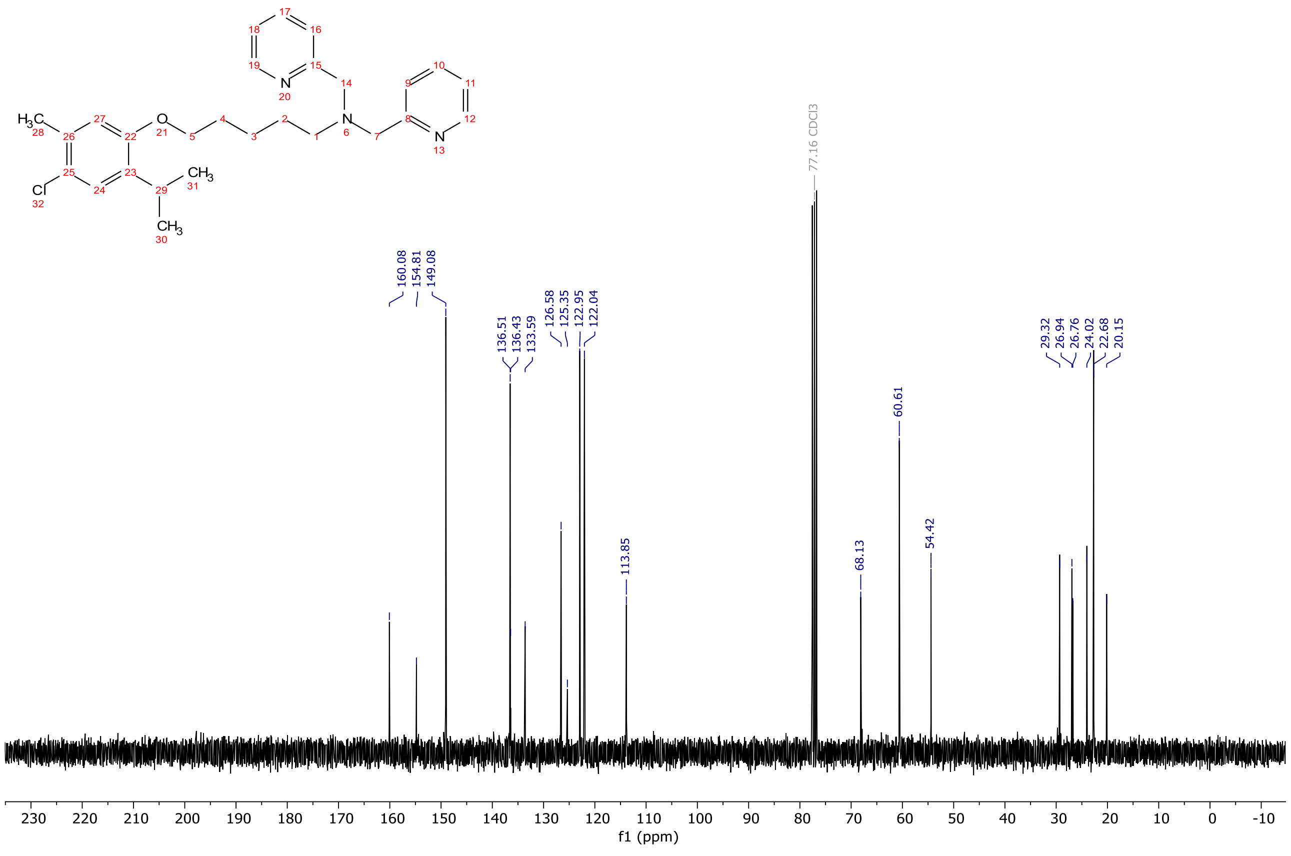

5-(4-isopropyl-3-methylphenoxy)-N,N-bis(pyridin-2-ylmethyl)pentan-1-amine (28).

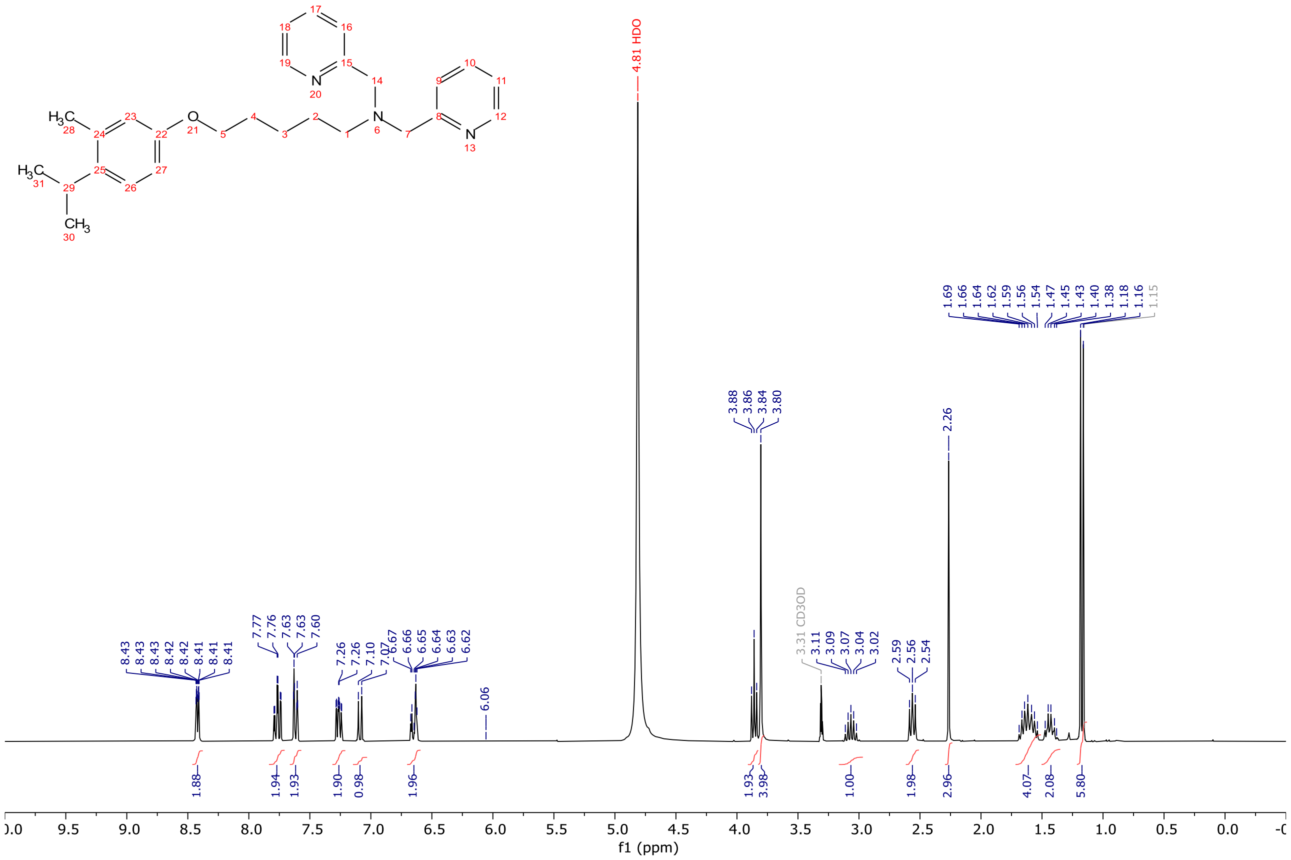

5-(4-isopropyl-3-methylphenoxy)-N,N-bis(pyridin-2-ylmethyl)pentan-1-amine (28).

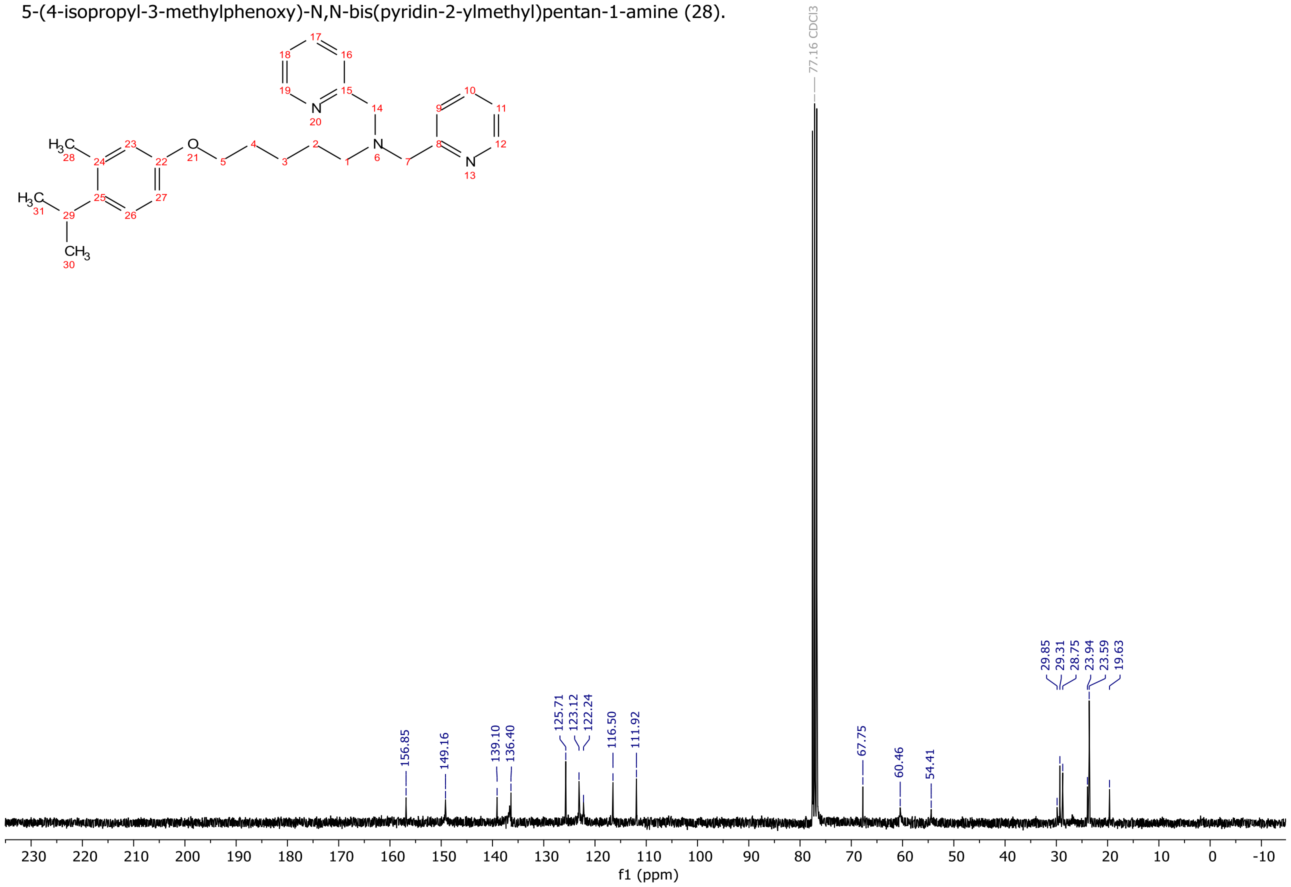

6-(4-chloro-2-isopropyl-5-methylphenoxy)-N,N-bis(pyridin-2-ylmethyl)hexan-1-amine (29).

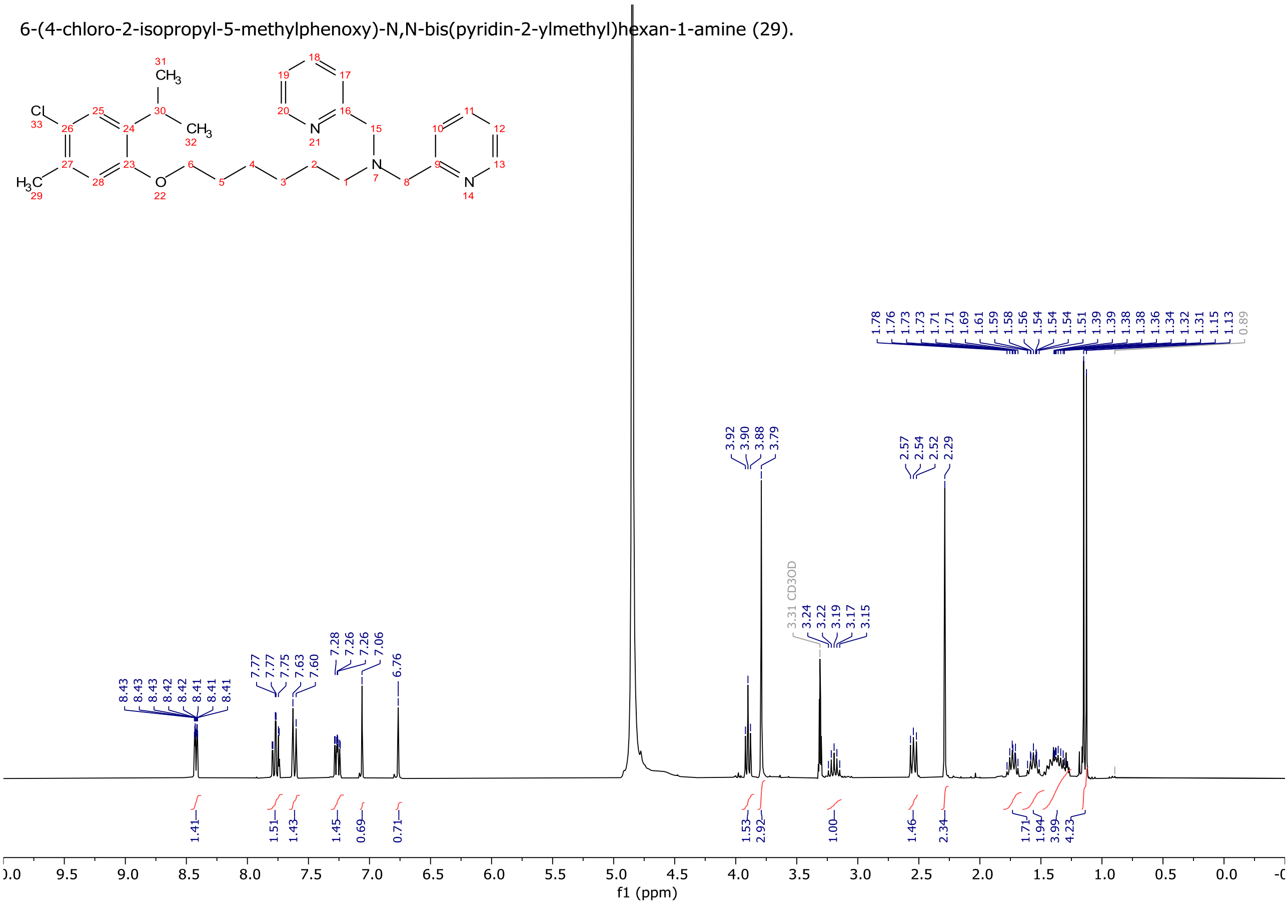

6-(4-chloro-2-isopropyl-5-methylphenoxy)-N,N-bis(pyridin-2-ylmethyl)hexan-1-amine (29).

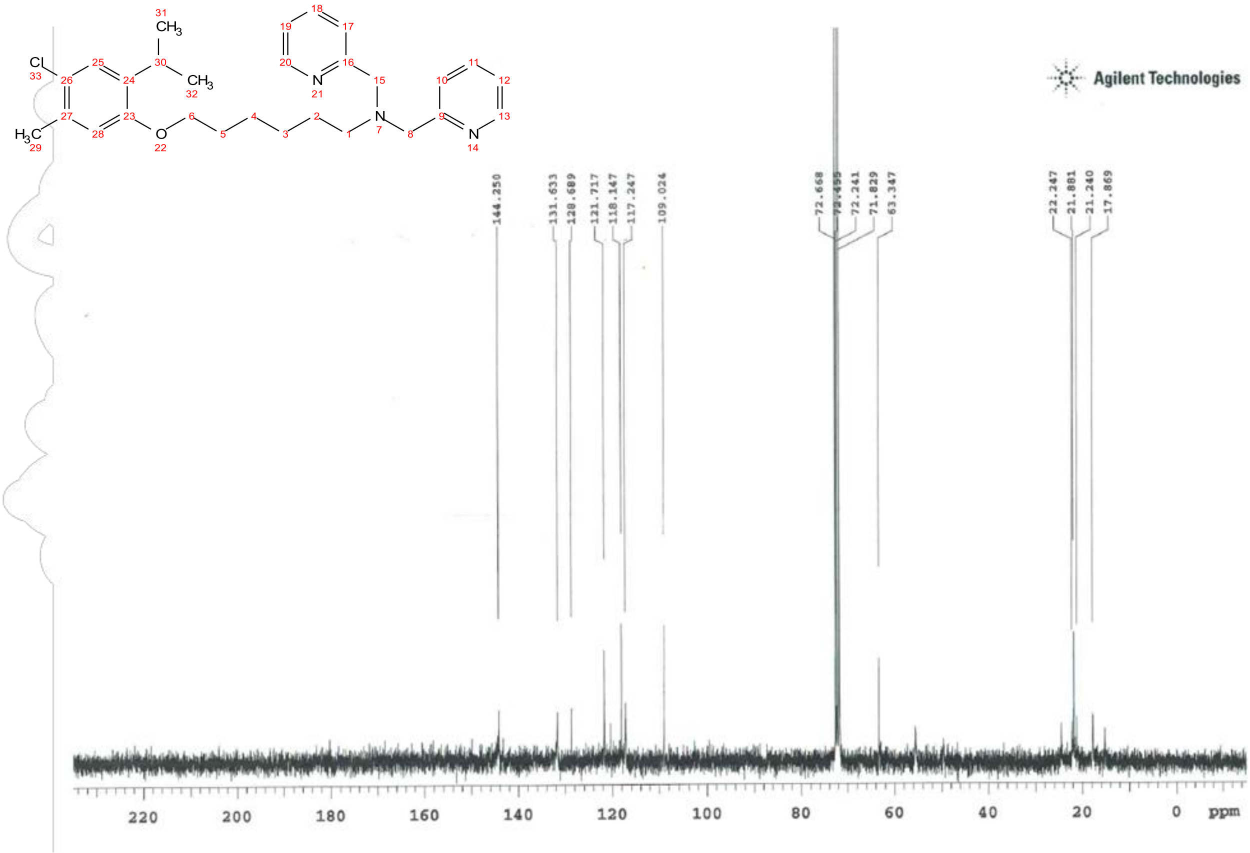

6-(4-isopropyl-3-methylphenoxy)-N,N-bis(pyridin-2-ylmethyl)hexan-1-amine (30).

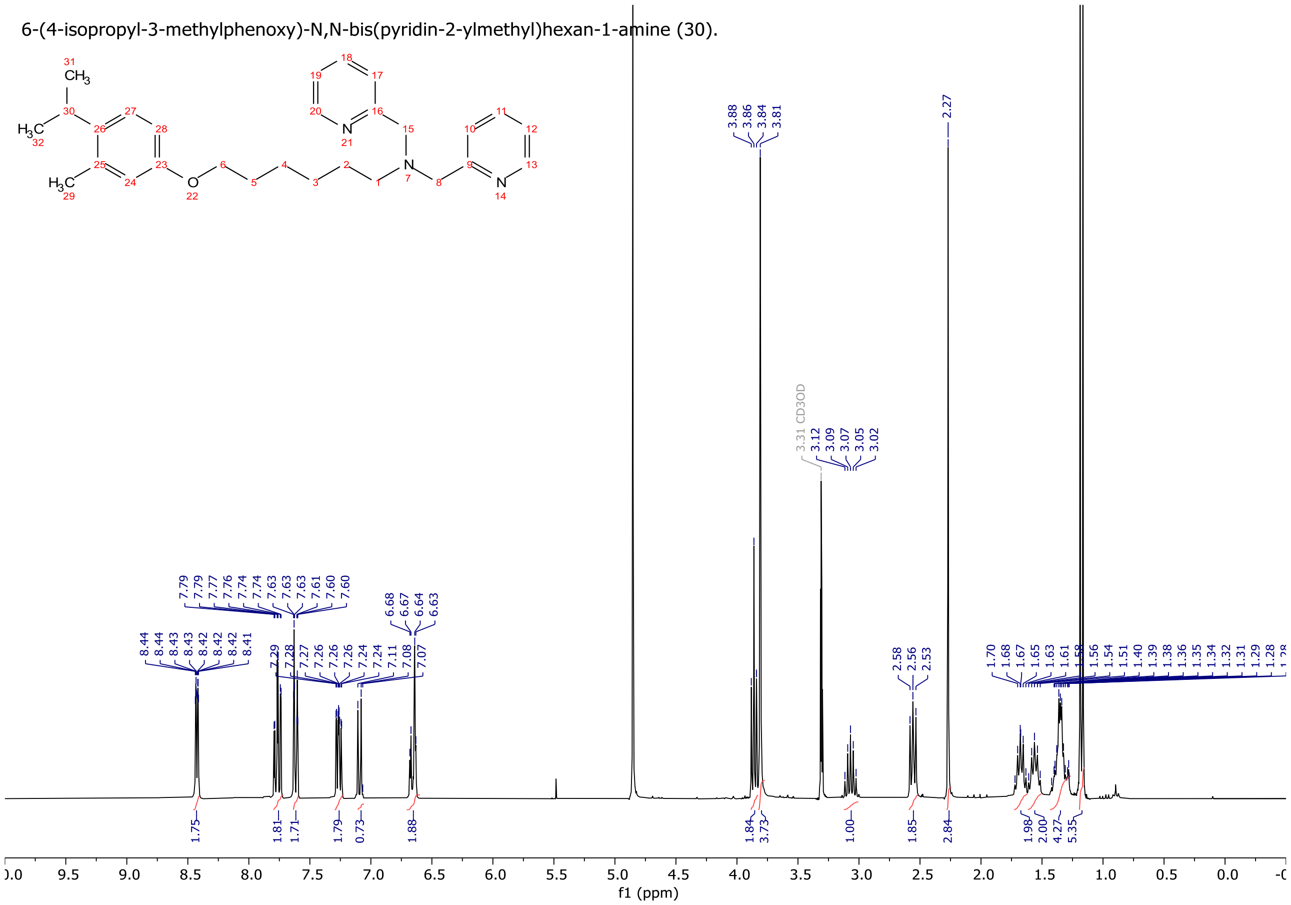

6-(4-isopropyl-3-methylphenoxy)-N,N-bis(pyridin-2-ylmethyl)hexan-1-amine (30).

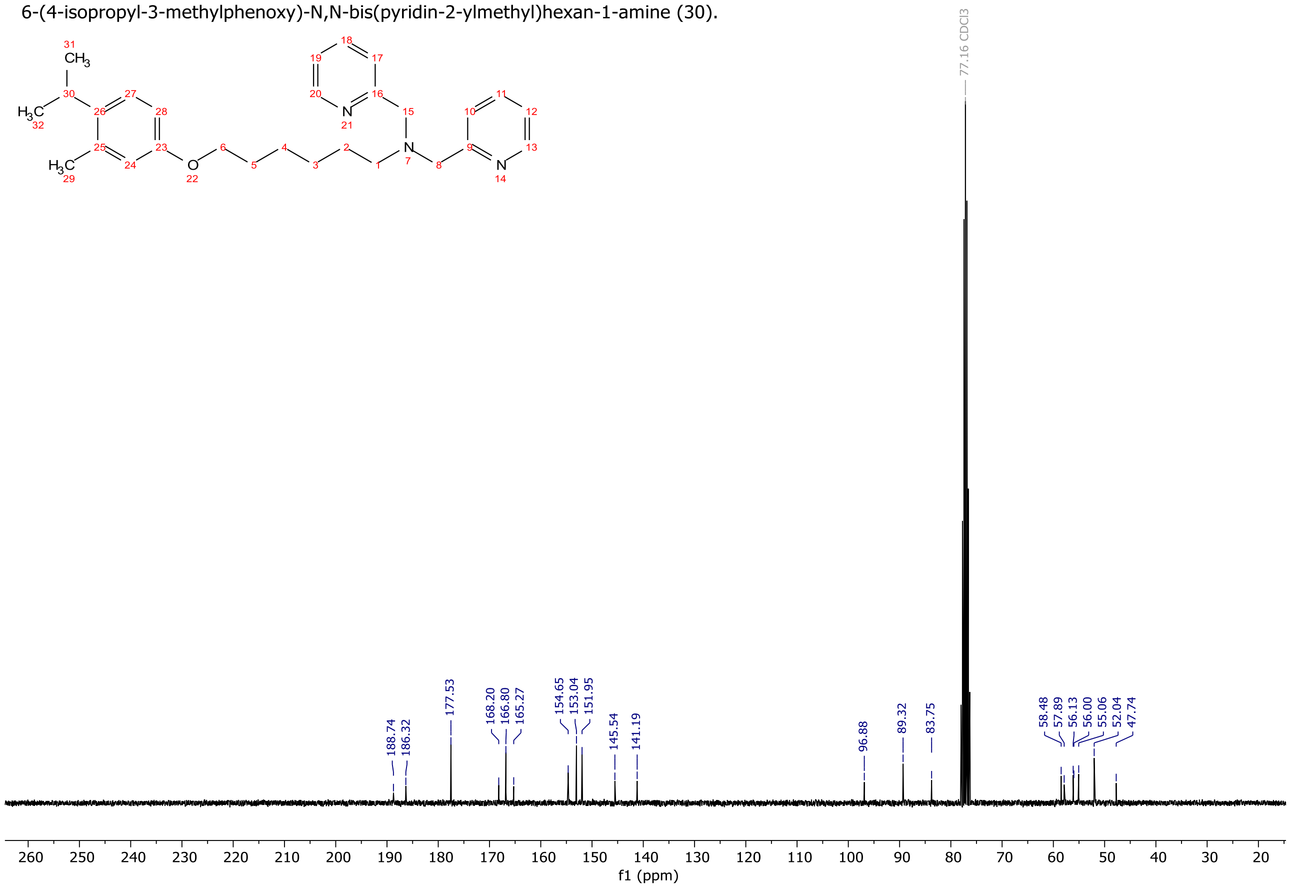

4-chloro-2-isopropyl-5-methylphenyl 2-oxo-2H-chromene-3-carboxylate (32).

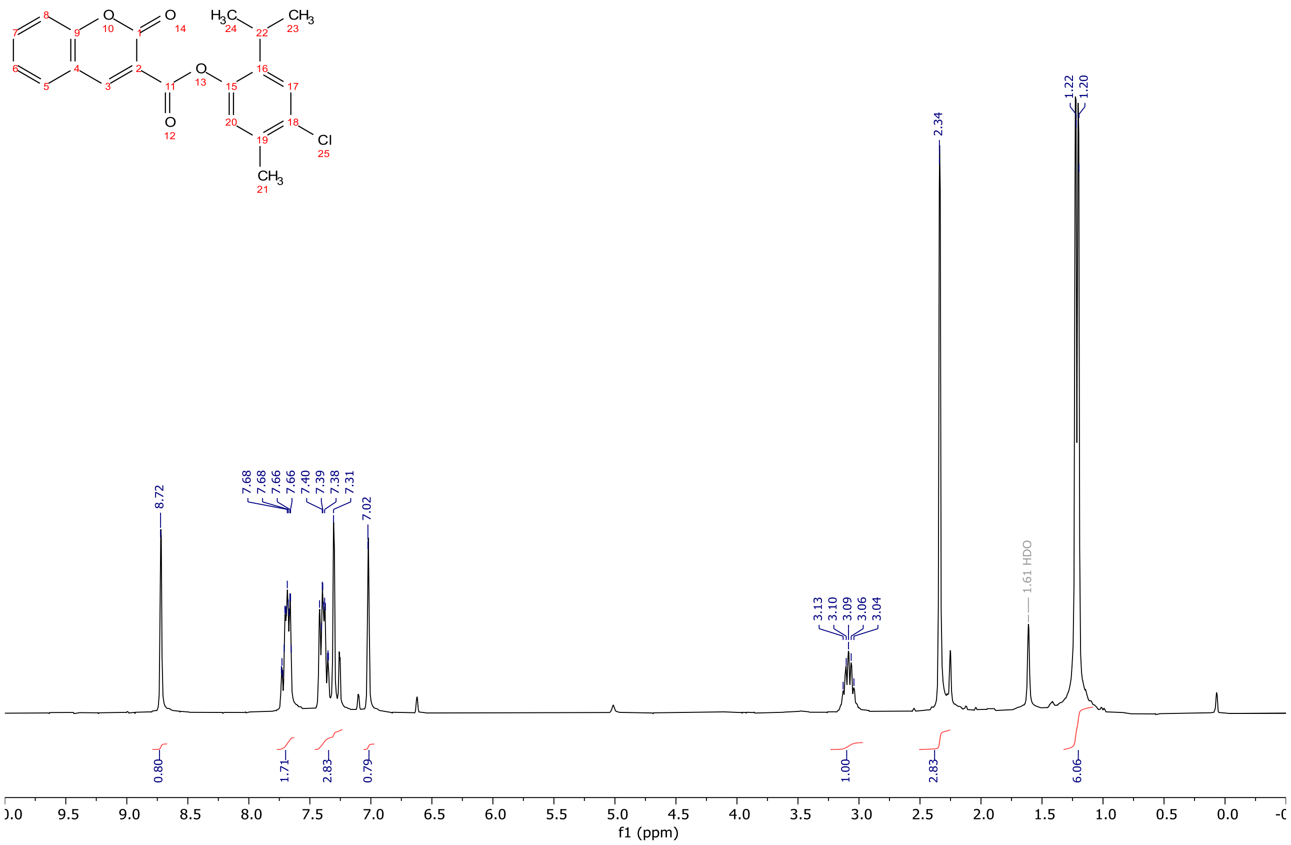

4-chloro-2-isopropyl-5-methylphenyl 2-oxo-2H-chromene-3-carboxylate (32).

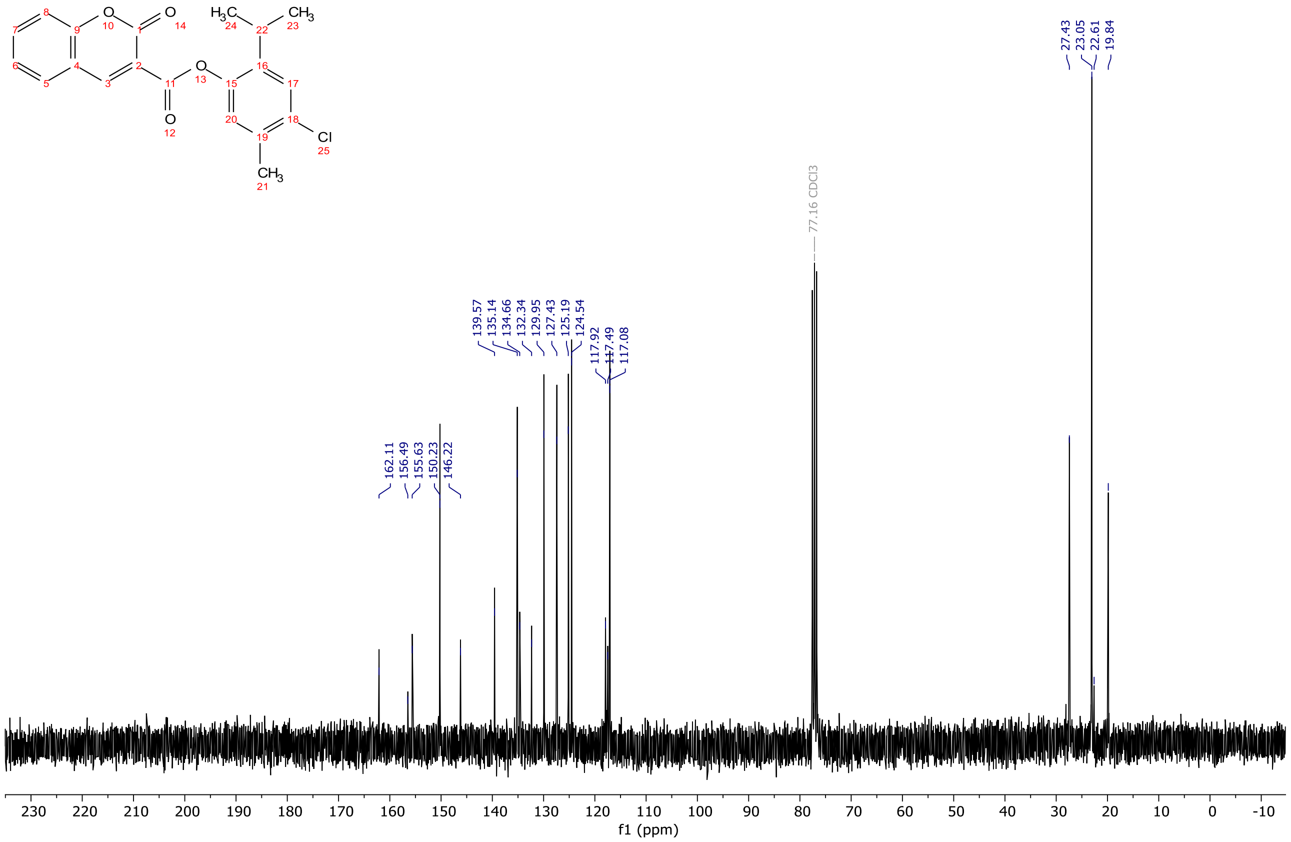

4-isopropyl-3-methylphenyl 2-oxo-2H-chromene-3-carboxylate (33).

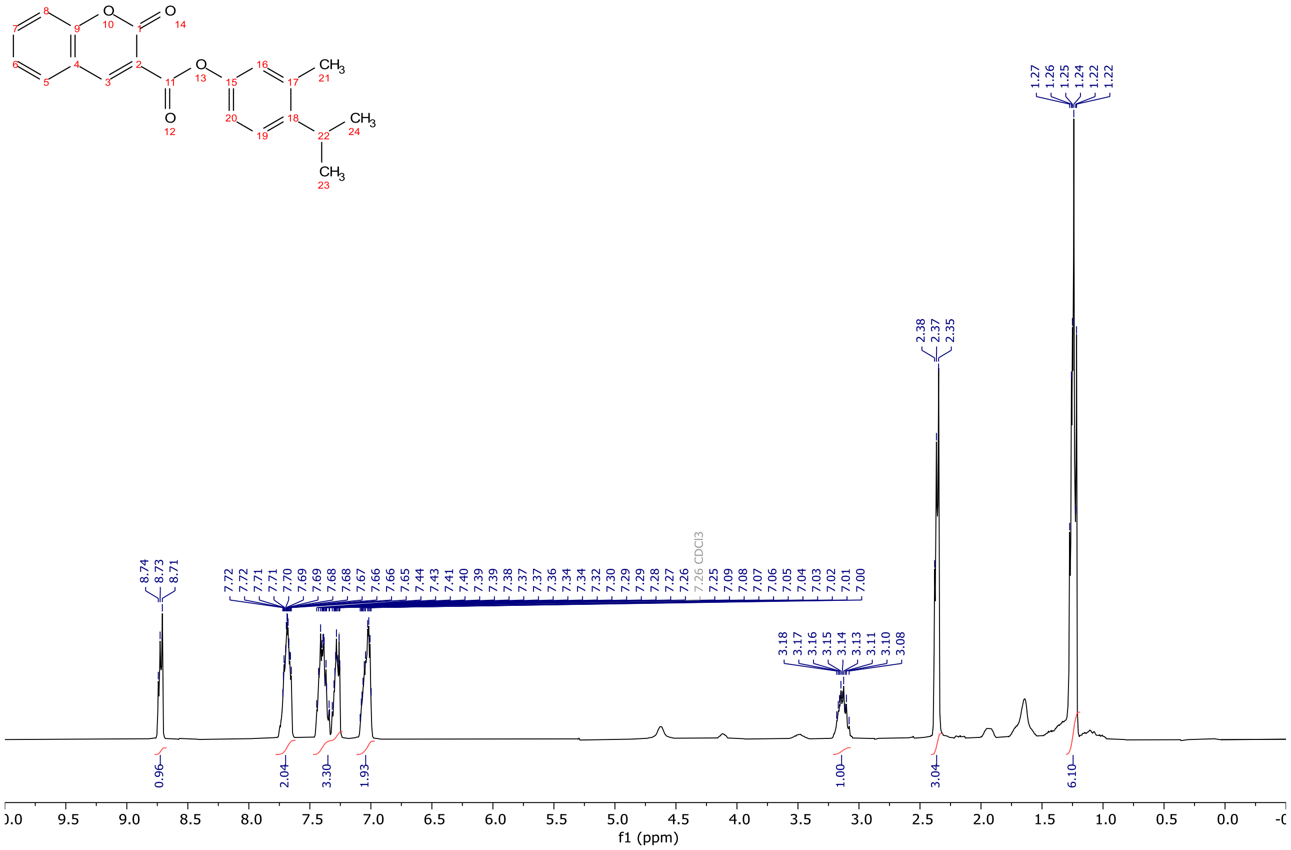

4-isopropyl-3-methylphenyl 2-oxo-2H-chromene-3-carboxylate (33).

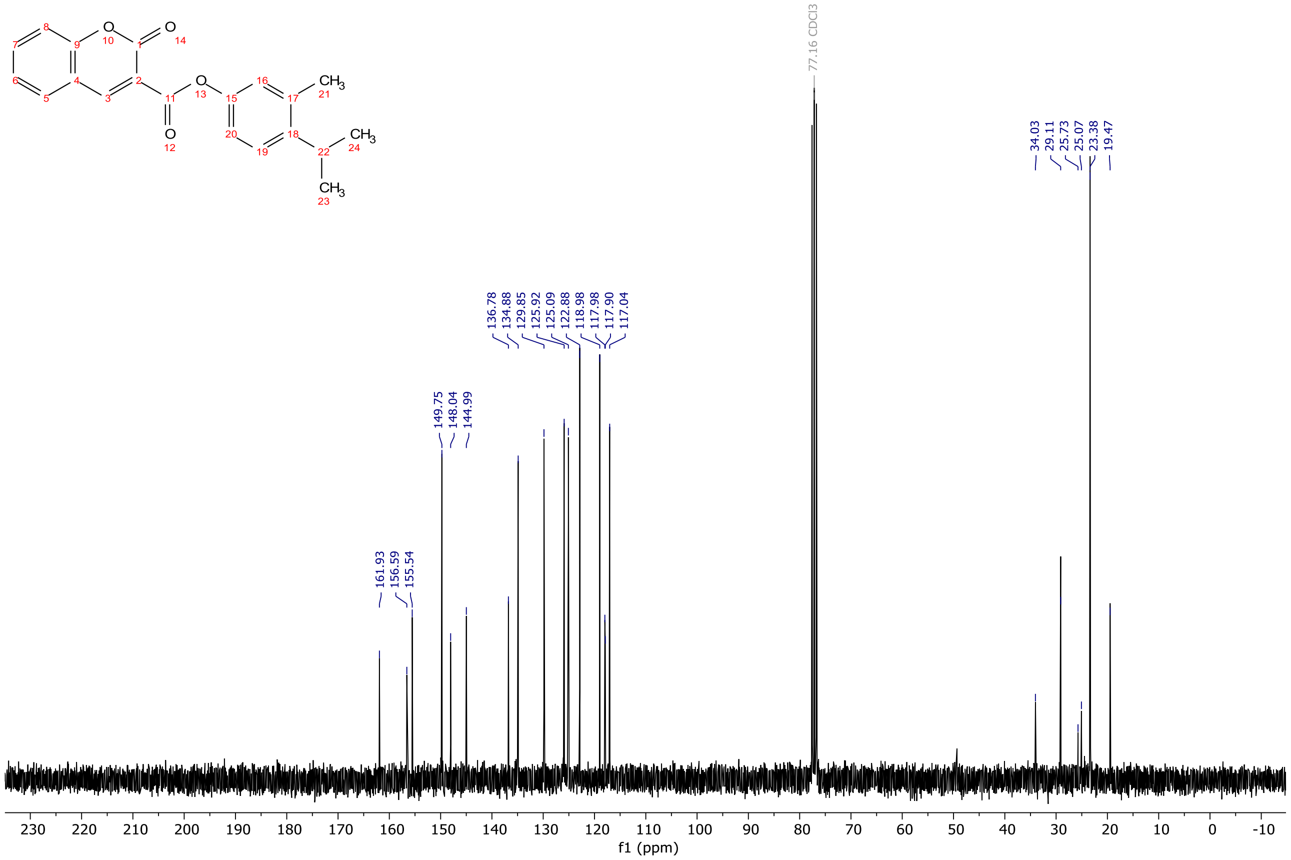

Supplement: Supplementary file 1 [file pharmaceuticals-19-00717-s001.zip › pharmaceuticals-4261487-supplementary.pdf]
